# Discovery of cahuitamycins as biofilm inhibitors derived from a convergent biosynthetic pathway

**DOI:** 10.1038/ncomms10710

**Published:** 2016-02-16

**Authors:** Sung Ryeol Park, Ashootosh Tripathi, Jianfeng Wu, Pamela J. Schultz, Isaiah Yim, Thomas J. McQuade, Fengan Yu, Carl-Johan Arevang, Abraham Y. Mensah, Giselle Tamayo-Castillo, Chuanwu Xi, David H. Sherman

**Affiliations:** 1 Life Sciences Institute, University of Michigan, Ann Arbor, Michigan 48109, USA; 2 Department of Environmental Health Sciences, University of Michigan School of Public Health, Ann Arbor, Michigan 48109, USA; 3 Unidad Estratégica de Bioprospección, Instituto Nacional de Biodiversidad (INBio), Santo Domingo de Heredia 223100, Costa Rica; 4 CIPRONA, Escuela de Química, Universidad de Costa Rica, 2060 San José, Costa Rica; 5 Department of Medicinal Chemistry, University of Michigan, Ann Arbor, Michigan 48109, USA; 6 Department of Chemistry, University of Michigan, Ann Arbor, Michigan 48109, USA; 7 Department of Microbiology and Immunology, University of Michigan, Ann Arbor, Michigan 48109, USA

## Abstract

Pathogenic microorganisms often have the ability to attach to a surface, building a complex matrix where they colonize to form a biofilm. This cellular superstructure can display increased resistance to antibiotics and cause serious, persistent health problems in humans. Here we describe a high-throughput *in vitro* screen to identify inhibitors of *Acinetobacter baumannii* biofilms using a library of natural product extracts derived from marine microbes. Analysis of extracts derived from *Streptomyces gandocaensis* results in the discovery of three peptidic metabolites (cahuitamycins A–C), with cahuitamycin C being the most effective inhibitor (IC_50_=14.5 μM). Biosynthesis of cahuitamycin C proceeds via a convergent biosynthetic pathway, with one of the steps apparently being catalysed by an unlinked gene encoding a 6-methylsalicylate synthase. Efforts to assess starter unit diversification through selective mutasynthesis lead to production of unnatural analogues cahuitamycins D and E of increased potency (IC_50_=8.4 and 10.5 μM).

## Abstract (web summary)

Pathogenic microbes can often attach to surfaces and form biofilms that display increased antibiotic resistance. Here, the authors characterize the biosynthesis of a new class of natural products, the cahuitamycins, that inhibit formation of biofilms by the pathogenic bacterium *Acinetobacter baumannii*.

## Graphical abstract

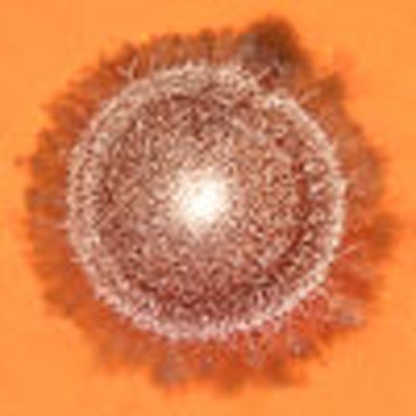

Widespread antibiotic resistance is currently posing a grave health burden through a multitude of serious infections^1^. The rise in bacterial adaptation can be directly correlated to the paucity of novel classes of antimicrobial agents^2^. In the past few decades, synthetic tailoring has been the primary strategy for enhancing established core scaffolds through analogue generation. Although this approach has been fruitful, no major classes of new antibiotics were introduced between 1962 and 2000 (ref. 3). Therefore, to restore robust access to effective therapeutic agents, it is imperative that we engage in aggressive efforts to discover novel chemical entities with unique microbial targets^2^,^4^.

*Acinetobacter baumannii* belongs to the ESKAPE group of major nosocomial opportunistic resistance pathogens (*Enterococcus faecalis, Staphylococcus aureus, Klebsiella pneumoniae, A. baumannii, Pseudomonas aeruginosa* and *Enterobacter* sp.), which can spread epidemically among patients causing ventilator-associated pneumonia and bacteremia, with mortality rates as high as 60%, representing a paradigm of pathogenesis, transmission and resistance^5^. Numerous reports have also shown startling emergence of multidrug resistant *A. baumannii* in hospitals, and also identification of pan-drug-resistant strains at some locations^5^,^6^. *A. baumannii* strains possess both intrinsic resistance to antibiotics and a facile ability to acquire genes encoding resistance determinants. In addition, antibiotic resistance of this pathogenic microbe appears to be mediated by their propensity to form biofilms with a highly structured extracellular polymeric matrix, and includes the ability to colonize medical devices. When attached, bacterial cells that comprise the biofilm possess 10–1,000-fold lower susceptibility towards antimicrobial agents compared with planktonic forms^7^. Moreover, despite the central role that bacterial biofilms play during infection, there are currently no drugs specifically targeting biofilms in clinical trials to date^8^,^9^. Therefore, a precision medicine is urgently required for vulnerable patients to avoid potential life-threatening infections. Recently, developing biofilm inhibitors has become a priority compared with biofilm disruption due to the advantage provided by preventing subsequent dispersion of cells that may have acquired drug resistance^10^,^11^,^12^. Although biofilm control by drug targeting has become a high priority objective^7^,^13^, marine microbes as a source of novel chemical entities remain relatively underexplored^14^,^15^.

In our continuing effort to identify new structural classes of antibiotics^4^, we employed static- and flow-based high-throughput screening (HTS) assays to survey our natural product extract (NPE) library in the search for new inhibitors of biofilm formation^16^. Here we describe the discovery of three novel secondary metabolites, whose stable production and full structural identification required ribosome engineering, and was facilitated by biosynthetic gene cluster characterization. In addition, we show that the cahuitamycins are derived from two independent starter unit pathways, one of which is genetically unlinked to the core cluster. The convergent pathway enabled us to perform directed pathway engineering to generate a more potent molecule, selectively. Furthermore, mutasynthetic efforts on the ribosomally modulated strain generated two additional novel compounds with enhanced activity against biofilm formation.

## Results and Discussion

### HTS for biofilm inhibitors against *A. baumannii*

In an effort to discover new antibiotics to combat hospital-associated infections in patients, we proceeded to screen our marine microbial-derived NPE library to identify biofilm inhibitors of *A. baumannii*. Natural products account for the majority of currently marketed drugs^17^, and the marine microbiome represents a potential new source of unique chemical entities^18^. We adapted a crystal-violet-based high-throughput assay^19^ that was queried against a library of 9,831 marine microbial-derived NPEs to identify the extracts inhibiting biofilm formation as the primary screen. The active extracts were further prioritized by setting the inhibition threshold to 50% followed by a dose–response assay, yielding 31 active NPEs (Supplementary Figs 1–2). A second round of microbial biological activity analysis was conducted on the top nine most potent extracts (Supplementary Fig. 2). This study revealed the extract from *Streptomyces gandocaensis* to be of particular interest due to its ability to inhibit biofilm formation, but showing a limited effect on *A. baumannii* growth (Supplementary Figs 1–3).

### Ribosome engineering of *S. gandocaensis*

Following initial identification of the active principles, regrowth of the wild-type *S. gandocaensis* over several months showed complete loss of production of the active biofilm inhibitor molecules. Therefore, we decided to immediately pursue a ribosome engineering approach to restore and improve production of the active metabolites. This approach has been employed for activation of secondary metabolite production in *Streptomyces* spp.^20^, and can result in significantly enhanced yields by inducing point mutations in ribosomal protein-encoding genes (for example, *rpsL*). Several rounds of mutagenesis based on a streptomycin resistance phenotype resulted in an improved strain DHS287 of *S. gandocaensis* (Supplementary Fig. 4; Supplementary Table 7), a fourth-generation mutant with restored stable production that generates several-fold increased quantities of active molecules compared with initial wild-type levels. Genetic analysis revealed that the streptomycin-induced ribosome engineering introduced a point mutation in the *rpsL* gene, which encodes the ribosomal protein S12, in the engineered strain (Supplementary Fig. 5; Supplementary Table 3). Previous studies have shown that mutations in the *S12* gene render cells potentially more active for polypeptide synthesis under typical starvation conditions during the late growth phase^20^. This effort appears to be the first reported instance where complete loss of an active, but structurally uncharacterized natural product has been recovered using the ribosome engineering approach.

### Isolation and structure elucidation of cahuitamycins A–C

**Figure 1:**
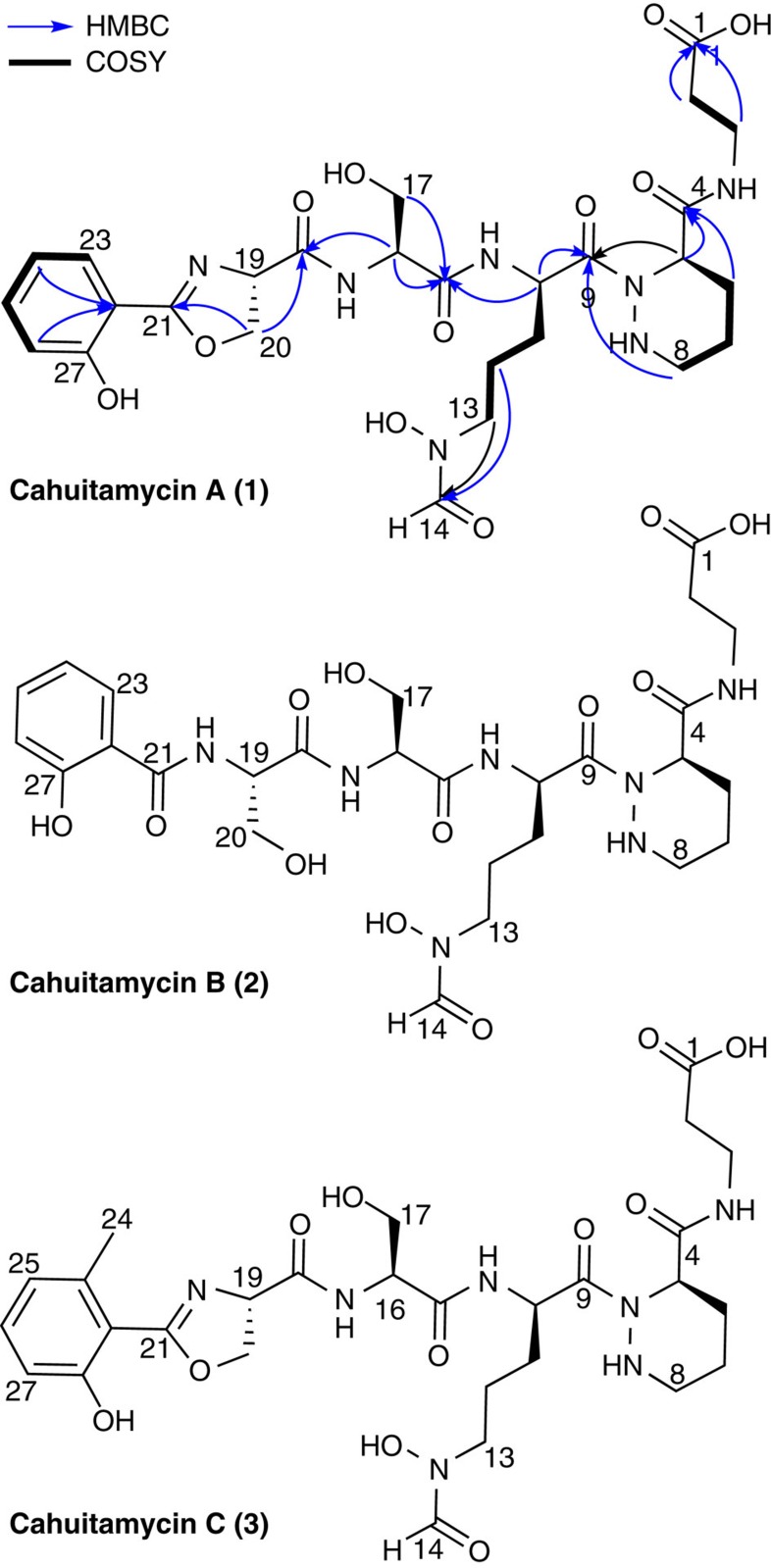
Structures of cahuitamycins A–C (**1**–**3**) with absolute stereochemistry. Cahuitamycin A shows key HMBC and COSY correlations.

Bioassay guided C18 column fractionation followed by high-performance liquid chromatography (HPLC) purification of organic extracts obtained from the ribosome-engineered *S. gandocaensis* DHS287 yielded three new secondary metabolites, cahuitamycins A–C (**1**–**3**; Fig. 1). Cahuitamycin A (**1**), the major metabolite, showed a high-resolution time-of-flight electrospray ionization mass spectrometry (ESIMS) [M+H]^+^ ion peak at *m/z* 636.2679, indicating the molecular formula of C_27_H_37_N_7_O_11_ (+0.3 p.p.m.) requiring 13° of unsaturation (Supplementary Fig. 8). The one-dimensional (1D; ^1^H, ^13^C) and two-dimensional (2D; gHSQCAD, gHMBCAD and gCOSY) NMR data acquired in CD_3_OD+D_2_O (4:1) indicated the peptidic nature of **1** (Supplementary Figs 9–13) by the presence of 8 methyl/methine carbons, 10 methylene carbons and 9 carbonyls/quaternary carbons. Analysis of gCOSY, TOCSY and gHMBC cross peaks at δ_H_ 7.01, 7.48, 6.97 and 7.71 to δ_C_ 159.7 and 159.8 suggested the spin system consisting of an *ortho*-substituted phenol group. In addition, correlations observed through long-range ^1^H–^13^C between δ_H_ 4.69 (H-20a) and δ_C_ 168.6 (C-21) as well as ^1^H–^1^H between δ_H_ 5.11 (H-19) and 4.61 (H-20b) interactions indicated the moiety to be a *N*-terminal 2-phenyl-oxazoline group. Further analysis of the gCOSY and TOCSY spectra indicated at least four more spin systems consisting of a serine (Ser), two modified ornithines (Orns) and a modified alanine (Ala) (Supplementary Figs 8–14). We defined modified Orn as *N*^δ^-hydroxy-*N*^δ^-formylornithine (*N*-OH-*N*-fOrn) based on the COSY relay observed between δ_H_ 4.29 (H-10), 1.82 (H-11), 1.68 (H-12), 3.45 (H-13) and a gHMBC correlation between H-13 and C-14 (δ_C_ 164.1). Similarly, another Orn-like spin system potentially related to a piperazic acid (Pip) based on long-range ^1^H–^13^C between δ_H_ 3.57, 3.63 (H-8)-δ_C_ 173.9 (C-9) and a short-range ^1^H–^1^H array observed from H-5 to H-8 (Fig. 1). The C terminus of the peptide was identified as β-alanine (β-Ala) based on COSY correlation between H-3 (δ_H_ 3.47, 3.39) to H-2 (δ_H_ 2.39) and an HMBC correlation from H-3 to C-1 (δ_C_ 174.1). All deduced moieties completed the planar structure of **1** (Fig. 1; Supplementary Table 1).

Cahuitamycin B (**2**) was isolated by RP-18 HPLC from the same C18 fraction containing compound **1**. The high-resolution ESIMS (HRESIMS) [M+H]^+^ ion peak at *m/z* 654.2759 provided a molecular formula of C_27_H_39_N_7_O_12_ with only 12° of unsaturation compared with 13 in **1** (Supplementary Fig. 15). Moreover, the 1D NMR data although acquired in dimethylsulphoxide (DMSO)-d_6_, showed high structural similarity to **1** with the existence of six carbonyls (between δ_C_ 164.8 and 171.1), an aldehyde (δ_C_ 161.6, δ_H_ 8.21) and a phenyl group functionality (between δ_C_ 116.7–156.9 and δ_H_ 6.81–7.91) fulfilling 11 out of 12 degrees of unsaturation. Analysis of the 2D NMR data for **2** suggested a similar carbon backbone as **1** except the gCOSY and HMBC cross peaks at δ_C_ 55.4, δ_H_ 4.56 (C-16)—δ_C_ 61.5, δ_H_ 3.58, 3.63 (C-17) and δ_C_ 55.2, δ_H_ 4.52 (C-19)—δ_C_ 61.7, δ_H_ 3.69, 3.73 (C-20) indicating two Ser groups, replacing the ring in **1**, respectively (Fig. 1; Supplementary Figs 16-20). Furthermore, similar COSY and HMBC correlation as observed in **1** suggested the presence of Pip, thus accounting for the 12th degree of unsaturation to complete the structure of **2** (Supplementary Table 1).

Cahuitamycin C (**3**) was also isolated as a white amorphous solid from the same C18 fraction containing **1** and **2**. The HRESIMS [M+H]^+^ ion peak at *m/z* 650.2804 provided a molecular formula of C_28_H_39_N_7_O_11_ with 13 degrees of unsaturation (Supplementary Fig. 21). Extensive 1D and 2D NMR analysis indicated that **3** shares structural similarity on much of the carbon backbone compared with **2** (Supplementary Table 1). The only observed difference was localized to the phenyl ring system where an HMBC correlation from δ_H_ 2.51 (H-24) singlet to δ_C_ 123.1 (C-25) and 112.8 (C-22) suggested methylation of the *N*-terminal 2-hydroxybenzoyl-oxazoline group at C-23 (δ_C_ 141.2). Furthermore, a change in ^1^H multiplicity at δ_H_ 6.73 (H-25) to a doublet confirmed the planar structure of **3** (Fig. 1; Supplementary Figs 22–26).

### Stereochemical studies for cahuitamycins A–C

Due to the polypeptidic nature of the cahuitamycins, we pursued advanced Marfey's analysis^21^,^22^ to ascertain absolute stereochemistry. Initially, only cahuitamycin B (**2**) was selected for acid hydrolysis followed by 1-fluoro-2,4-dinitrobenzene-5-alanine amide (FDAA) derivatization. This was based on **2** appearing to be the congener leading to production of cahuitamycins A and C (**1** and **3**) following cyclization or vice versa (see further below).

A study was conducted to compare *m/z* 357.27, 382.32 and 400.34 channels from liquid chromatography/electrospray ionization/mass spectrometry (LC-ESI-MS) chromatograms between the L-FDAA and D, L-FDAA-derivatized Ser, Pip and *N*-OH-Orn (a hydrolysed product of *N*-OH-*N*-fOrn) products of **1**, respectively. Analysis clearly revealed the absolute configuration of the moieties in the hydrolysate of **1** to be L-Ser, L-Ser, D-Pip and D-*N*-OH-Orn, respectively (Fig. 1).

**Figure 2:**
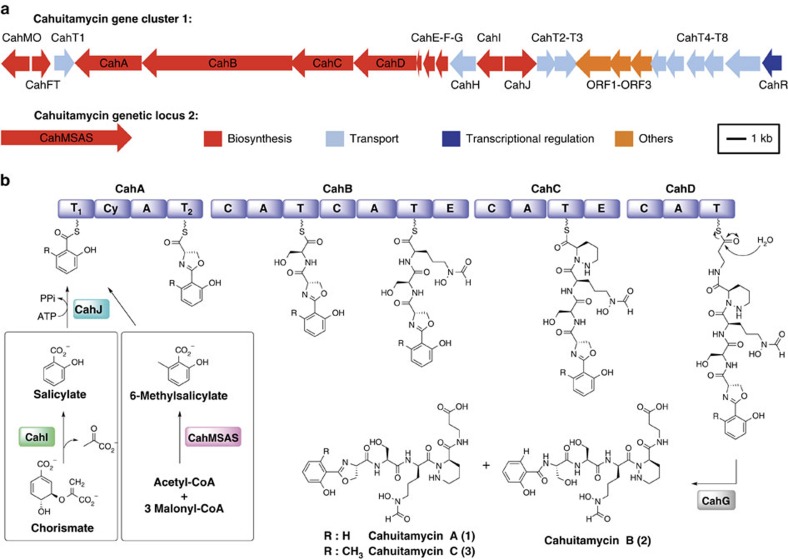
Complete biosynthetic scheme of cahuitamycins. Organization of the bifurcated cahuitamycin (*cah*) gene cluster (**a**) and a proposed biosynthetic pathway of cahuitamycins **1**–**3** (**b**). Domain notation: A, adenylation; C, condensation; Cy, cyclization; E, epimerization; T, thiolation.

Furthermore, analysis of the Ser, Pip and *N*-OH-Orn portions of **1** and **3** was conducted as described above for **2**, revealing the same stereochemistry. The absolute configuration of the oxazoline ring in **1** and **3** was extrapolated to be D based on similar NMR chemical shifts compared with **2,** as well as the absence of an epimerization domain in the first module (CahA) of the cahuitamycin biosynthetic gene cluster (see above, Fig. 2).

### Affinity of cahuitamycins towards iron

Cahuitamycins were observed to have siderophore-like properties, and we therefore conducted competition titrations with EDTA to identify their relative iron-binding affinities^23^. In this method, the Fe–cahuitamycin complex was incubated with varying concentrations of EDTA and assessed for Fe distribution using ultraviolet–visible spectroscopy (Methods). The *pFe*^III^ for cahuitamycins were calculated against the known stability constants for EDTA (*pFe*^III^=23.42)^24^. Three individual titrations were conducted for each natural product, and the representative *pFe*^III^ values for cahuitamycins **1**–**3** were measured to be 18.34±0.16, 20.42±0.09 and 17.52±0.12, respectively (Supplementary Fig. 38). Importantly, the calculated *pFe*^III^ for **1**–**3** was significantly lower than those previously reported for hydroxamate siderophores under similar physiological condition (∼25–37)^25^. This supports our hypothesis that cahuitamycins possess siderophore properties, but their relatively low affinity may indicate it to be a secondary endogenous function. Interestingly, the diminishing activity of **1** over an extended bioassay time course and the observed inverse proportionality between *pFe*^III^ of **1**–**3** against *A. baumannii* biofilm inhibition could be attributed to this iron chelation property (see below).

### Dissecting the cahuitamycin biosynthetic gene cluster

To mine a candidate gene cluster for cahuitamycin biosynthesis, analysis of the draft genome sequence of *S. gandocaensis* was performed using antiSMASH^26^. As a result, a gene cluster was identified (GeneBank accession code KU363800) as potentially responsible for nonribosomal peptide scaffold biosynthesis, transport and regulation of cahuitamycins A and B (**1** and **2**; Fig. 2a). Cahuitamycin C (**3**) biosynthesis was initially expected to involve a *S*-adenosyl-L-methionine-dependent C-methyltransferase through post-assembly tailoring. However, the absence of genes responsible for the formation of a methyl group at C-23 position of **3** (Fig. 1) near cluster 1 led to the hypothesis that another genetic locus encoding 6-methyl salicylate synthase (*6-MSAS*) might be involved in its biosynthesis (Fig. 2). The architecture and annotation of the bifurcated cahuitamycin (*cah*) gene cluster is shown in Fig. 2 and Supplementary Table 4, respectively. The cluster containing nonribosomal peptide synthetase (NRPS)-encoding genes *cahA–D*, together with genes involved in chain initiation (*cahIJ*), termination (*cahG*) and a transcriptional regulator (*cahR*), is located in a region spanning ∼40 kb of DNA. Each NRPS protein consists of the essential condensation (C), adenylation (A) and thiolation (T) domains (Fig. 2b), and has a non-colinear architecture.

CahA is proposed to catalyse oxazoline ring formation together with a putative salicylate synthase CahI and salicylate-AMP ligase CahJ (Fig. 2b). CahI is homologous to MbtI from *Mycobacterium tuberculosis* and is predicted to convert chorismate to salicylate (Fig. 2b)^27^. CahJ bears high similarity to a salicylate-AMP ligase MxcE from *Sorangium cellulosum* So ce56 (ref. 28), which suggests that it activates salicylate by adenylation (Fig. 2b). The identity of amino acid building blocks selected and activated by the Cah NRPS were analysed by examining the specificity-conferring residues in each A domain (Supplementary Table 5)^29^. The A domain in CahA is predicted to recognize L-Cys (Supplementary Table 5), though only L-Ser is loaded according to the structure of cahuitamycins (Fig. 1), which is also observed in the amychelin biosynthetic system^30^. The heterocyclization between Ser and the carbonyl group to form an oxazoline ring scaffold can be attributed to the Cy domain in CahA, and the resulting peptide product is then transferred to CahB for synthesis of **1** and **3**. Similar to formation of gobichelins, where the oxazoline ring of gobichelin A is hydrolysed into a linear form under non-acidic condition, **1** may convert to **2** with a free Ser through hydrolysis of the oxazoline ring^31^. The *in silico* analysis of A domains in *CahB* predicts activation of L-Ser and L-*N*-OH-*N*-fOrn by CahB-A_1_ and CahB-A_2_, respectively (Supplementary Table 5). Subsequent to the incorporation of L-Ser by CahB-A_1_, a L-*N*-OH-*N*-fOrn moiety, which is synthesized from L-Orn by a putative Orn hydroxylase (CahMO) and a putative *N*-formyltransferase (CahFT)^32^, is appended to the growing chain (Supplementary Table 4). The L-*N*-OH-*N*-fOrn moiety linked to the T_2_ domain of CahB undergoes epimerization by the epimerase (E) domain^33^, which is consistent with the stereochemistry of cahuitamycins (Fig. 1; Supplementary Figs 8–37).

Biosynthesis of cahuitamycins likely involves formation and loading of the piperazic acid (Pip) building block into the growing peptide chain. Although the relatively rare Pip moiety is found in several biologically active secondary metabolites^34^,^35^,^36^,^37^,^38^,^39^, the precise biosynthetic events for Pip elaboration have not been elucidated. Recent precursor incorporation experiments have suggested that generation of the Pip moiety in kutzneride occurs before incorporation into the respective peptide chain^35^, similar to the formation of a hydrazo linkage in valanimycin^34^. It is likely that biosynthesis of the Pip moiety in cahuitamycin is initiated by *N*-hydroxylation of the amino group of Orn by a putative CahMO. The resulting L-*N*-OH-Orn residue attached to the T domain of CahC would be converted to its corresponding D-stereoisomer (D-*N*-OH-Orn) by the E domain within the same module followed by nucleophilic attack of the amino group to close the ring. Alternatively, since the A domain in the CahC is homologous to L-glutamine (Gln)-activating A domains (Supplementary Table 5), it is possible that Gln may be incorporated as a branch point from primary metabolism into the piperazate biosynthetic pathway (Fig. 2)^40^,^41^.

Further *in silico* analysis of the A domain in CahD (Supplementary Table 5) predicted loading of β-Ala onto the T domain consistent with the structure of cahuitamycins. Moreover, it further affirms that the presence of β-Ala residue is likely formed from L-aspartate (L-Asp) by the *cahF* gene product^42^. The CahD polypeptide contains neither C-terminal thioesterase nor a reductase domain usually required for chain release in nonribosomal peptide biosynthesis, and likely involves a specific hydrolase for release of the fully assembled peptide chain from the T domain of the last module CahD. We propose that following final extension by β-Ala, CahG (α/β hydrolase superfamily) catalyses in trans release of the peptide chain through hydrolysis of the T domain-bound thioester as described previously in coelichelin biosynthesis^43^.

Moreover, CahE, an MbtH-like protein homologue, likely contributes to the stimulation of A domains in the Cah NRPS^44^. CahH, which belongs to the major facilitator superfamily, is similar to EntS, an enterobactin efflux exporter of *Escherichia coli*. Proteins encoded by CahT1 through CahT8 are putatively required for iron uptake and translocation of cahuitamycin across the cell membrane (Supplementary Table 4). Therefore, these proteins might be involved in the export of cahuitamycins from the cytoplasm into the medium. Open reading frame 1 (ORF1), ORF2 and ORF3 show sequence similarity to oxidoreductase, acyltransferase and 6-aminohexanoate-oligomer hydrolase, respectively, and their relationship to cahuitamycin biosynthesis remains unclear (Supplementary Table 4).

### Biosynthesis of cahuitamycin C

**Figure 3:**
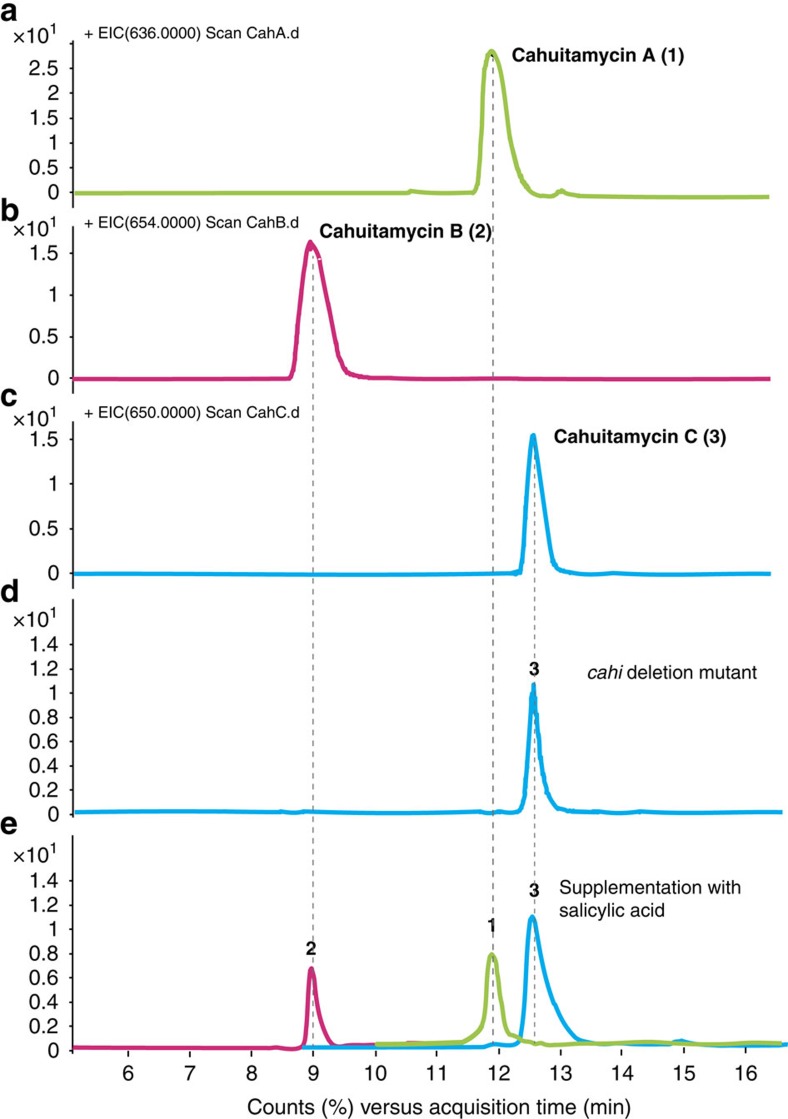
Chemical complementation of the *cahI* deletion mutant of *S. gandocaensis*. LC-ESI-MS chromatograms of **1**–**3** obtained from culture of DHS287 (**a**–**c**) the *cahI* deletion mutant DHS334 (**d**) supplemented with 100 μM of salicylic acid (**e**).

We next sought to confirm the function of the assigned biosynthetic cluster by deletion of *cahI* in *S. gandocaensis* through insertion of the kanamycin resistance gene (*aph*). Surprisingly, the Δ*cahI S. gandocaensis* strain DHS334 (Methods; Supplementary Fig. 7; Supplementary Table 7) gave exclusive production of **3**, while the production of **1** and **2** were completely abolished (Fig. 3d). The result indicated a possible unlinked source involved in providing 6-methylsalicylate starter unit. A BLAST analysis using bacterial 6-MSASs including various putative candidates from other bacterial secondary metabolite gene clusters^45^,^46^,^47^,^48^,^49^, led to the identification of a single iterative type I PKS gene encoding a 6-MSAS (genetic locus 2; GeneBank accession code KU363801) located at least ∼50 kb away from cluster 1 based on our *S. gandocaensis* draft genome-sequencing data (Fig. 2a). This finding led us to propose that the 6-methylsalicylate starter unit is likely derived from the 6-MSAS identified from bioinformatics mining, which is subsequently adenylated by CahJ and loaded onto the T_1_ domain of CahA for production of **3** (Fig. 2). As in other bacterial PKSs in its class, the putative cahuitamycin 6-MSAS (genetic locus 2; *cah MSAS*) possesses keto synthase, acyltransferase, dehydratase, ketoreductase and acyl carrier protein domains on a single protein (Supplementary Table 4). The hypothesis was further substantiated by the dosing effect observed by introducing exogenous 6-methylsalicylic acid into cultures of the Δ*cahI S. gandocaensis* strain. The feeding study provided increased production of **3**, while no changes were observed in production of **1** and **2** (Supplementary Fig. 39). This result, together with the lack of other potential sources of 6-methylsalicyclate, or a candidate methyltransferase encoded within the *S. gandocaensis* genome appears to rule out the possibility of any posttranslational modification of **1** to produce **3**. Furthermore, production of **1** and **2** was restored by external supplementation of salicylic acid to the Δ*cahI* growth medium (Fig. 3e), representing chemical complementation and confirmation of its role in the production of cahuitamycins. These studies substantiated that biosynthesis of **3** is independent of *cahI*-mediated salicylic acid biosynthesis, and more importantly cahuitamycin C (**3**) appears to be derived from a convergent biosynthetic pathway (Fig. 2). Secondary metabolites assembled from unlinked gene clusters have been reported in previous studies^32^,^50^,^51^, but to the best of our knowledge this is the first example of a starter unit pathway that is genetically unlinked to a corresponding bacterial NRPS. The observation provides an intriguing example where *S. gandocaensis* may be diversifying its biosynthetic machinery to produce a more potent/toxic metabolite for enhanced competitiveness in its natural habitat.

### Mutasynthetic generation of cahuitamycin analogues

**Figure 4:**
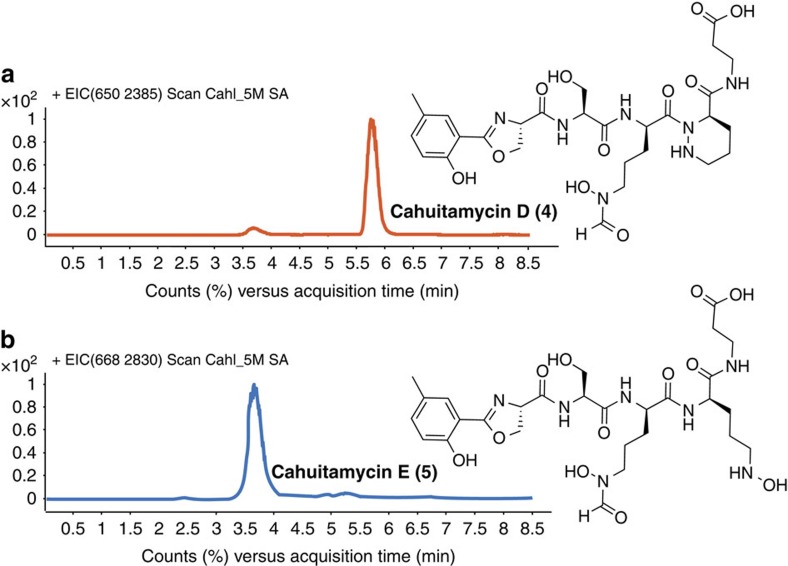
Mutasynthetic generation of cahuitamycins D and E (**4** and **5**) and their complete structures. LC-ESI-MS chromatograms of **4** (**a**) and **5** (**b**) obtained from a culture of the *cahI* deletion mutant DHS334 supplemented with 100 μM of 5-methylsalicylic acid.

The biosynthetic process for generating **3** revealed the possible tolerance of CahJ towards structurally related salicylic acid substrates, and a promising avenue for producing new cahuitamycin analogues by mutasynthesis^52^. In this approach, we provided the DHS334 Δ*cahI* strain with a series of substituted benzoic acid substrates (Supplementary Fig. 40), and sequentially assessed their incorporation to make new analogues (Fig. 4). Of the series of unnatural starter units tested, feeding of methyl-hydroxybenzoic acid substrates showed some level of incorporation. However, only assimilation of 5-methylsalicylic acid into the Δ*cahI* pathway provided isolable quantities of the new analogue, cahuitamycin D (**4**; Fig. 4a; Supplementary Fig. 40).

Cahuitamycin D (**4**) was isolated by reverse-phase HPLC (RP-HPLC) from crude extract of Δ*cahI* mutant. The HRESIMS [M+H]^+^ ion peak at *m/z* 650.2706 provided similar molecular formula as of **3**, C_28_H_39_N_7_O_11_. Extensive 1D and 2D NMR data were acquired for **4**, which indicated the expected structural similarity with **3**, as per the mutasynthesis hypothesis (Fig. 4a; Supplementary Table 2). The structure showed a similar carbon backbone with a clear difference at the phenyl ring system compared with **3**. Cahuitamycin D (**4**) shows the presence of a singlet at δ_H_ 7.78 (H-23) with HMBC correlation to δ_c_ 20.3 (C-25) and δ_c_ 129.4 (C-24), suggesting methylation at C-24 consistent with the hypothesized incorporation of 5-methylsalicylic acid to the mutant strain DHS334 of *S. gandocaensis*.

Furthermore, during isolation of **4**, we observed another HPLC peak eluting with this natural product (Fig. 4b). Interestingly, the HRESIMS [M+H]^+^ ion peak at *m/z* 668.2830 suggested the same backbone with likely additional hydration. The new molecule cahuitamycin E (**5**) was shown by 1D and 2D NMR analysis to share structural similarity on much of the carbon backbone compared with **4** (Supplementary Table 2). The only plausible difference could be traced to the C-5 position where Pip moiety in **4** was substituted with L-*N*-OH-Orn in **5**, suggested based on the COSY relay observed from δ_H_ 4.33 (H-5) to δ_H_ 3.45 (H-8) along with absence of any significant HMBC correlation from H-8 to C-9 (δ_C_ 172.2) (Fig. 4b). The observation was compelling as it indicates that either the cyclization to form Pip moiety is occurring after the insertion of L-*N*-OH-Orn in **5** or, alternatively, the domain is capable of accepting both moieties separately. In addition, the study also indicated that L-glutamine might not be a substrate for the CahC A domain during biosynthesis of cahuitamycins.

The absolute stereochemical configuration of the cahuitamycins D and E (**4** and **5**; Fig. 4) was extrapolated to be the same as cahuitamycins A–C (**1**–**3**) based on very similar chemical shifts observed for all nuclei obtained through detailed 1D and 2D NMR analysis (Supplementary Table 2).

### Biological activity associated with cahuitamycins A–E

**Figure 5:**
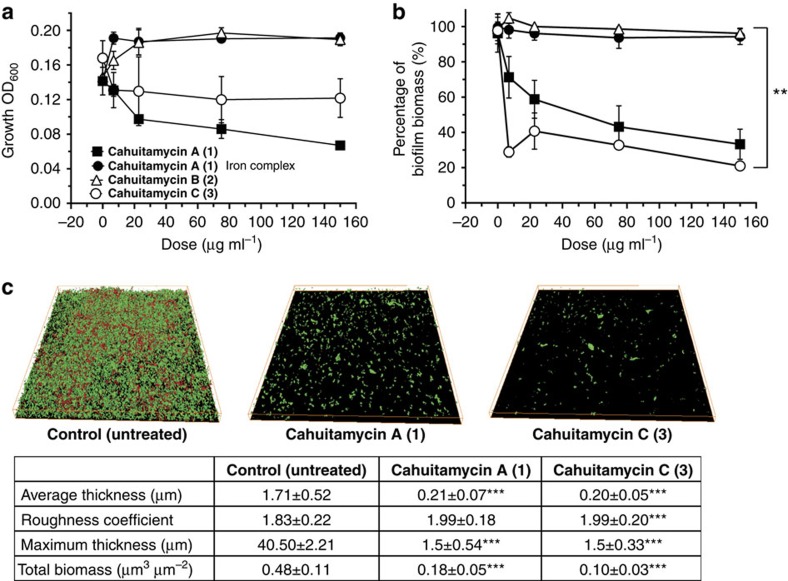
Biological activity of cahuitamycins A–C (**1**–**3**). (**a**) Growth of *A. baumannii* ATCC 17978 and (**b**) inhibition of biofilm in the presence of cahuitamycins A–C (**1**–**3**) and iron complex of **1** compared with control. Results are the average of three replicates±s.d. Student's *t*-test was used for statistical analysis, ** indicates *P*<0.01. (**c**) 3D image of *A. baumannii* ATCC 17978 biofilm after 22 h of incubation. Control was untreated, and **1** and **3** were treated at 240 nM. Images were acquired using a confocal microscope with a × 60 objective lens. Results are the average of five replicates. Student's *t*-test was used for statistical analysis, *** indicates *P*<0.001 compared with control.

Cahuitamycins A–C (**1**–**3**) were next tested for their ability to inhibit biofilm formation of *A. baumannii* using a high-resolution optical secondary assay system. The primary high-throughput static biofilm assay leading to identification of **1**–**3** was conducted using crystal violet staining followed by optical density measurements (Fig. 5a,b). The result from this assay showed that **1** was able to inhibit, while **2** had no effects on bacterial growth or biofilm formation (Fig. 5a,b). Interestingly, **3** possessed the highest potency with half-maximal inhibitory concentration (IC_50_) values of 14.5 μM while having a limited effect on growth of *A. baumannii.* In addition, a confocal laser scanning microscope imaging analysis of biofilm formation and biomass was performed (Methods). In this assay, the biofilm was developed on a 12-well glass plate with solvent (no compound) as a negative control and only 240 nM of **1** and **3** as treated. After staining with fluorescent dye SYTO9 and propidium iodide, the biofilm structure was observed (Fig. 5c). The reconstructed three-dimensional (3D) biofilm images obtained showed a much thinner biofilm and also significantly less total biomass for the glass plates treated with **1** and **3** compared with control (Fig. 5c). Furthermore, the glass plate treated with **3** showed a much lower level of total biomass compared with **1** at the same concentration (0.10 μm^3^ versus 0.18 μm^3^), which confirmed **3** as the more potent biofilm inhibitor (Fig. 5c).This finding indicates that cahuitamycins prevent biofilm formation through a specific, yet currently undefined biochemical target with negligible impact on cell growth (Fig. 5a,b). Furthermore, cahuitamycin C (**3**) was tested against the growth of *A. baumannii* separately (Supplementary Fig. 41), and revealed an IC_50_ value of ⩾1.0 mM (Supplementary Fig. 42). In addition, when **1** (the most abundant molecule) was tested in iron-complexed form (owing to its moderate iron chelation affinity), it showed minimal impact on both growth and biofilm formation of the pathogen (Fig. 5a,b). To probe iron chelation as a possible mode of action for the cahuitamycins, a known siderophore desferrioxamine was used as control, which showed no activity, suggesting that iron chelation (or depletion) may not be the basis for cahuitamycin activity (Supplementary Figs 38 and 44). However, loss of inhibition of biofilm formation over time could be attributed to the metal-complexed cahuitamycins (Supplementary Figs 38 and 44), as the test media is replete with iron and **1** in complex with Fe^III^ has not displayed comparable inhibitory activity (Fig. 5a). This observation is consistent with previous studies, which have reported that clinical isolates of *A. baumannii* are able to grow in the presence of high concentrations of different iron chelators due to its ability to express a high-affinity extracellular siderophore, acinetobactin^53^,^54^. In addition, the structural differences between cahuitamycins with previously reported amychelin^30^, results in a significant change in iron affinities, which can be further explored for biofilm inhibitory properties in future studies.

Next, we sought to probe the specific stage and extent of inhibition during biofilm formation employing a secondary flow cell assay (Supplementary Fig. 44; Methods). This analysis revealed almost the same number of cells initially attached to the surface before starting treatment. After 6 h of inhibitor supplementation, the flow cells treated with **1** showed only a few microcolonies of the pathogen, while the control (no treatment) and desferrioxamine showed formation of a robust biofilm. After 24 h of supplementation, the control and desferrioxamine biofilm became thicker with the flow cells exposed to **1** showing larger microcolonies and initiation of biofilm formation by *A. baumannii*.

**Figure 6:**
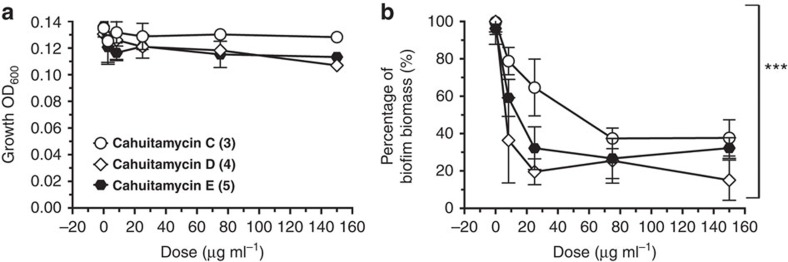
Biological activity of cahuitamycins C–E (**3**–**5**). (**a**) Growth of *A. baumannii* and (**b**) inhibition of biofilm in the presence of cahuitamycins C–E (**3**–**5**). Results are the average of three replicates±s.d. Student's *t*-test was used for statistical analysis. ***P<0.001 compared with control (no addition of compounds).

As described above, the mutasynthetic study on *S. gandocaensis* strain DHS334 led to the isolation of two additional analogues cahuitamycins D–E (**4**–**5**). The new molecules were also subjected to static biofilm assays and the result demonstrated that **4** displayed almost twice the potency (IC_50_=8.4 μM) compared with **3,** which we earlier considered to be the most active molecule (Fig. 6; Supplementary Figs 41–43). Furthermore, cahuitamycin E (**5**) also displayed significant activity with an IC_50_ of 10.5 μM (Fig. 6; Supplementary Figs 41–43). In addition, compounds **3** and **4** were also tested for disruption of established *A. baumannii* biofilm revealing low activity with an IC_50_ of 692 and 535 μM, respectively (Supplementary Fig. 45; Methods), establishing their better efficacy as biofilm formation inhibitors. Although prophylactic antibiotic administration preceding surgery is routinely successful in reducing infection rates, it has little or no protective effects in surgical procedures involving implants or prostheses^55^. Therefore, catheters and implants coated with biofilm-inhibiting antimicrobial agents are urgently needed as an effective, precision therapeutic option. Our current data indicate that a key pharmacophore of the cahuitamycins is likely the 2-hydroxybenzoyl-oxazoline group where relatively minor modifications can result in an increase (as in case of **3**–**5**) or decrease (as for **2**) of anti-biofilm activity, with the potential for further structure activity relationship studies using synthetic chemistry or Cah pathway engineering.

The application of the streptomycin resistance-mediated screening of the *Streptomyces* strain identified from HTS against *A. baumannii* has yielded a novel structural class of biofilm inhibitors derived from a marine microbial NPE library. Cahuitamycins A–E (**1**–**5**) inhibit the ability of *A. baumannii* to generate a biofilm, which is involved in the emergence of a widespread antibiotic resistance phenotype^6^,^56^. The cahuitamycins are most active in apo form, with chelation of Fe^III^ over time resulting in attenuated biological activity. The cahuitamycins along with allied chemical entities with distinct biological activity provides a primary foundation for future medicinal chemistry and synthetic exploration towards the development of an efficacious drug to prevent or limit biofilm formation.

We have also demonstrated that production of cahuitamycins A–C is specified by a convergent pathway specified by the *cah* biosynthetic gene cluster comprised of two unlinked genetic loci. The inherently flexible starter unit adenylating enzyme CahJ was exploited to produce two additional analogues in the engineered Δ*cahI* strain DHS334. The new cahuitamycin D analogue displays twofold-enhanced biofilm inhibitory activity compared with the cahuitamycin C natural product. These results establish a unique opportunity for developing and discovering new antibiotics from genetically engineered strains bearing inherent flexibility in pathway initiation processes. More importantly, given the simultaneous decline in antibiotic drug discovery and increased incidence of multidrug resistant bacteria, the cahuitamycins may represent a propitious starting point for discovery and development of new therapeutics against dangerous human pathogens involving biofilm formation.

## Methods

### General experimental procedures

Optical rotation measurements were obtained on a Perkin-Elmer 241 Polarimeter calibrated using a Rudolph Quartz Control Plate Calibration Standard at sodium D line (at +11.502°). Ultraviolet spectra were obtained on a UV-visible Molecular Devices SpectraMax M5 spectrophotometer using 1-ml cuvettes with 1.0 cm path lengths at room temperature in solvent methanol (MeOH). Spectrophotometric assays were performed on Molecular Devices SpectraMax M5 384 variable wavelength spectrometer. All NMR spectra were acquired on a Varian INOVA 600 MHz and a Varian INOVA 700 MHz spectrometer at the NMR Facility, Department of Chemistry, University of Michigan. HRESIMS spectra were measured at the University of Michigan core facility in the Department of Chemistry using an Agilent 6520 Q-TOF mass spectrometer equipped with an Agilent 1290 HPLC system. RP-HPLC was performed using Econosil C18 10 μm 22 × 250-mm column and Agilent ZORBAX RX-C8 5 μm 9.4 × 250-mm column and a solvent system of MeCN and H_2_O. The LC-MS analysis of HPLC fractions was performed on a Shimadzu 2010 EV APCI spectrometer.

### Biological material

*S. gandocaensis* (Strain # 12620-H2) was isolated from marine sediments collected from Punta Mona Island (protected area: −82^o^ 37′ 1.50′′, 09^o^ 37′ 56.2′′ RVS Gandoca Manzanillo with collection permit R-CM-INBio-30-2007), Costa Rica, on 5 June 2007. The procedure for the isolation of actinomycetes from these samples was previously described by Magarvey *et al*.^16^ Maintenance and propagation of cultures were performed using standard media and protocols where 500 mg of wet sediment was diluted in 10 ml of sterile water and vortexed for 10 min. Then, 1 ml of this suspension was applied directly to the top of the discontinuous sucrose gradient and centrifuged for 30 min at 300*g*. A volume of 500 μl of the 20, 30 and 40% layers was then plated to HVA agar supplemented with 10 μg ml^−1^ chlortetracycline, 25 μg ml^−1^ cyclohexamide and 25 μg ml^−1^ of nalidixic acid. The plates were then incubated at 28 °C for 1 month. The colony was picked off the plate and streaked onto ISP2 agar until pure. Seed cultures were grown in 17-ml dual-position cap tubes containing 2 ml of ISP2 and grown for 4 days on a rotary shaker at 200 r.p.m. The seed culture was then poured into a 250-ml baffled flask containing 100 ml of ISP2 and grown for 18 days on a rotary shaker at 200 r.p.m. The culture was centrifuged at 4,000 r.p.m. for 10 min to remove the cells, and 2 g of XAD16 resin (Sigma-Aldrich, St Louis, Mo) contained within a polypropylene mesh bag was added to the broth and incubated overnight on the rotary shaker. The resin bag was removed and placed into 10 ml of MeOH, followed by 10 ml of acetone and 10 ml of ethyl acetate (EtOAc). Each of the three fractions was dried *in vacuo* and reconstituted to a final concentration of 15 mg ml^−1^ in DMSO.

### Culture maintenance and fermentation

Seed cultures of 100 ml (5 × ) of ISP2 media (1% malt extract, 0.4% yeast extract, 0.4% dextrose and 3% NaCl) were inoculated with a loop-full of vegetative cells from an oatmeal plate (6% oat meal, 1.25% agar and 3% NaCl) culture of *S. gandacoensis* and incubated with shaking (200 r.p.m.) at 28 °C for 5 days. A 25 ml portion of the seed cultures were transferred to a 2.8-l Fernbach flask containing 1.5 l of the ISP2 medium, and the 39 l fermentation was carried out on a rotary shaker (200 r.p.m.) at 28 °C for 18 days. After 14–18 days of growth, the cultures were collected by centrifugation. The resulting cell-free broth was subjected to solid phase extraction using 15 g of Amberlite XAD-16. The resin was then separated by filtration and subjected to organic extraction using MeOH:EtOAc (1:1).

### Natural product extract library

The NPE library at the University of Michigan Center for Chemical Genomics contained ∼20,000 extracts. Each extract in the library is derived from marine samples collected from all over the world, including Costa Rica, Panama and Papua New Guinea. Some of these samples are from isolated microbes (*n*=19,055), while others were derived from field-collected biomass samples (‘macrosamples,' *n*=800). Previous work describes in detail how these extracts are prepared for the library^16^.

### PCR amplification, cloning and sequencing of 16S rDNA

Genomic DNA of *S. gandocaensis* was isolated using the Wizard Genomic DNA Purification Kit according to the manufacturer's instruction. The 16S ribosomal DNA (16S rDNA) gene was amplified by PCR with the universal primers FC27 and RC1492 (Supplementary Table 6)^57^ using the genomic DNA as a template. The PCR fragments were cloned into pGEM-T Easy vector (Promega) and the resulting plasmid containing 16S rDNA of *S. gandocaensis* was sequenced using T7 and SP6 primers.

### Phylogenetic analysis of 16S rDNA of *S. gandocaensis*

Phylogenetic analyses were conducted using GENEIOUS R6 that is available from http://www.geneious.com/ (ref. 58). The evolutionary history was inferred using the Neighbour-Joining method. The bootstrap consensus tree inferred from 500 replicates was taken to represent the evolutionary history of the taxa analysed (Supplementary Fig. 6).

### Generation of ribosome-engineered mutants of *S. gandocaensis*

Spores (10^8^–10^9^) of wild-type *S. gandocaensis* were spread on R2YE agar containing various concentrations of streptomycin, followed by 15 days incubation at 28 °C to allow the development of streptomycin-resistant colonies. Eighty resistant colonies (Supplementary Fig. 4) were obtained from the plate containing a high level (10, 50 and 100 μg ml^−1^) of streptomycin and grew in ISP2 media with streptomycin for 14 days at 28 °C. This step was repeated four times to obtain spontaneous streptomycin-resistant mutants. The ability of selected mutants to produce cahuitamycin was tested by LC-MS using Agilent extend C18 5 μm 2.1 × 150-mm column on gradient condition 30–85% acetonitrile (w/0.1% formic acid) in H_2_O (w/0.1% formic acid) over 15 min with additional 5 min of equilibration time.

### Amplification and sequencing of *rpsL*

Nine strains, including the parent strain and eight high-yielding recombinants, were selected to determine mutations in the *rpsL* gene in this study. The *rpsL* gene was amplified by PCR with primers SR185 and SR186 (Supplementary Table 6), using genomic DNA as a template and cloned into pGEM-T easy vector (Promega) and sequenced. Mutations in the wild-type *rpsL* gene were determined by DNA sequencing^59^ (Supplementary Fig. 5; Supplementary Table 3).

### Isolation and purification of cahuitamycins

The organic extracts obtained from the engineered strains were concentrated under vacuum to afford the crude extracts (∼350 mg) obtained from 100-ml culture. The crude extract was assayed in the developed *in vitro* crystal violet at 10 and 1.0 p.p.m. The bioactive extract was then further purified by RP-HPLC on a gradient of 10–75% ACN and was followed by ultraviolet–visible photodiode array detection at 215 nm to yield semi-pure compounds 1 (6.7 mg), 2 (2.4 mg) and 3 (2.9 mg). Compounds were again subjected to repurification over RP-HPLC on isocratic condition of 35% MeOH (0.1% FA) using C-8 column to get compounds 1 (5.1 mg), 2 (1.1 mg) and 3 (1.6 mg).

*Cahuitamycin A (**1**).* Bone white, amorphous powder; ultraviolet (ACN:H_2_O) *λ*_max_ 203, 245, 249, 255 and 304 nm; ^1^H and ^13^C NMR, see Supplementary Table 1; HRESIMS *m/z* 636.2679 [M+H]^+^ (calculated for C_27_H_38_N_7_O_11_, 636.2629).

*Cahuitamycin B (**2**)*. Bone white, amorphous powder; ultraviolet (ACN:H_2_O) *λ*_max_ 203, 240, 249, 255 and 304 nm; ^1^H and ^13^C NMR, see Supplementary Table 1; HRESIMS *m/z* 654.2759 [M+H]^+^ (calculated for C_28_H_40_N_7_O_11_, 654.2735).

*Cahuitamycin C (**3**)*. Bone white, amorphous powder; ultraviolet (ACN:H_2_O) *λ*_max_ 200, 245, 249, 255 and 304 nm; ^1^H and ^13^C NMR, see Supplementary Table 1; HRESIMS *m/z* 650.2804 [M+H]^+^ (calculated for C_27_H_38_N_7_O_11_, 650.2786).

### Generation of *cahI* deletion mutant

The *cahI* gene was inactivated by an insertional inactivation via double-crossover homologous recombination (Supplementary Fig. 7A). A knock-out plasmid pSRP50 based on pKC1139 (ref. 60) was constructed by amplifying kanamycin resistance gene as a selection marker from plasmid pYJ276 (ref. 61), and left-and-right-flanking regions of the *cahI* gene using the genomic DNA of *S. gandocaensis* as a template. The primer pairs SR144-SR145, SR146-SR147 and SR148-SR149 (Supplementary Table 6) were designed for the amplification of left-and-right-flaking fragments of *cahI* gene, and selection marker, respectively. DNA assembly was performed using Gibson assembly^62^ master mix (New England Biolabs) according to the manufacturer's instructions. The plasmid pSRP50 was passaged through methylation-deficient *E. coli* ET12567 and then introduced into the *S. gandocaensis* by protoplasts-based transformation^63^. The target region of *cahI* gene was then disrupted by an insertional inactivation via double-crossover homologous recombination. The desired mutant Δ*cahI* was selected by its kanamycin-resistant and apramycin-sensitive phenotype (Supplementary Fig. 7b) and verified by PCR (Supplementary Fig. 7c) using the primer pair SR233–SR234 (Supplementary Table 6). The resulting *cahI* deletion mutant of *S. gandocaensis* was designated as DHS334.

### Chemical complementation of mutant DHS334 and mutasynthesis

Mutant strain of DHS334 was first precultured in 3 ml R2YE liquid medium for 15 days at 28 °C and then 3 ml of the seed culture was used to inoculate 100 ml of the same medium, followed by cultivation for 15 days at 28 °C. Salicylic acid and a series of substituted benzoic acid substrates, 2-hydroxybenzoic acid, 2,3-dihydroxybenzoic acid, 2,4-dihydroxybenzoic acid, 2-fluorobenzoic acid, 2-hydoxy-5-methylbenzoic acid (5-methylsalicylic acid) and 2-hydoxy-6-methylbenzoic acid (6-methylsalicylic acid) were added every alternate day to separate 100-ml cultures of DHS334 at a final concentration of 500 μM for 15 days. The products were first extracted using Amberlite XAD-16. The resin was separated and subjected to organic extraction using MeOH:EtOAc (1:1) for LC-MS analysis as described above.

The substrate-fed crude extract was then further purified by RP-HPLC on a gradient of 10–75% ACN and was followed by ultraviolet–visible photodiode array detection at 215 nm to yield semi-pure compounds **4** (2.4 mg) and **5** (2.6 mg). Compounds were again subjected to repurification over RP-HPLC on isocratic condition of 35% MeOH (0.1% FA) using C-8 column to get compounds **4** (1.1 mg) and **5** (1.3 mg).

Cahuitamycin D (**4**). Amorphous powder; ultraviolet (ACN:H_2_O) *λ*_max_ 203, 240, 249, 255 and 304 nm; ^1^H and ^13^C NMR, see Supplementary Table 2; HRESIMS *m/z* 650.2706 [M+H]^+^ (calculated for C_28_H_40_N_7_O_11_, 650.2786).

Cahuitamycin E (**5**). Amorphous powder; ultraviolet (ACN:H_2_O) *λ*_max_ 200, 245, 249, 255 and 304 nm; ^1^H and ^13^C NMR, see Supplementary Table 2; HRESIMS *m/z* 668.2830 [M+H]^+^ (calculated for C_28_H_42_N_7_O_12_, 668.2891).

### Determination of *pFe* ^III^ for cahuitamycins A–C

Determination of pFe^III^ for cahuitamycins was carried out as reported by Abergel *et al*. essentially without modifications^64^. Briefly, purified Fe-cahuitamycins, prepared as described above, was dissolved in HEPES buffer (10 mM HEPES and 0.1 M KCl, pH 7.4) and five different concentration ranges of ETDA were added from EDTA stock solutions also prepared in HEPES buffer. Each reaction consisted of a total volume of 0.25 ml, a final concentration of 0.1 mM Fe-cahuitamycins and a range of 5–8,000-fold EDTA (relative to Fe**-**cahuitamycins) in HEPES buffer. The reaction was allowed to equilibrate at room temperature for at least 24 h, and ultraviolet–visible spectra were subsequently recorded. The composite spectra contain contributions from both Fe-EDTA and Fe-cahuitamycins. The *ɛ* of both species as a function of *λ* were determined in HEPES buffer (see Supplementary Fig. 38 for Fe-cahuitamycins) and used to deconvolute the spectra. The contribution of Fe-cahuitamycins was subtracted from the composite spectra using the 435 nm absorption band, and the *ɛ* of both Fe-cahuitamycins and Fe-EDTA were used to quantify the proportion of each species in solution. Concentrations of apo-EDTA and apo-cahuitamycins were calculated by subtracting [Fe-EDTA] (or [Fe-cahuitamycins]) from total initial EDTA (or Fe-cahuitamycins). The log [EDTA]/[cahuitamycins] was plotted against log [Fe-EDTA]/[Fe-cahuitamycins] and the data were fit to equation (1), which has been derived by Abergel *et al*.^64^. All the data sets are shown in Supplementary Fig. 38, yielding a pFe^III^ as reported in original article.


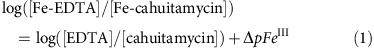


### Assay development and high-throughput screening assay

A static biofilm assay was developed from a previously reported method^19^. The biofilm assay measures the adhesion of bacteria to the surface of polystyrene 384-well microtiter plates. Adherent cells are detected by staining with crystal violet and subsequent washing to remove nonadherent planktonic cells. Inhibitors of biofilm formation prevent the bacteria from adhering to the surface of the microtiter plate and reduce the amount of crystal violet retained after washing. Assay quantification was performed by OD_600_ absorbance measurement of the crystal-violet-stained biofilm. The assay was optimized for plate surface treatment, time, temperature, media, media concentration, crystal violet concentration, wash method, inhibitor sensitivity and culture inoculum preparation.

The *A. baumannii* test strain (ATCC 17978) was maintained as a frozen stock at −80 °C in 20% glycerol. A volume of 5 ml of Mueller Hinton II cation adjusted media (Becton Dickinson, catalogue no. 212322) was inoculated and grown at 37 °C, 180 r.p.m. shaking for 4–6 h. The culture was then diluted 1:50 and incubated an additional 2 h. The resulting cells were washed four times by centrifugation and resuspension of the pellet in media was performed to remove any metabolites or cell signalling factors. The assay inoculum was prepared by diluting the cells to 0.008 OD_600_ in 10% Mueller Hinton II cation adjusted media (diluted in 18 Ohm deionized water). The assay plates (Corning, catalogue no. 3,680; 384-well non-treated polystyrene) were prepared by dispensing 20 μl of 10% Mueller Hinton II cation adjusted media into columns 1–22 using a Multidrop Combi dispenser (Thermo Fisher). The NPE samples were added as 0.2 μl of 15 mg ml^−1^ stocks in DMSO using a Biomek FX high-density pintool (Beckman Coulter). Column 1 and 2 contained DMSO without NPEs and served as the negative control. The positive control was added to column 23 and 24 as 20 μl of 40 μM Baicalein (Sigma-Aldrich, catalogue no. 11712). The bacterial cells were added to the entire plate in 20 μl of the prepared inoculum using a Multidrop Combi dispenser (Thermo Fisher). The resulting assay contained 40 μl of 10% Mueller Hinton II cation adjusted media with 0.004 OD_600_ bacterial cells, 0.5% DMSO and 75 μg ml^−1^ NPE. The assay plates were incubated at 30 °C for 20 h, stationary, in a humidified incubator. The biofilm was stained by adding 10 μl of filtered 1% crystal violet and incubating for 30 min at room temperature. Excess crystal violet and nonadherent planktonic cells were removed by washing three times with 150 μl of PBS using an ELX405 plate washer (Bio-tek). The wash program used a low velocity dispense rate and an aspiration height of 3.8 mm; ∼10 μl of PBS remained in the well after washing. The Crystal-Violet-stained biofilm was solubilized by the addition of 50 μl of 100% ethanol and allowed to develop overnight. Quantification was performed by measuring the OD_600_ absorbance using a Pherastar plate reader (BMG).

### 3D imaging of biofilm development

For visualizing bacterial biofilm formation, the same method as described previously was used for biofilm formation but in a glass-bottom 96-well plate (12–556–38, Fisher Scientific International Inc., Franklin, MA)^65^. After 20 h of incubation at 30 °C, the supernatant was removed, and the well surface was rinsed briefly with 1 × PBS buffer three times. PBS buffer (100 μl) containing 1 μM of SYTO 9 and 10 μM of propidium iodide was added into the well. The plate was incubated for 15 min in dark. Fluorescent images were acquired with an Olympus Fluoview FV1000 confocal microscope (Olympus, Markham, Ontario) with Melles Griot Laser supply and detectors and filter sets for monitoring SYTO 9 and propidium iodide fluorescence. Images were obtained using an oil immersion × 60 objective lens. 3D images were reconstructed using the Amira software package (Amira, San Diego, CA) from a stack of sectional images of biofilm samples. Biofilm biomass and per cent coverage were calculated based on those images using software COMSTAT^65^.

### Micrograph of biofilm in flow cells

Biofilms were formed by *A. baumannii* ATCC 17978 in flow chambers at room temperature. Images were taken at 1, 6 and 24 h (using a × 60 lens) after inoculation. A flow cell biofilm was achieved by using a flow cell chamber (ACCFL0001, Life Science Incorporated, Greensboro, NC). Briefly, overnight cultures of *A. baumannii* ATCC 17978 grown in MHII broth at 37 °C were diluted 100-fold with fresh MHII broth. A volume of 1 ml of dilution was injected into a flow cell chamber and allowed to settle 1 h for attachment of bacteria, followed by initiation media flow at a flow rate of 4 ml^−1^ h^−1^. Flow media was 10% MHII broth (control), 10% MHII broth with 7.5 μg ml^−1^ of desferoxamine or 10% MHII broth with 7.5 μg^−1^ ml of cahuitamycin A. Micrographs of the biofilm were acquired at 1, 6 and 24 h after the start of flow media using an Olympus IX70 microscope with a × 60 lens. Flow was still running while acquiring the micrograph.

### Minimum inhibitory concentration test

Cultures of *A. baumannii* ATCC 17978 grown overnight were diluted into fresh 10% Mueller Hinton II broth to get 10^5^ colony-forming unit per ml by determining optical density at OD_600_. A volume of 100 μl of diluted cultures were incubated in individual wells of 96-well plate containing 1% of DMSO and various concentrations of cahuitamycins C (**3**) and D (**4**). The plate was incubated at 30 °C with shaking at 150 r.p.m. The optical density of each well was monitored every 1 up to 24 h using a microtiter plate reader (Synergy HT, Bio-Tek, Winooski, VT). The number of viable bacterial cells was determined by counting colony-forming unit.

### Biofilm dispersal assay

Biofilm dispersal assay was performed as previously reported^66^. Briefly, *A. baumannii* ATCC 17978 biofilm was developed as described in minimum inhibitory concentration test. After 16 h incubation at 30 °C, the suspension in each well was removed by pipetting gently. A volume of 100 μl of 1 × PBS buffer containing cahuitamycins C (**3**) and D (**4**) at various concentrations was placed into each well. The plate was incubated at 30 °C for 12 h to allow cahuitamycins to disassemble pre-formed biofilms. Then, the suspension was removed by pipetting. The remaining attached biofilm was quantified by crystal violet assay.

## Additional information

**Accession codes:** The 16S rDNA sequence for *S. gandocaensis* (strain #12620-H2) has been deposited in the GenBank Nucleotide database with accession code KR303715. The DNA sequences for the two cahuitamycin genetic loci have been deposited in the GenBank Nucleotide database with accession codes KU363800 and KU363801. A description and analysis of the complete *S. gandocaensis* genome will be described elsewhere.

**How to cite this article:** Park, S. R. *et al*. Discovery of cahuitamycins as biofilm inhibitors derived from a convergent biosynthetic pathway. *Nat. Commun.* 7:10710 doi: 10.1038/ncomms10710 (2016).

## Supplementary Material

Supplementary Information Supplementary Figures 1-45, Supplementary Tables 1-7 and Supplementary References
