# Supplementary material for: Discovery of cahuitamycins as biofilm inhibitors derived from a convergent biosynthetic pathway
Source: Nat Commun. 2016 Feb 16;7:10710. doi: 10.1038/ncomms10710 (PMC4757757; doi:10.1038/ncomms10710)
Supplement: Supplementary Information — Supplementary Figures 1-45, Supplementary Tables 1-7 and Supplementary References [file ncomms10710-s1.pdf]

Supplementary Information

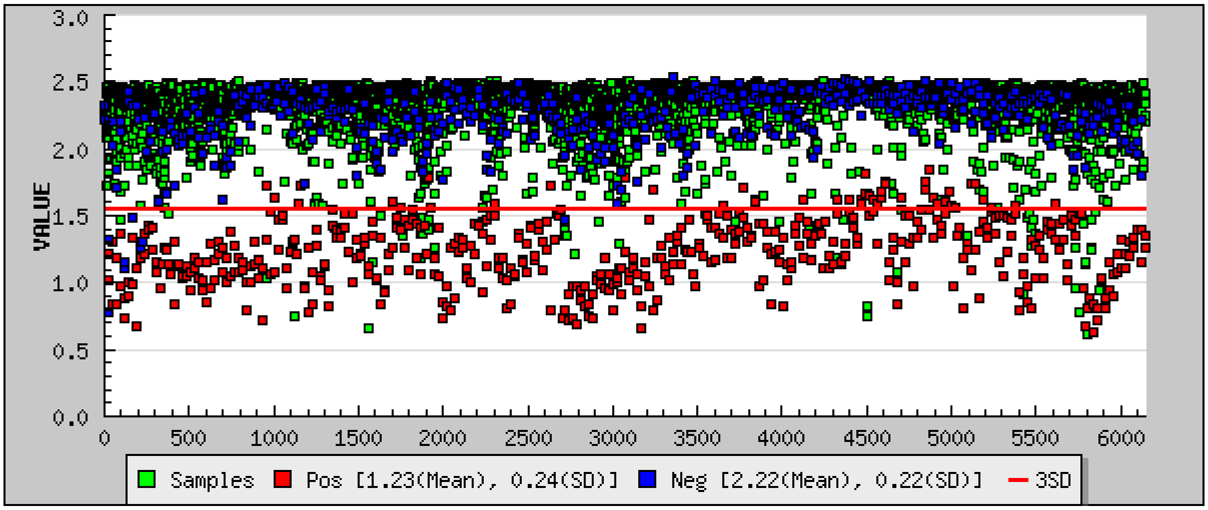

Supplementary Figure 1. High throughput screening assay view of sixteen -384 well plates containing NPEs. Each dot represents a single well reaction. Red dots represent control inhibitor, baicalein; blue dots represent DMSO control; and green dots represents test extracts wells; red line represents the value of three standard deviations of the DMSO control. (X-axis is the compounds tested, Y-axis is the absorbance value of crystal violet assay at 600 nm)

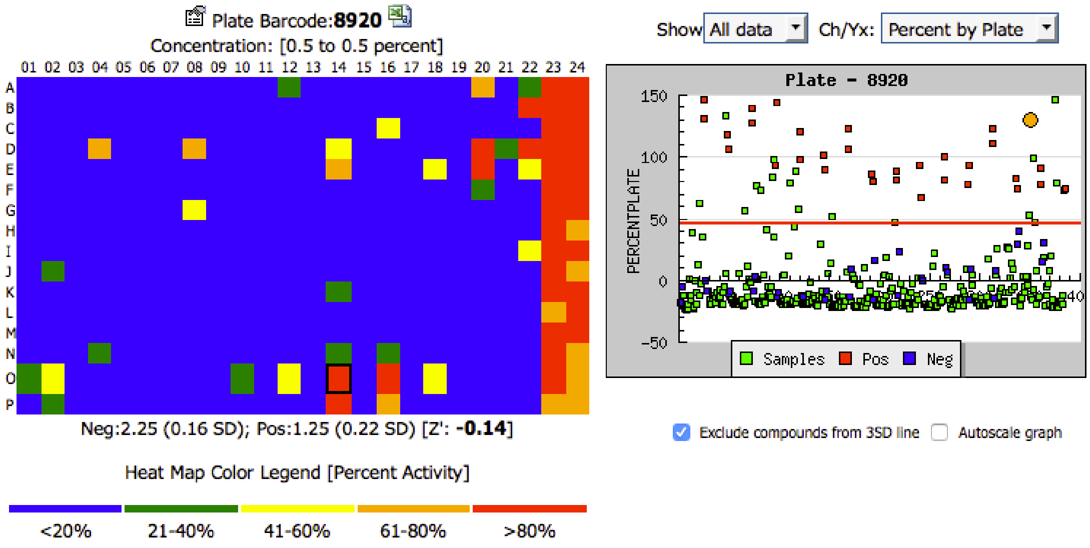

Supplementary Figure 2. High throughput screening plate view of the plate containing selected active strain

- 384-well plate layout with biofilm in each wells on left
- *S. gandocaensis* shown by orange dot on Plate 8920 on right (X-axis is the extracts tested, Y-axis is relative capacity of biofilm prevention (normalized by control baicalein).

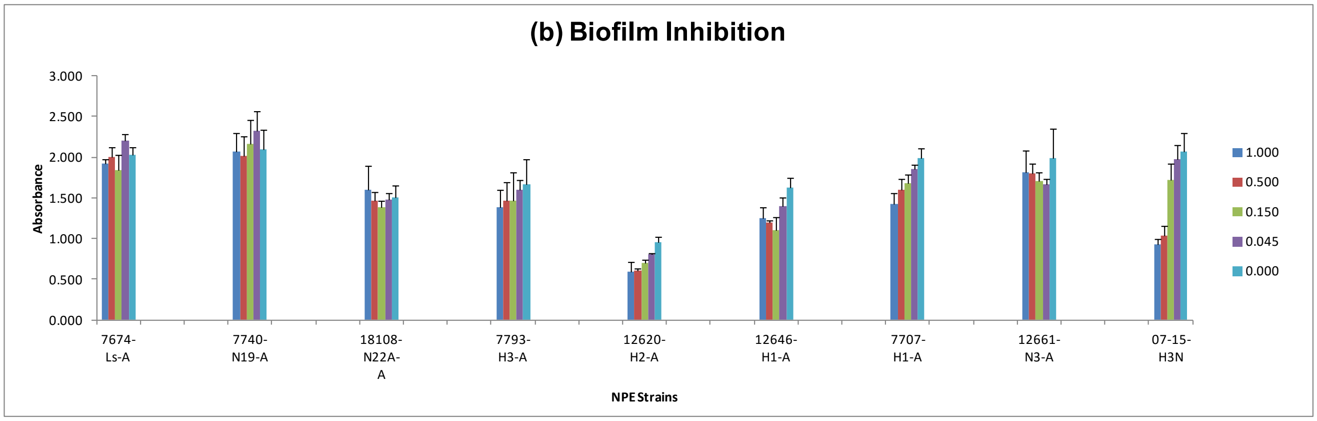

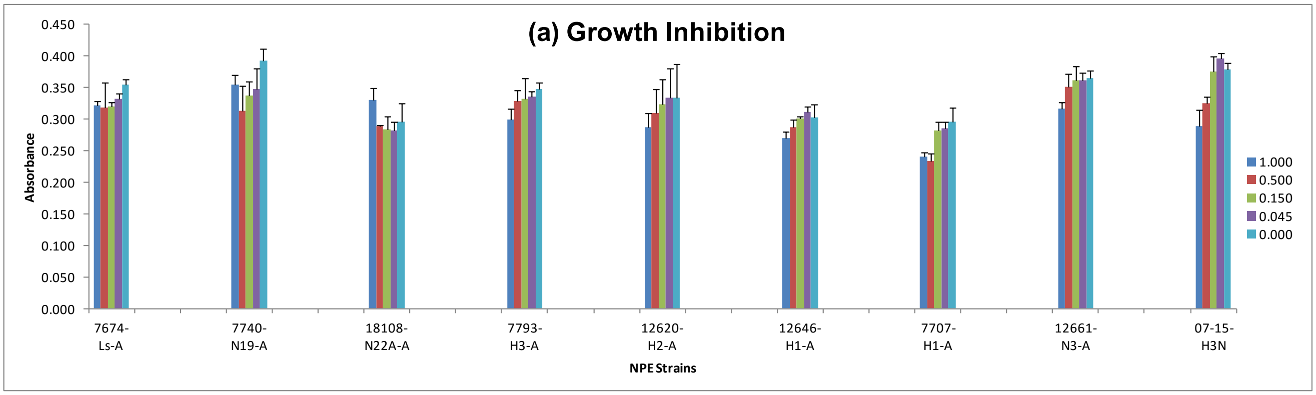

Supplementary Figure 3. Secondary confirmation assay of the selected top hits for further scale up culture. Biofilms were developed in 96-well plate and biomass was obtained through crystal violet assay at OD600. Extracts concentration is in µg/ml (legend). X-axis is the compounds tested, Y-axis is the absorbance value of crystal violet assay at 600 nm (All the experiments were conducted in triplicates). Results are the average of three replicates ± SD. Student's *t* test was used for statistical analysis, ** indicates $P < 0.01$.

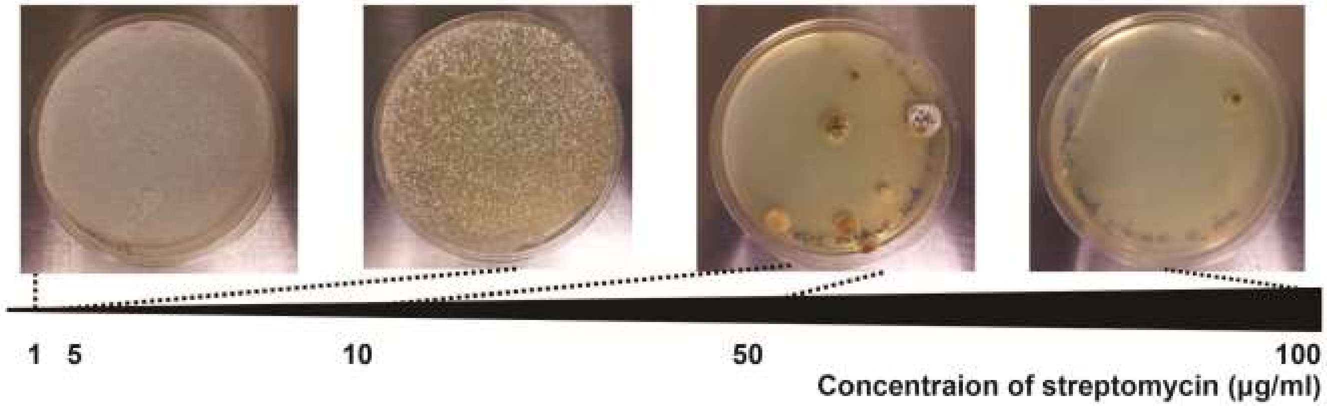

Supplementary Figure 4. Selection of streptomycin-resistant colonies of *S. gandocaensis*

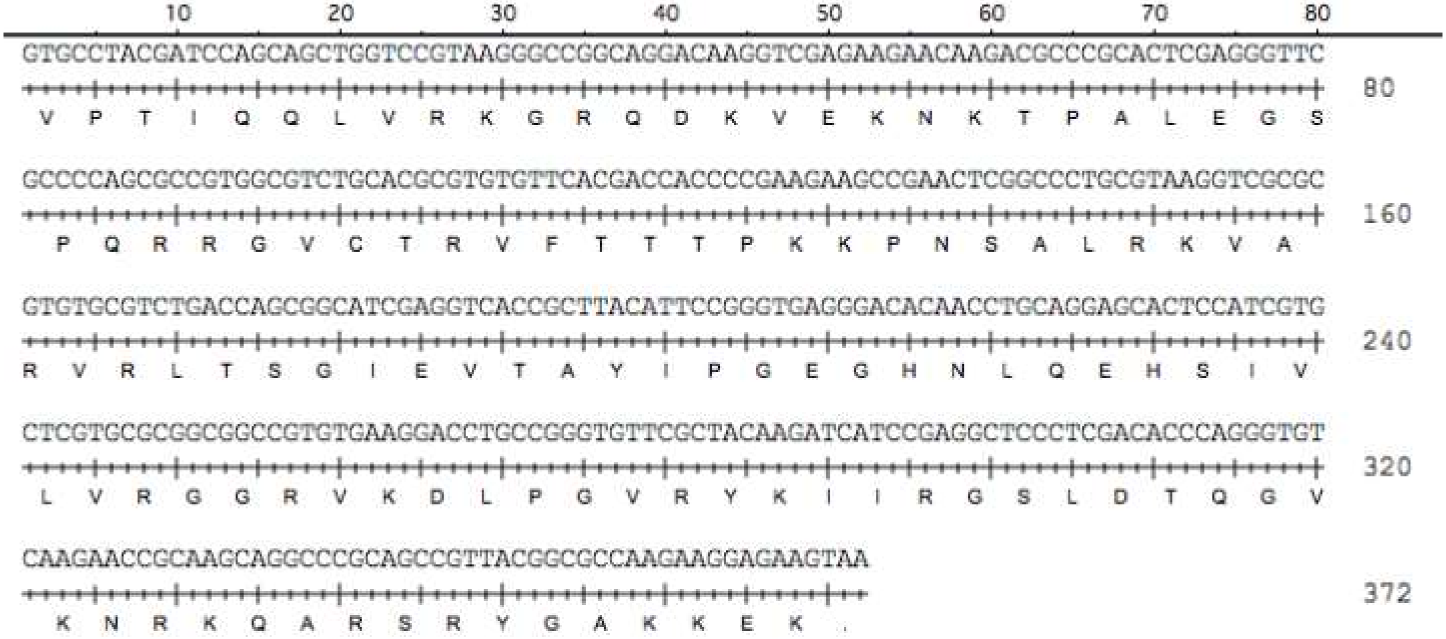

Supplementary Figure 5: Sequence of putative *rpsL* gene from parent strain of *S. gandocaensis*

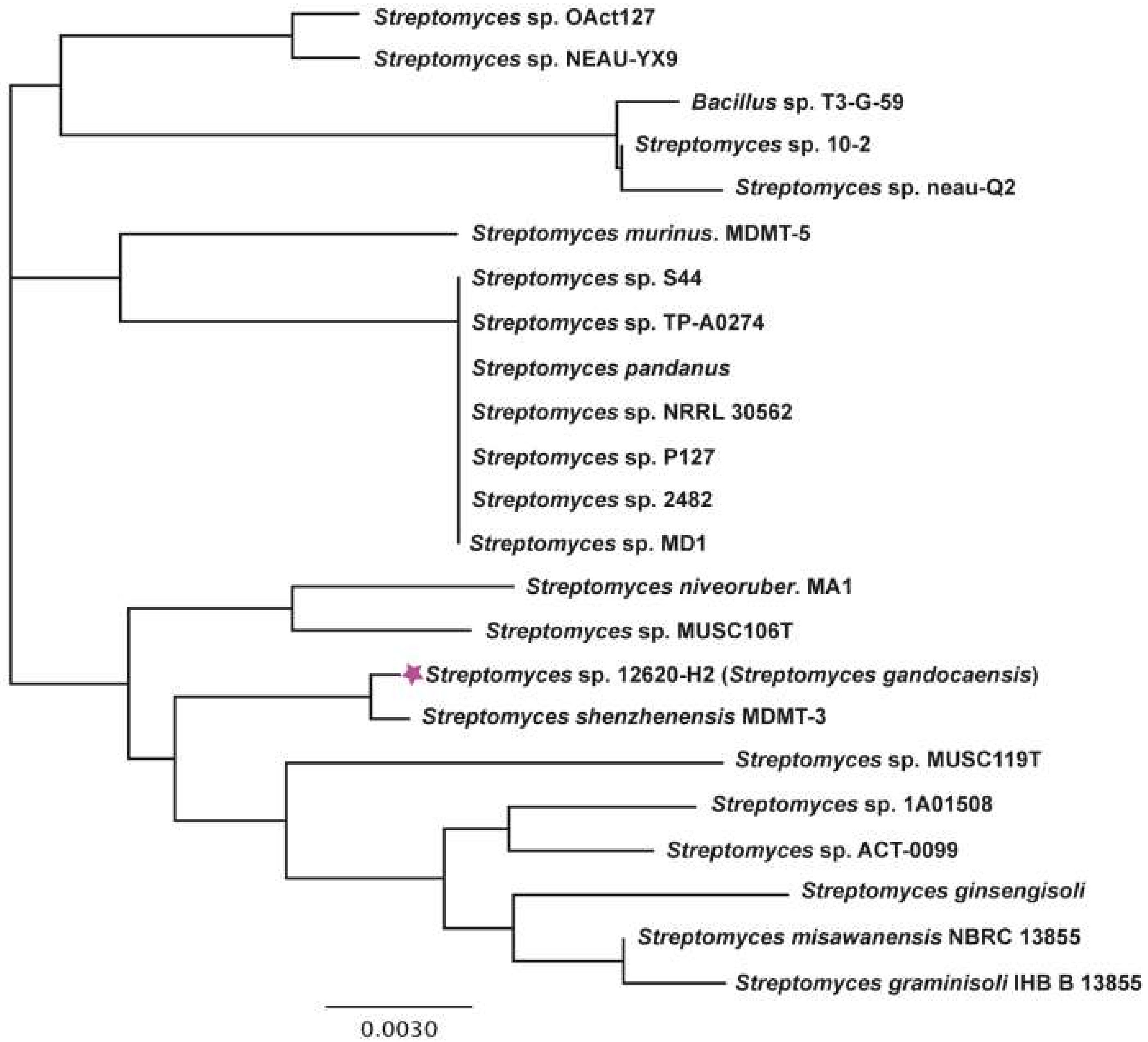

Supplementary Figure 6: Phylogenetic analysis of the 16S rDNA gene sequences of cahuitamycin producing-*Streptomyces gandocaensis*

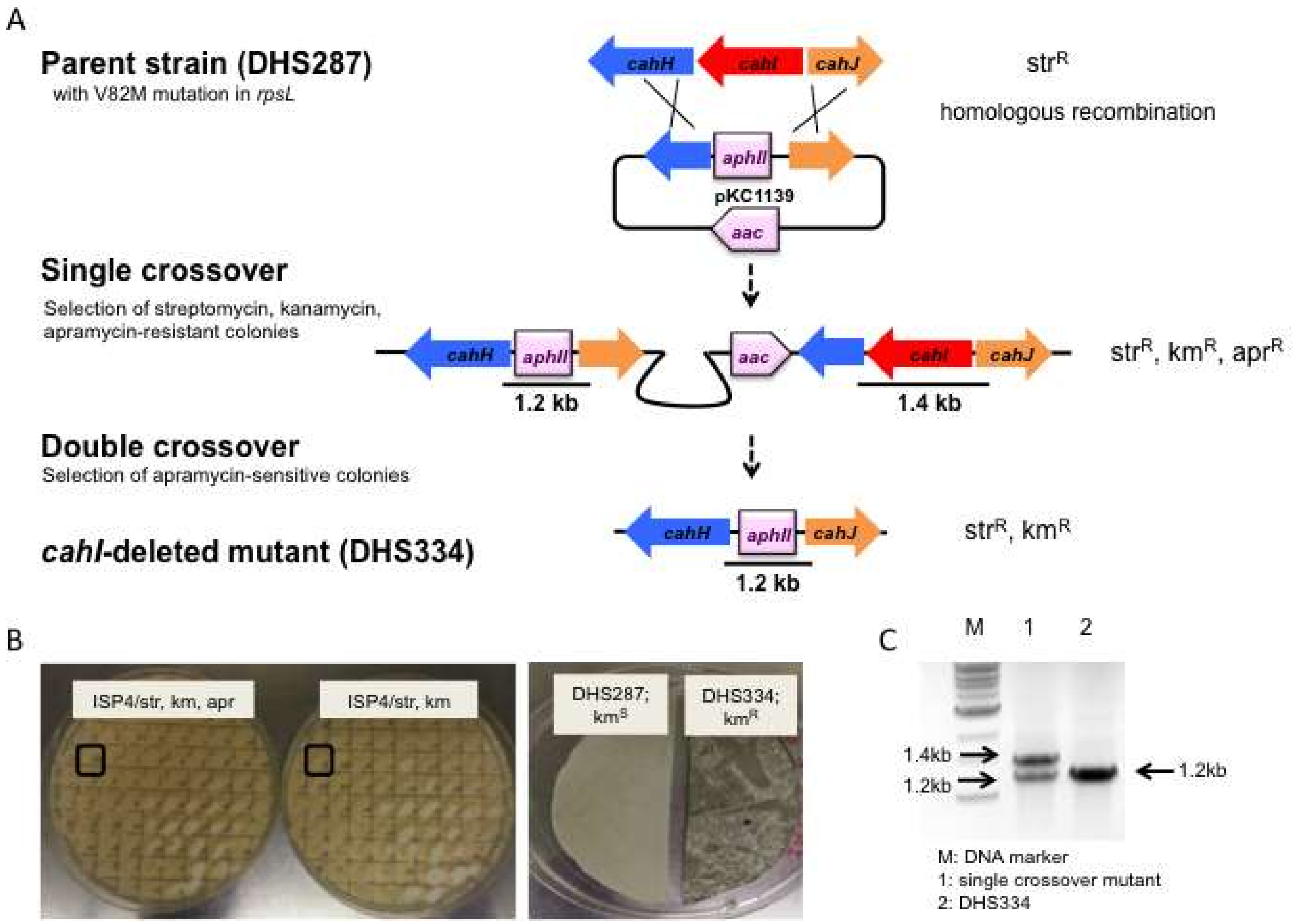

Supplementary Figure 7. Replacement of the *cahI* gene with kanamycin-resistance gene by homologous recombination. A) A single crossover between pSRP50 (see the experimental section) and a homologous DNA in the genome of the ribosome-engineered *S. gandocaensis* (DHS287; streptomycin-resistant (str^R)) gave the pSRP50-integrated strain, and a second crossover generated a *cahI*-disrupted strain DHS334 (streptomycin- and kanamycin-resistant (km^R)). B) Selection of (str^R) and (km^R) colonies. C) Confirmation of genotype of the DHS334 by PCR. Gene abbreviations: *aphII*, kanamycin resistance cassette; *aac*, apramycin resistance cassette.

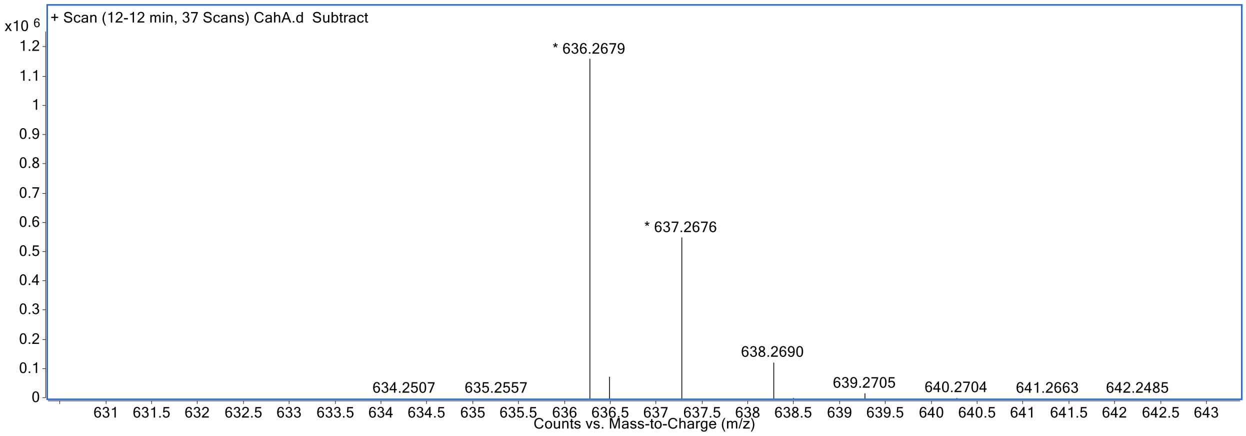

Supplementary Figure 8. HRESIMS chromatogram of cahuitamycin A (**1**).

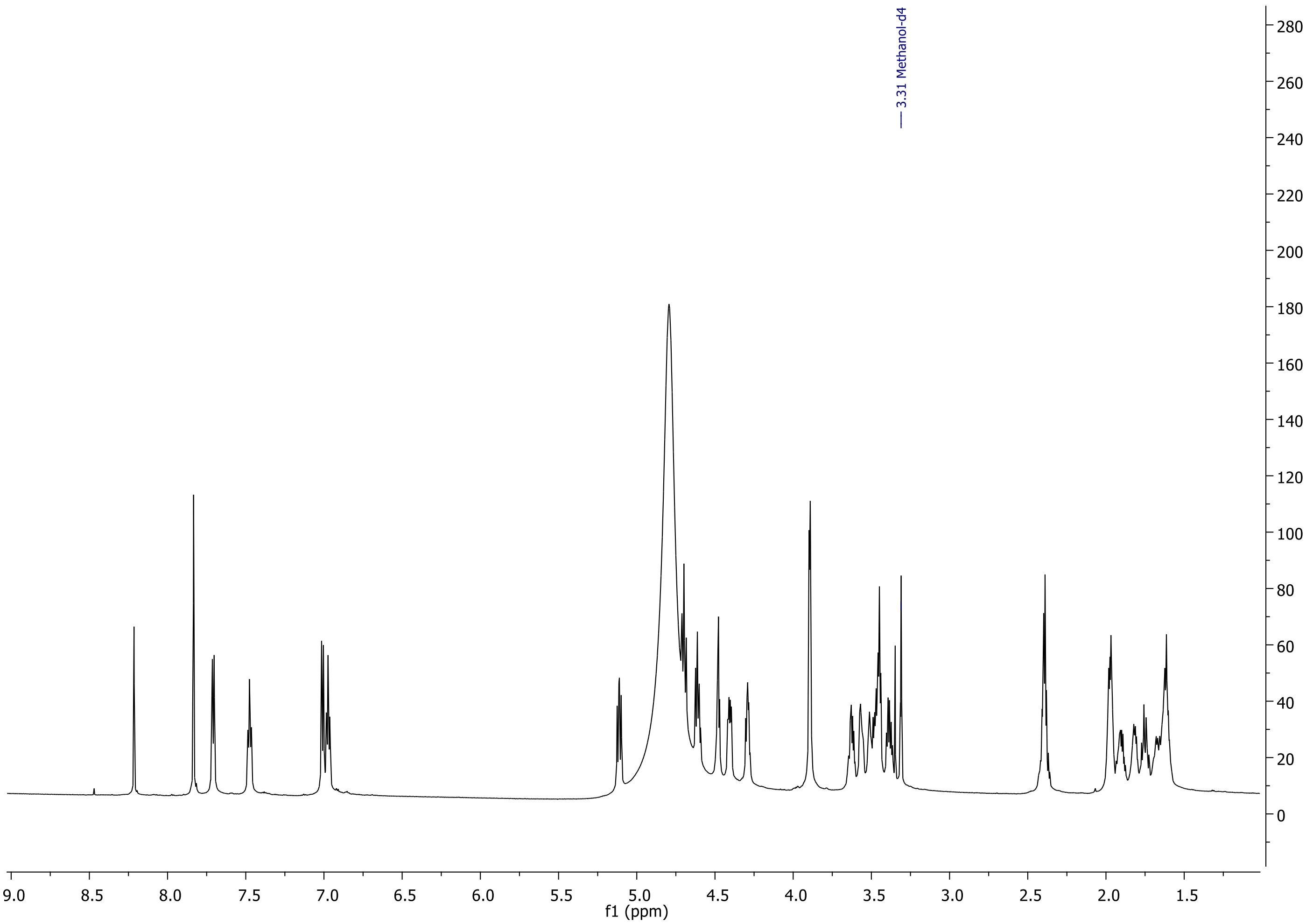

Supplementary Figure 9: ^{1}H NMR (MHz, CD_3OD+D_2O) spectrum of cahuitamycin A (**1**).

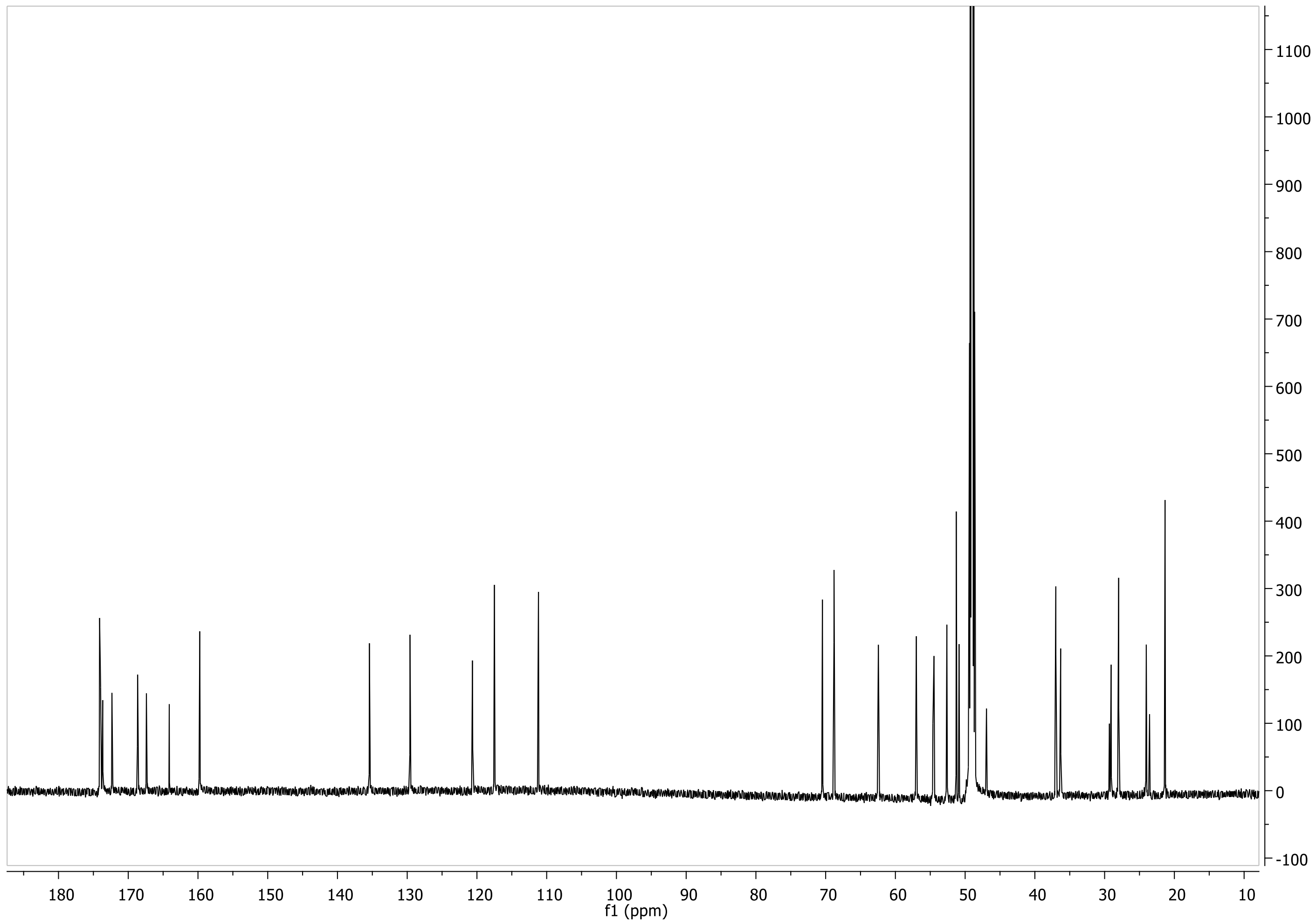

Supplementary Figure 10: ^{13}C NMR (MHz, CD_3OD+D_2O) spectrum of cahuitamycin A (**1**).

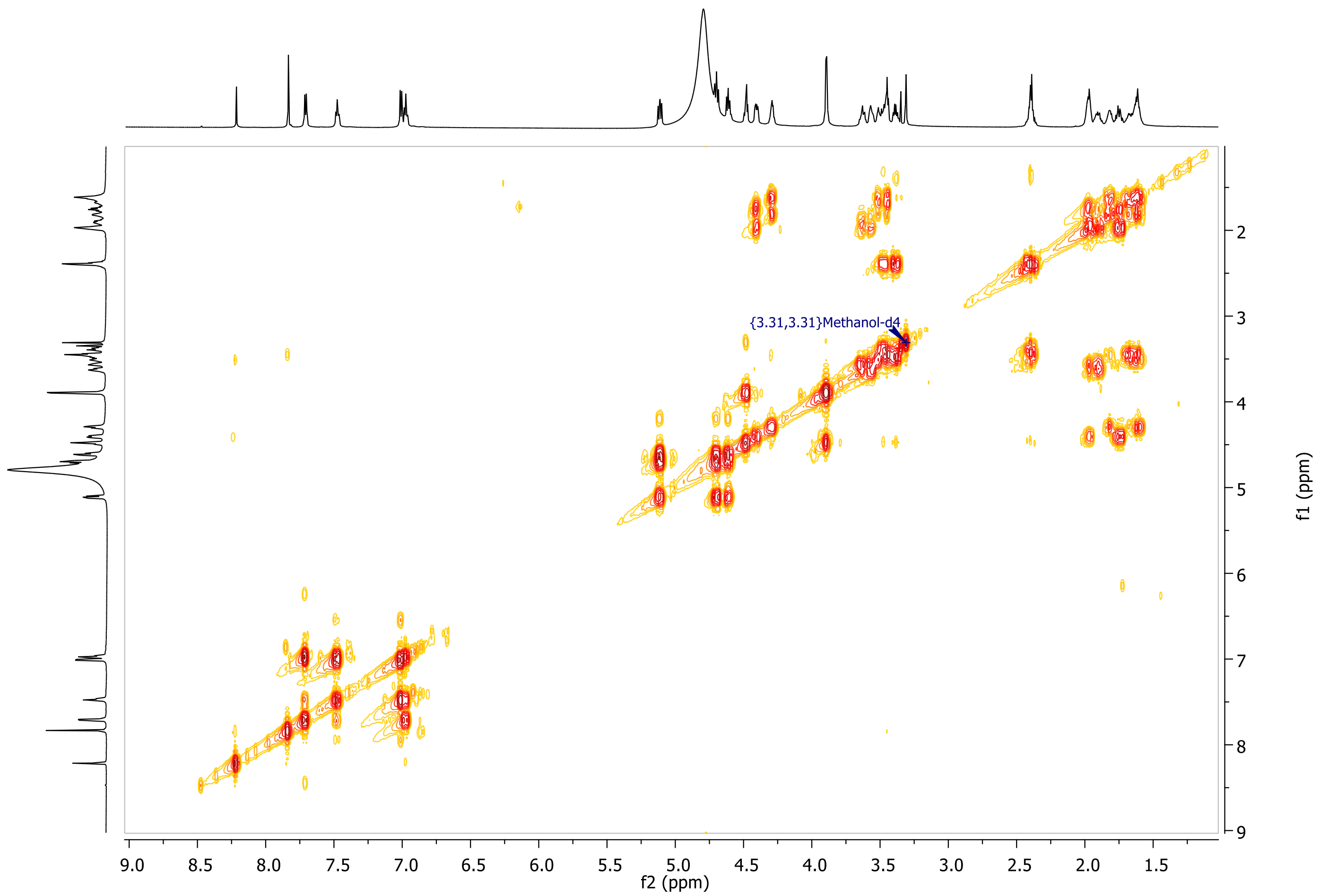

Supplementary Figure 11: ^{1}H-^{1}H COSY NMR spectrum of cahuitamycin A (**1**).

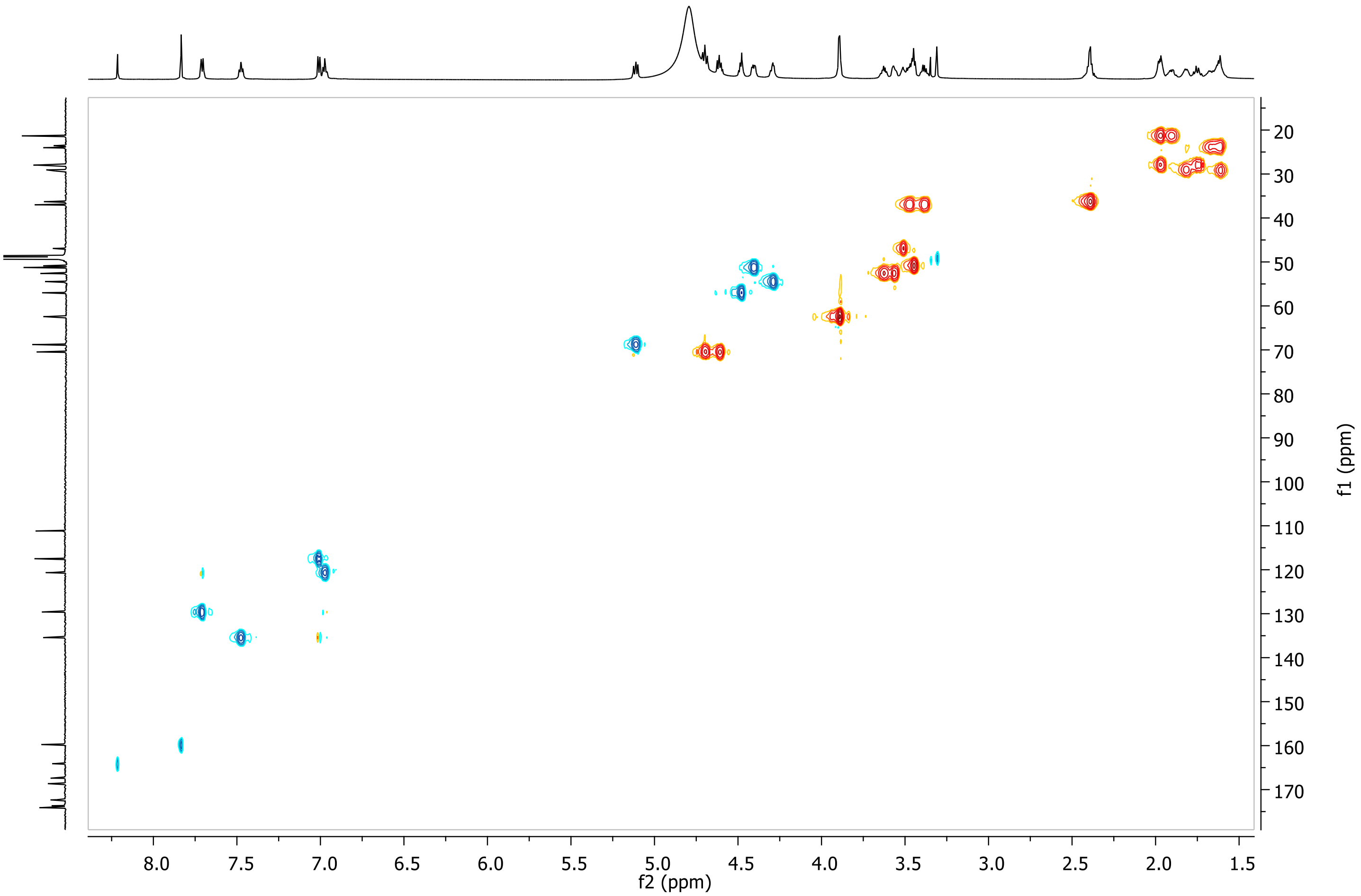

Supplementary Figure 12: 2D HSQC NMR spectrum of cahuitamycin A (**1**).

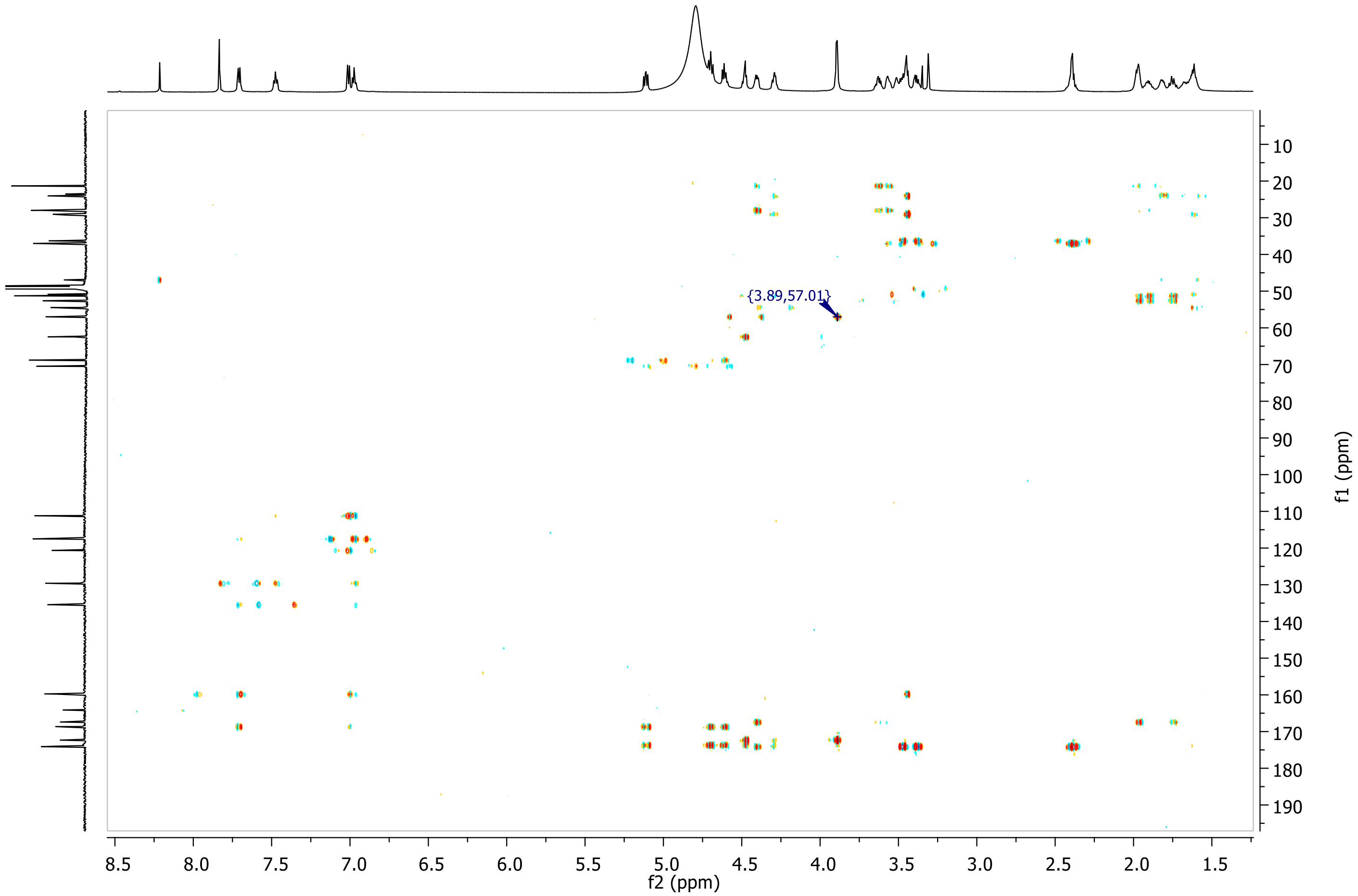

Supplementary Figure 13: 2D HMBC NMR spectrum of cahuitamycin A (**1**).

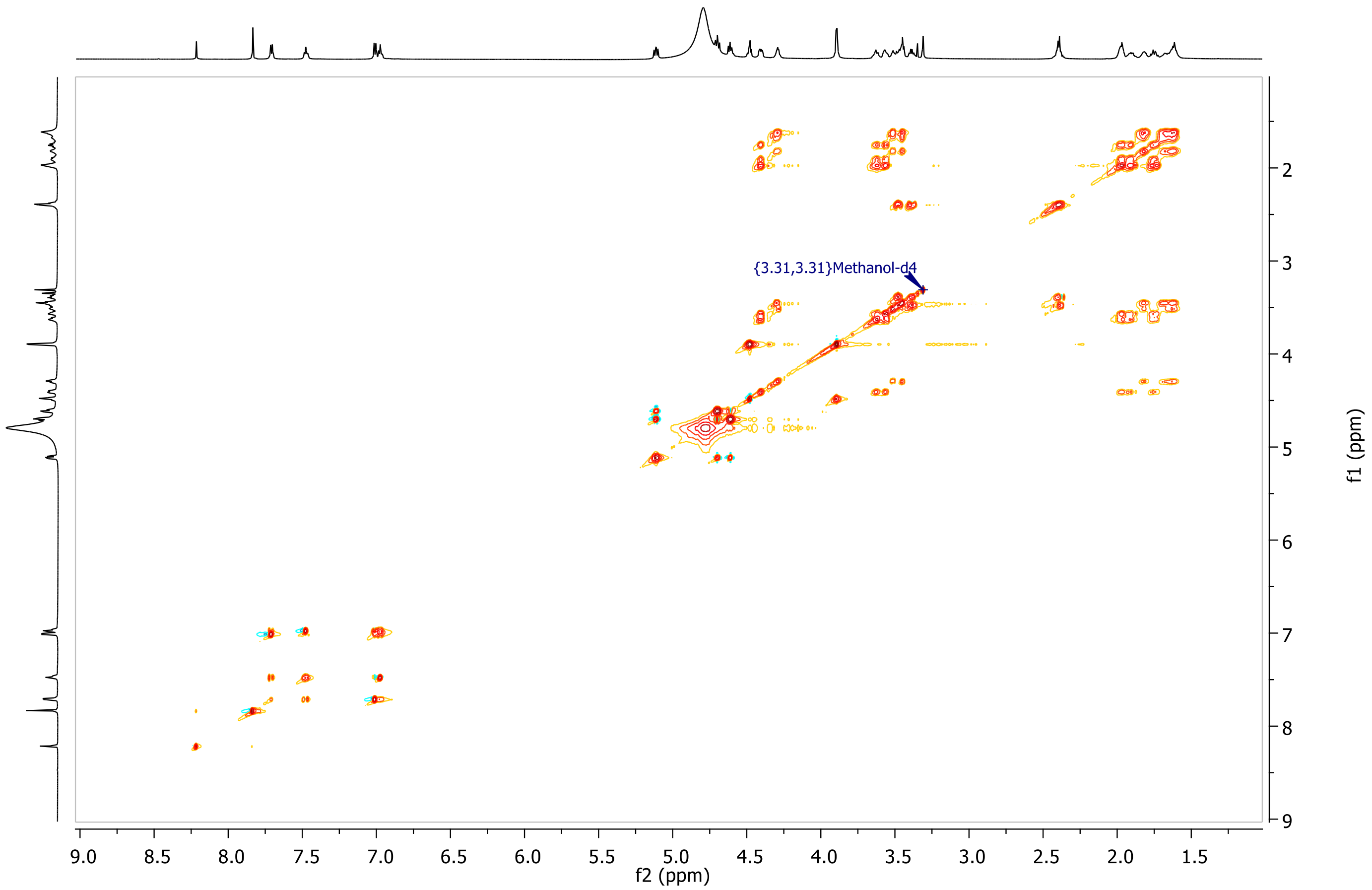

Supplementary Figure 14: 2D TOCSY NMR spectrum of cahuitamycin A (**1**).

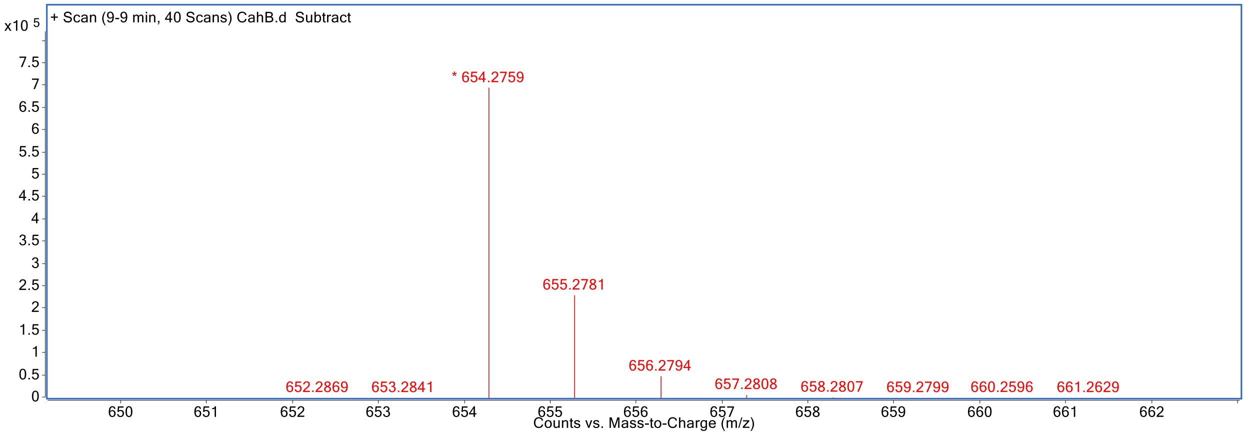

Supplementary Figure 15: HRMS chromatogram of cahuitamycin B (**2**).

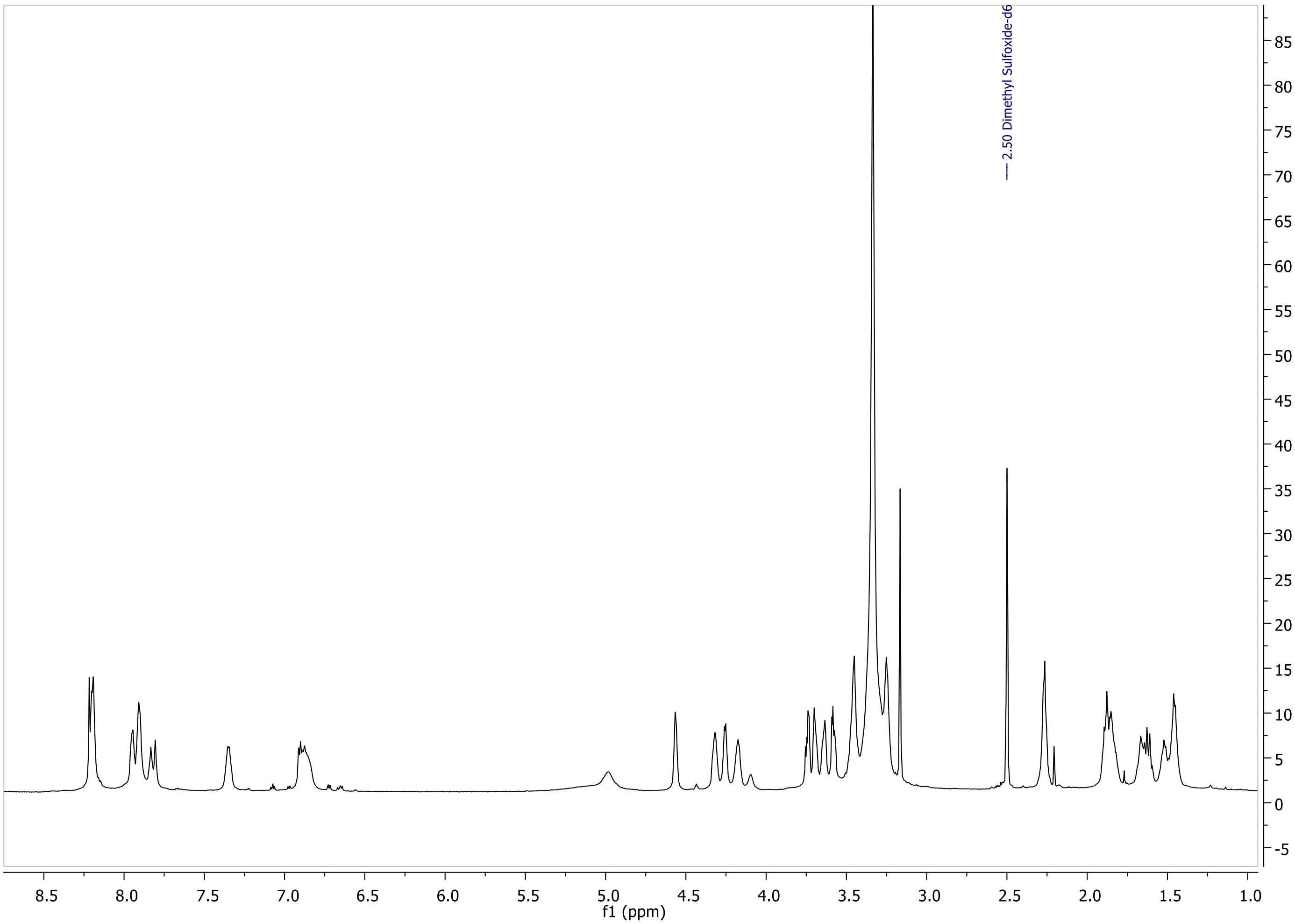

Supplementary Figure 16: ^{1}H NMR (MHz, DMSO-d6) spectrum of cahuitamycin B (**2**).

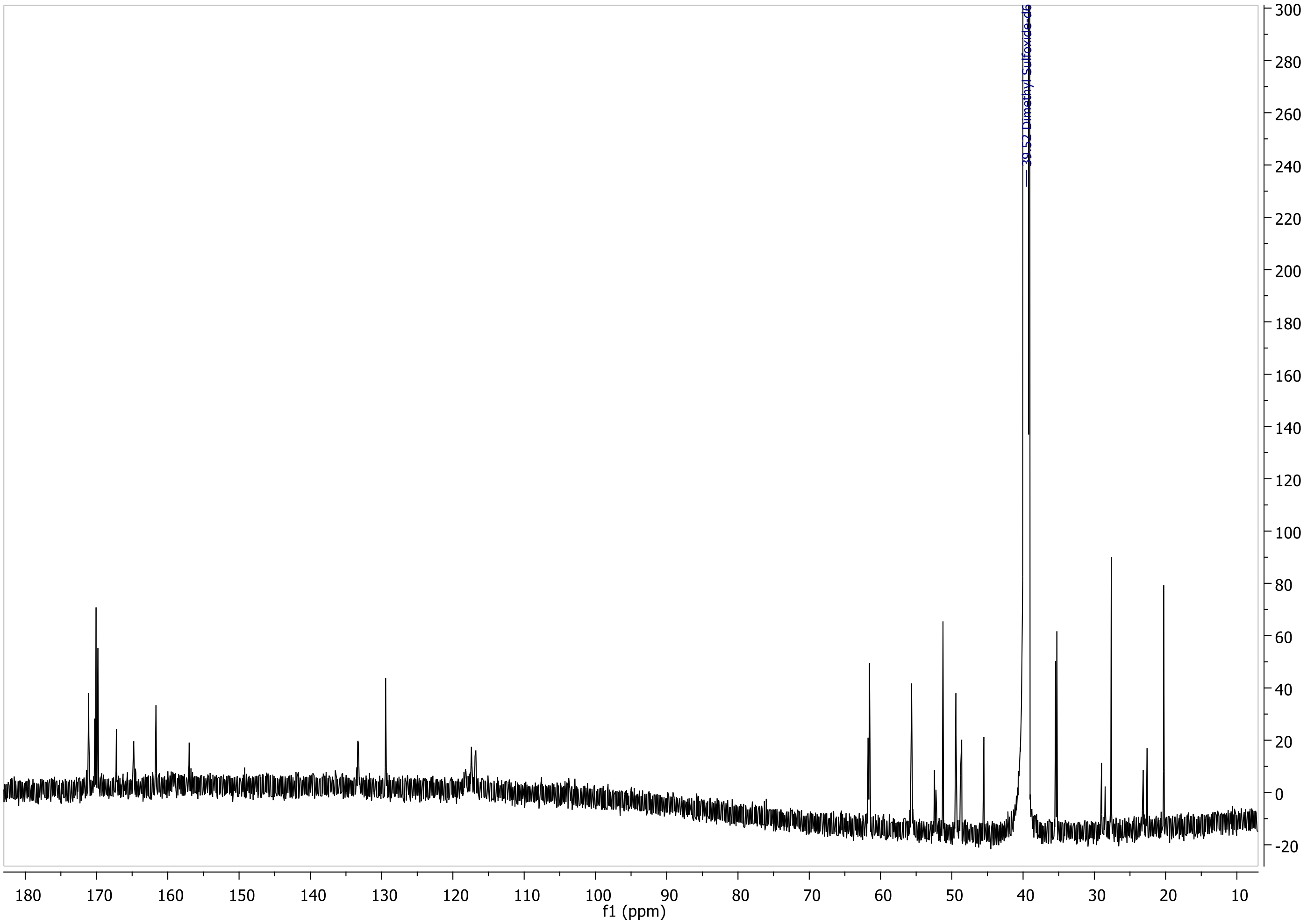

Supplementary Figure 17: ^{13}C NMR (MHz, DMSO-d6) spectrum of cahuitamycin B (**2**).

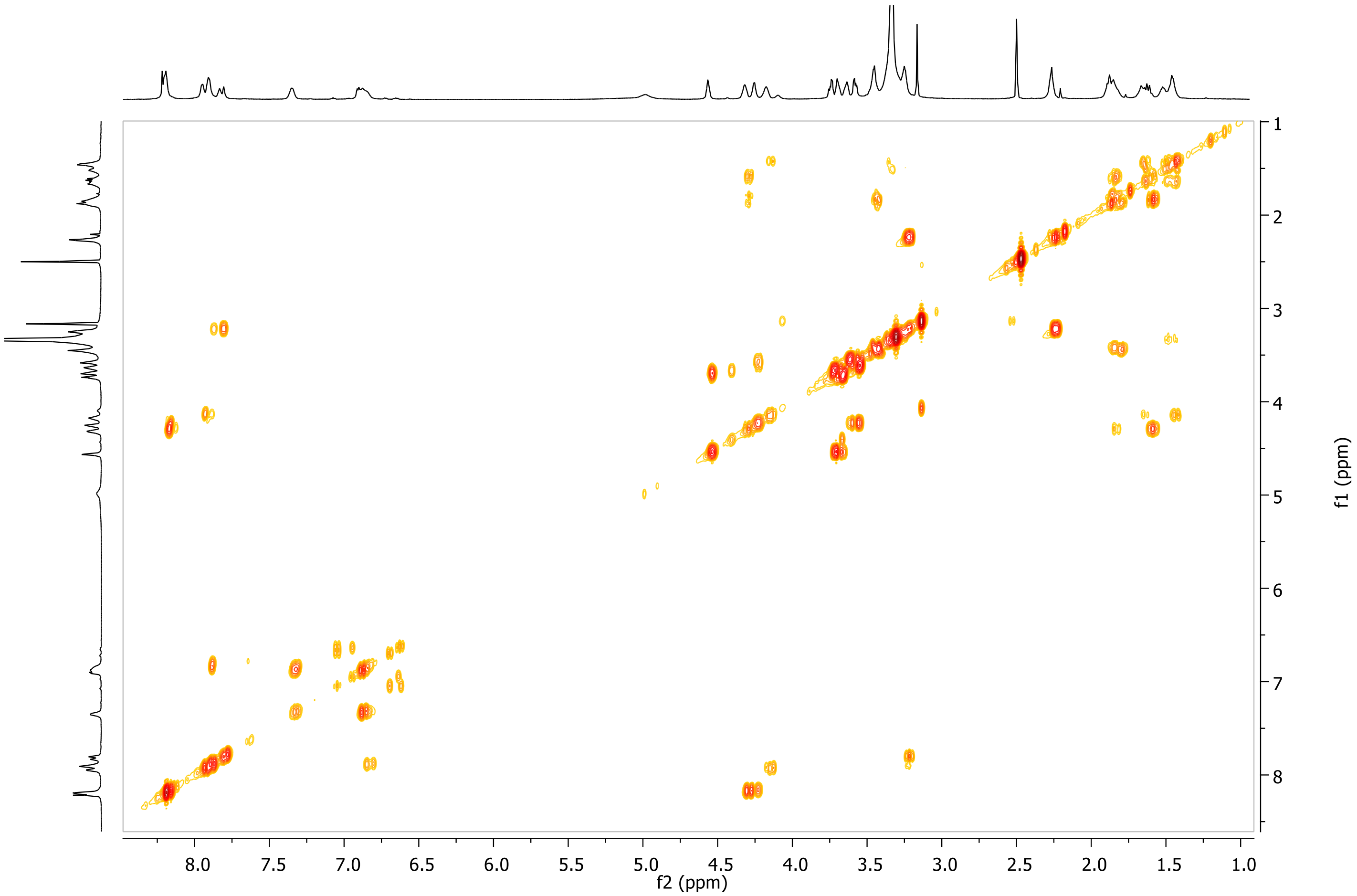

Supplementary Figure 18: ^{1}H-^{1}H COSY NMR spectrum of cahuitamycin B (**2**).

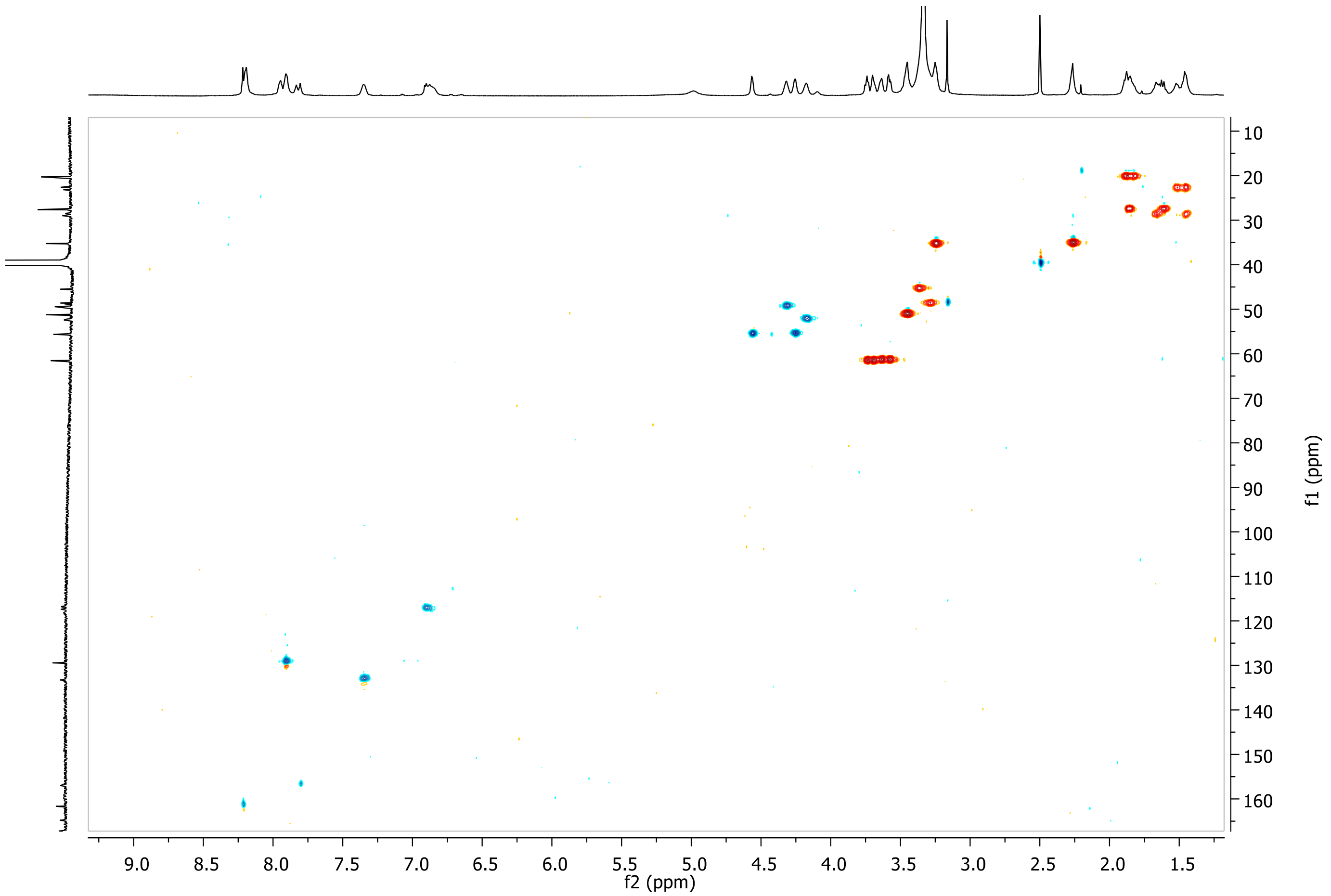

Supplementary Figure 19: 2D HSQC NMR spectrum of cahuitamycin B (**2**).

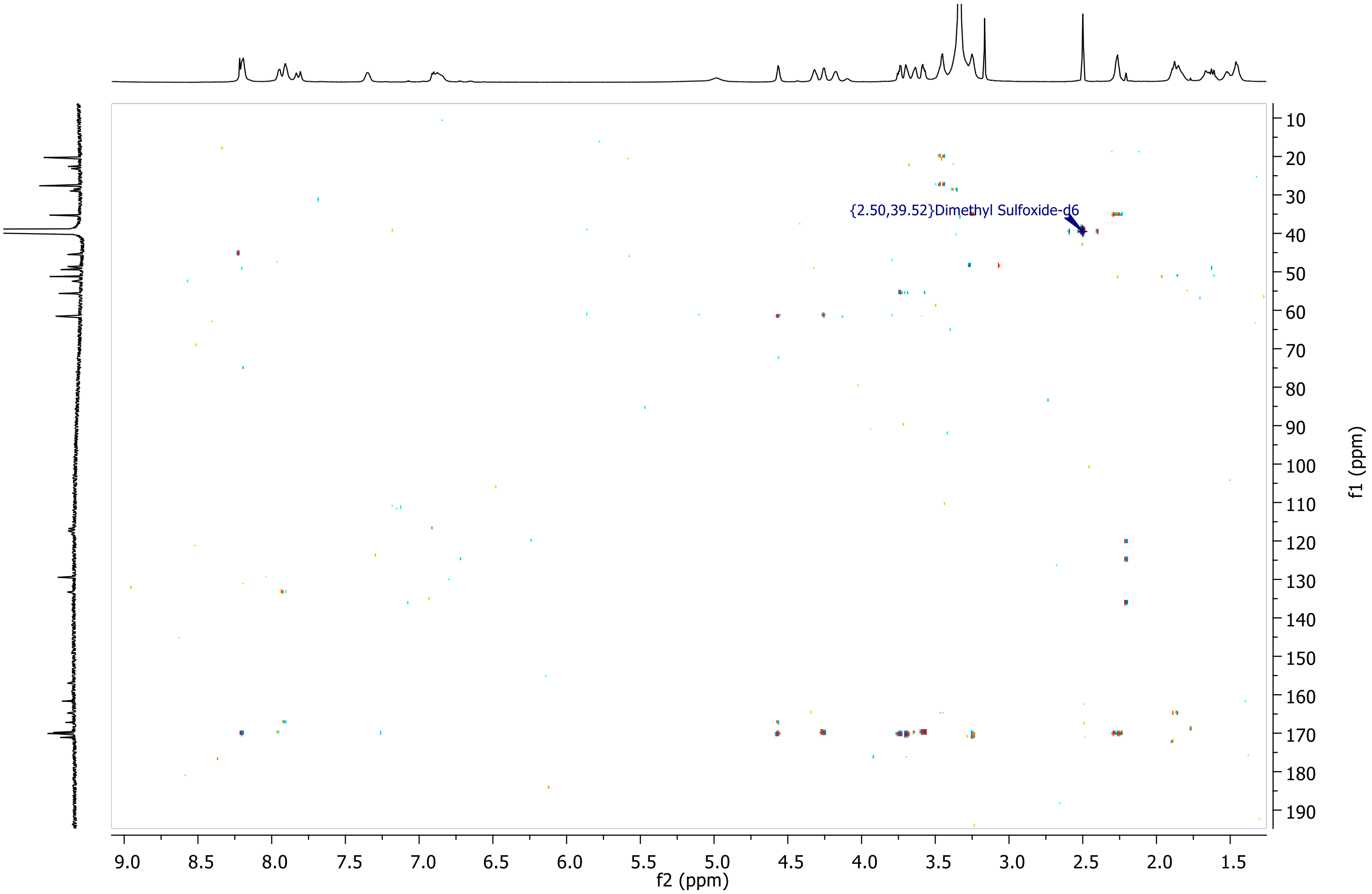

Supplementary Figure 20: 2D HMBC NMR spectrum of cahuitamycin B (**2**).

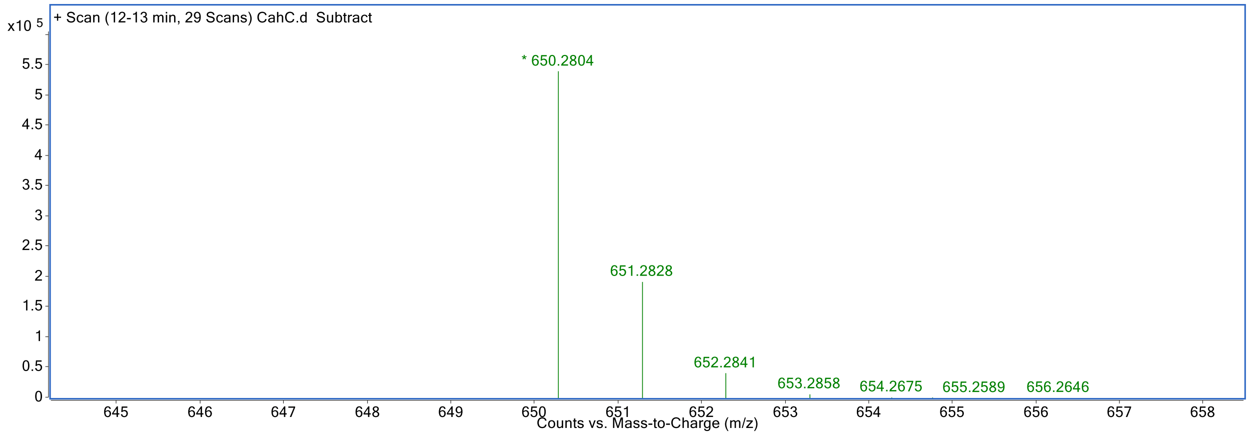

Supplementary Figure 21: HRMS chromatogram of cahuitamycin C (**3**).

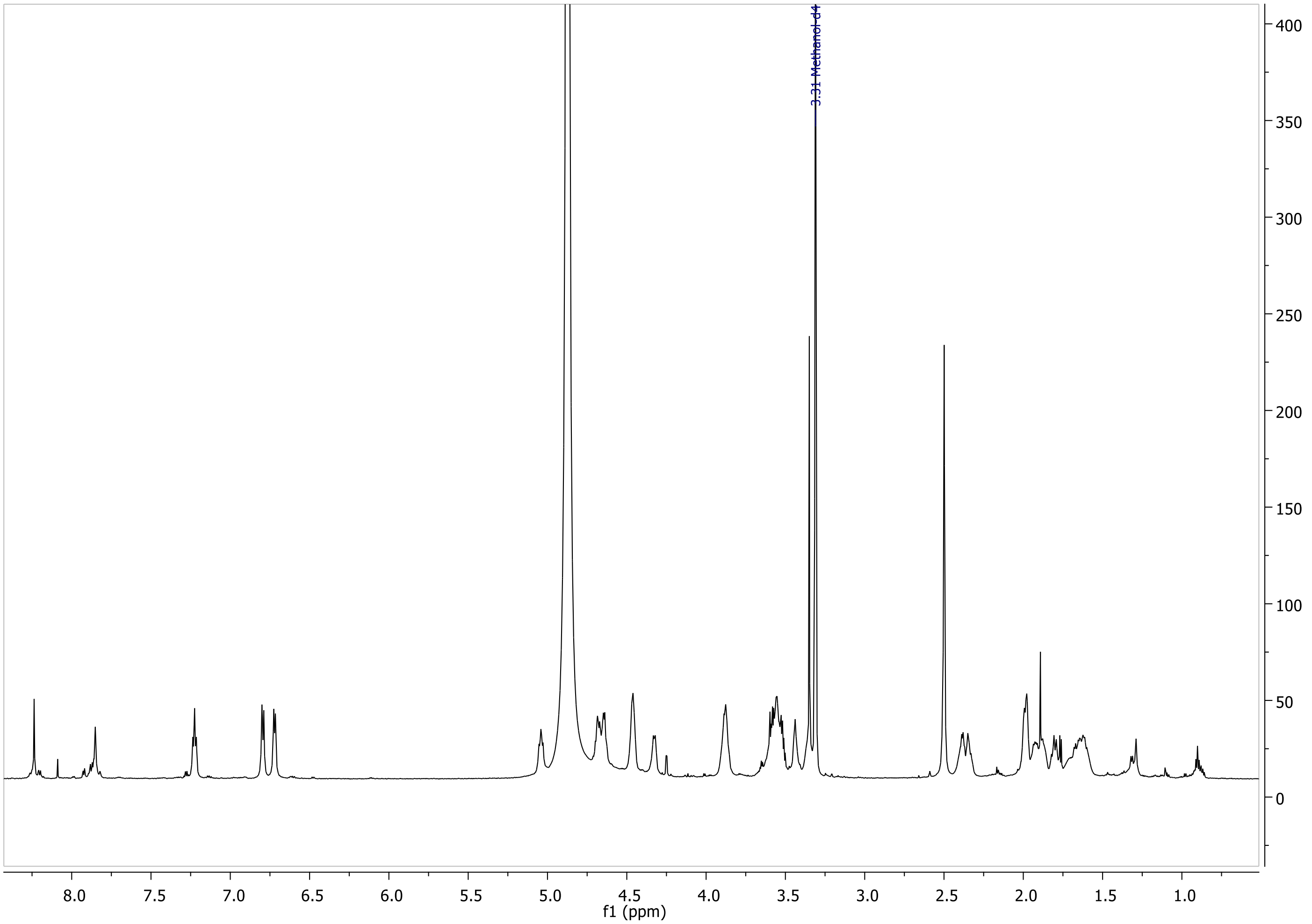

Supplementary Figure 22: ^{1}H NMR (MHz, CD_3OD) spectrum of cahuitamycin C (**3**).

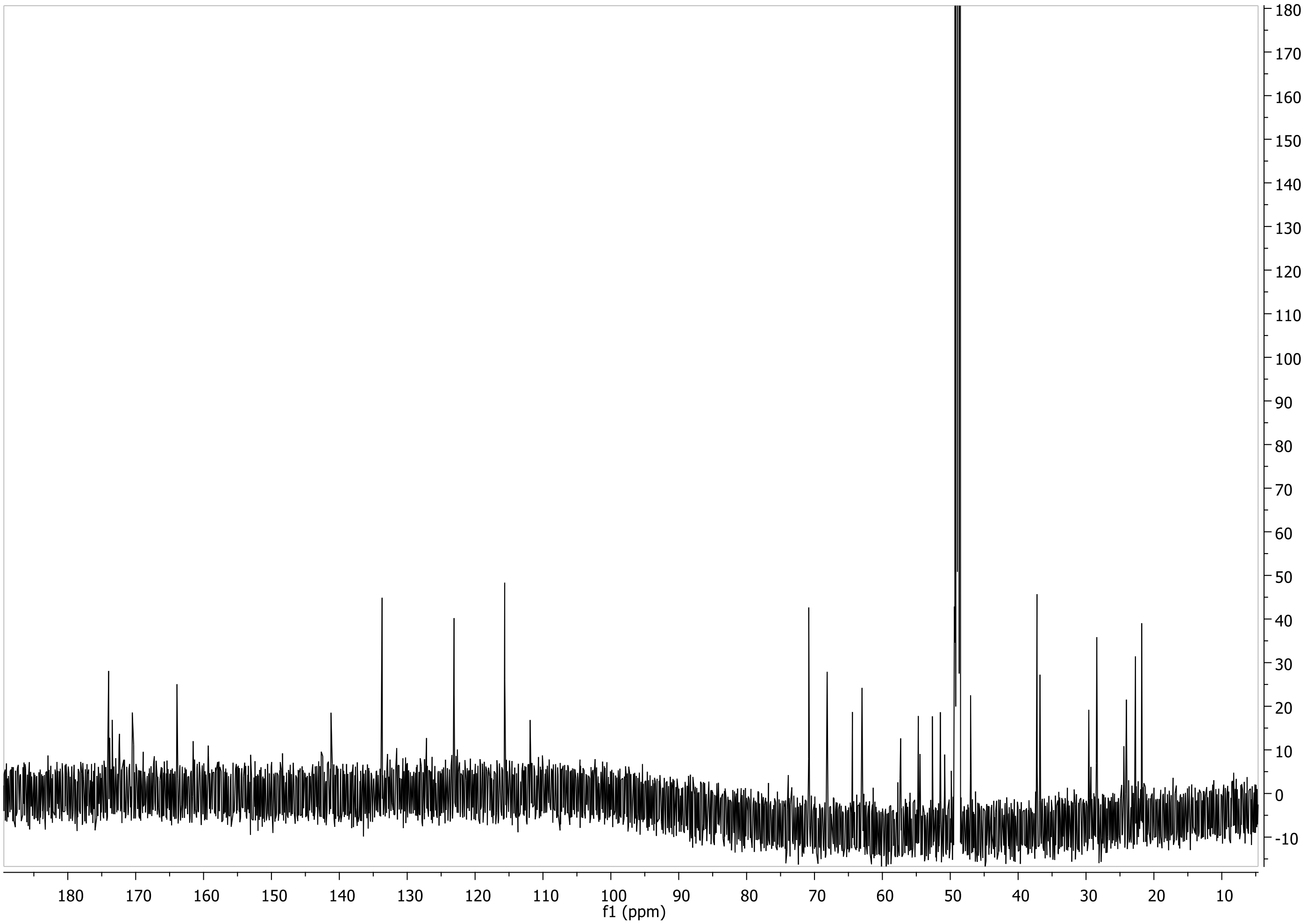

Supplementary Figure 23: ^{13}C NMR (MHz, CD_3OD+D_2O) spectrum of cahuitamycin C (**3**).

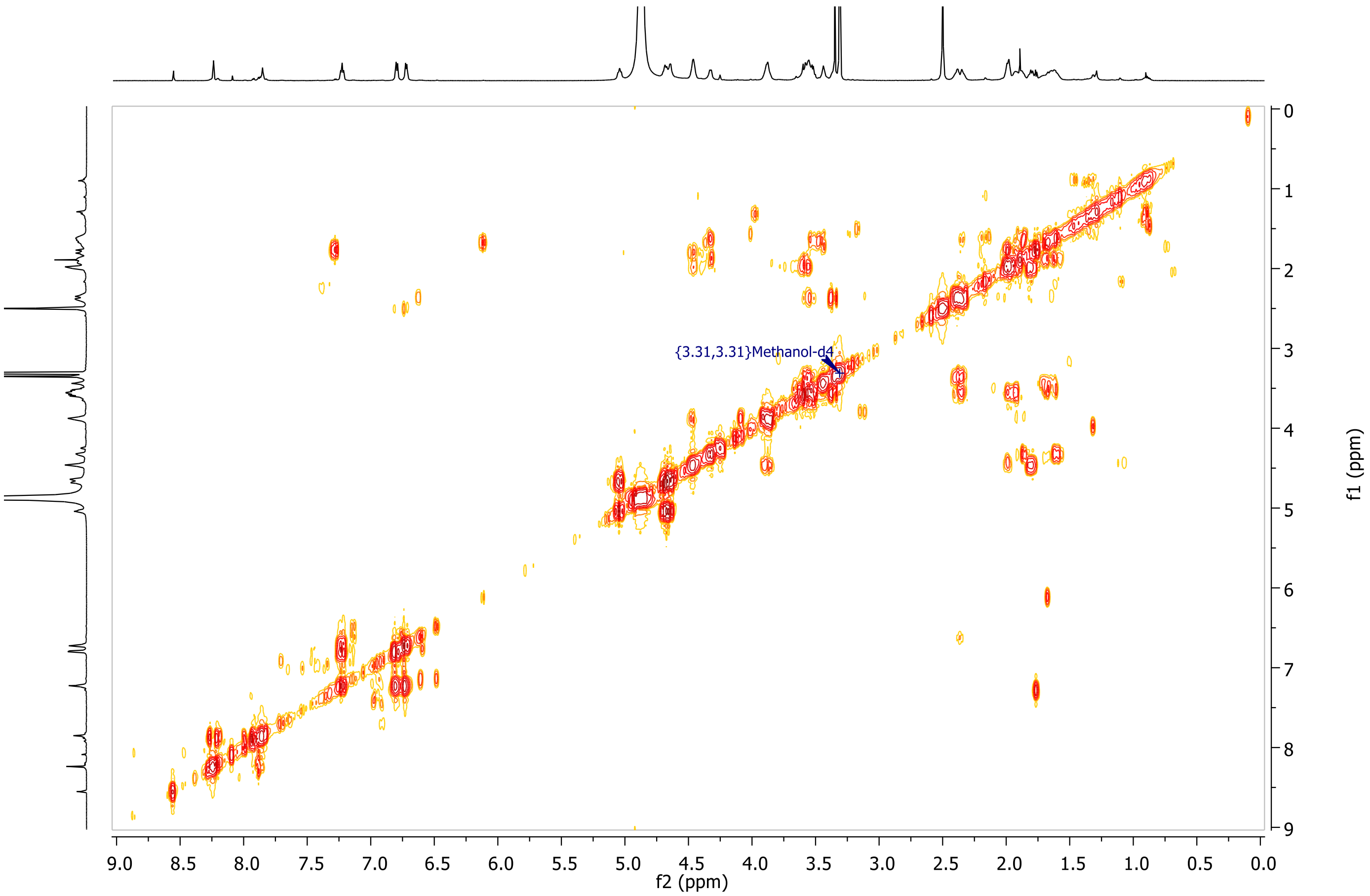

Supplementary Figure 24: ^{1}H-^{1}H COSY NMR spectrum of cahuitamycin C (**3**).

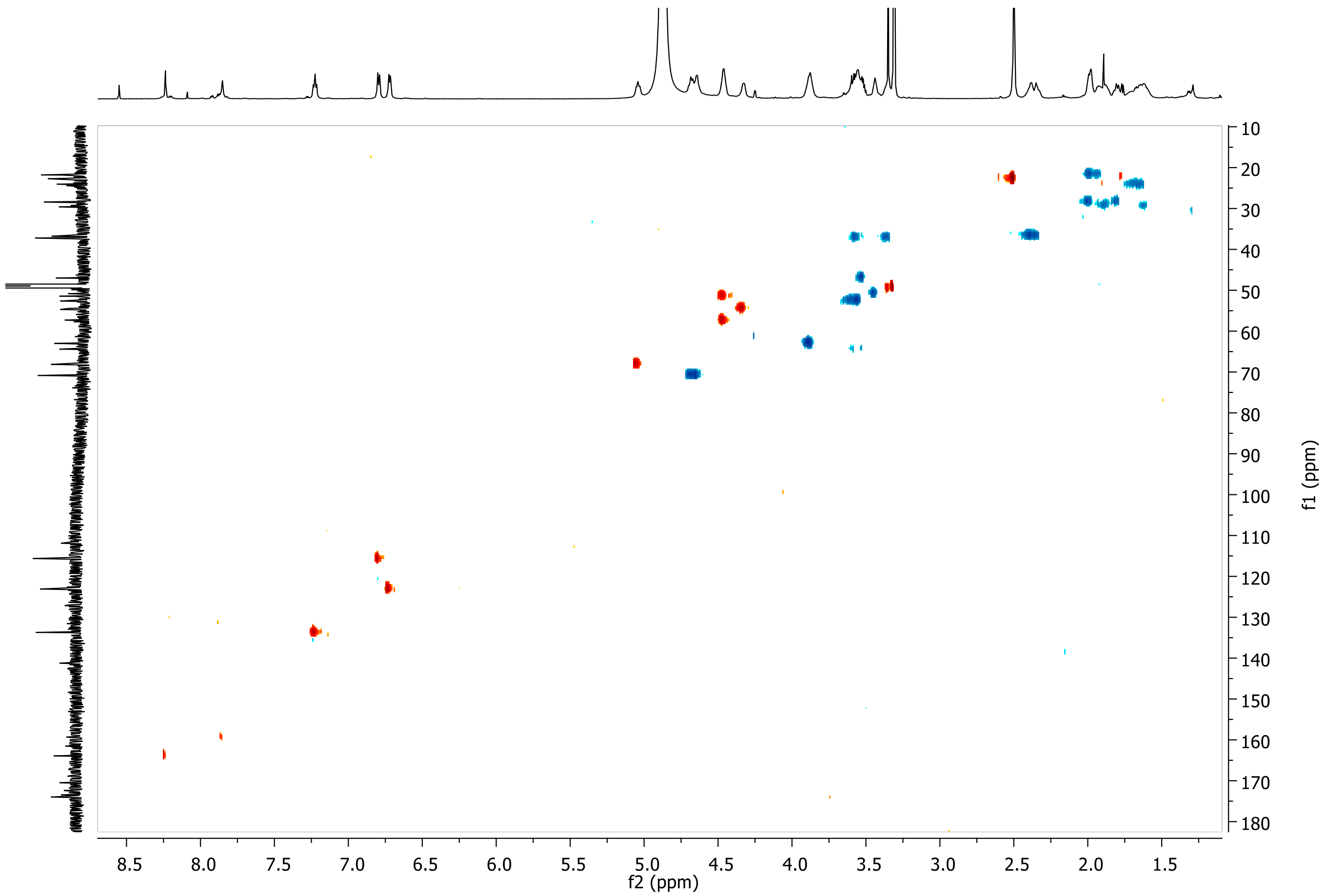

Supplementary Figure 25: 2D HSQC NMR spectrum of cahuitamycin C (**3**).

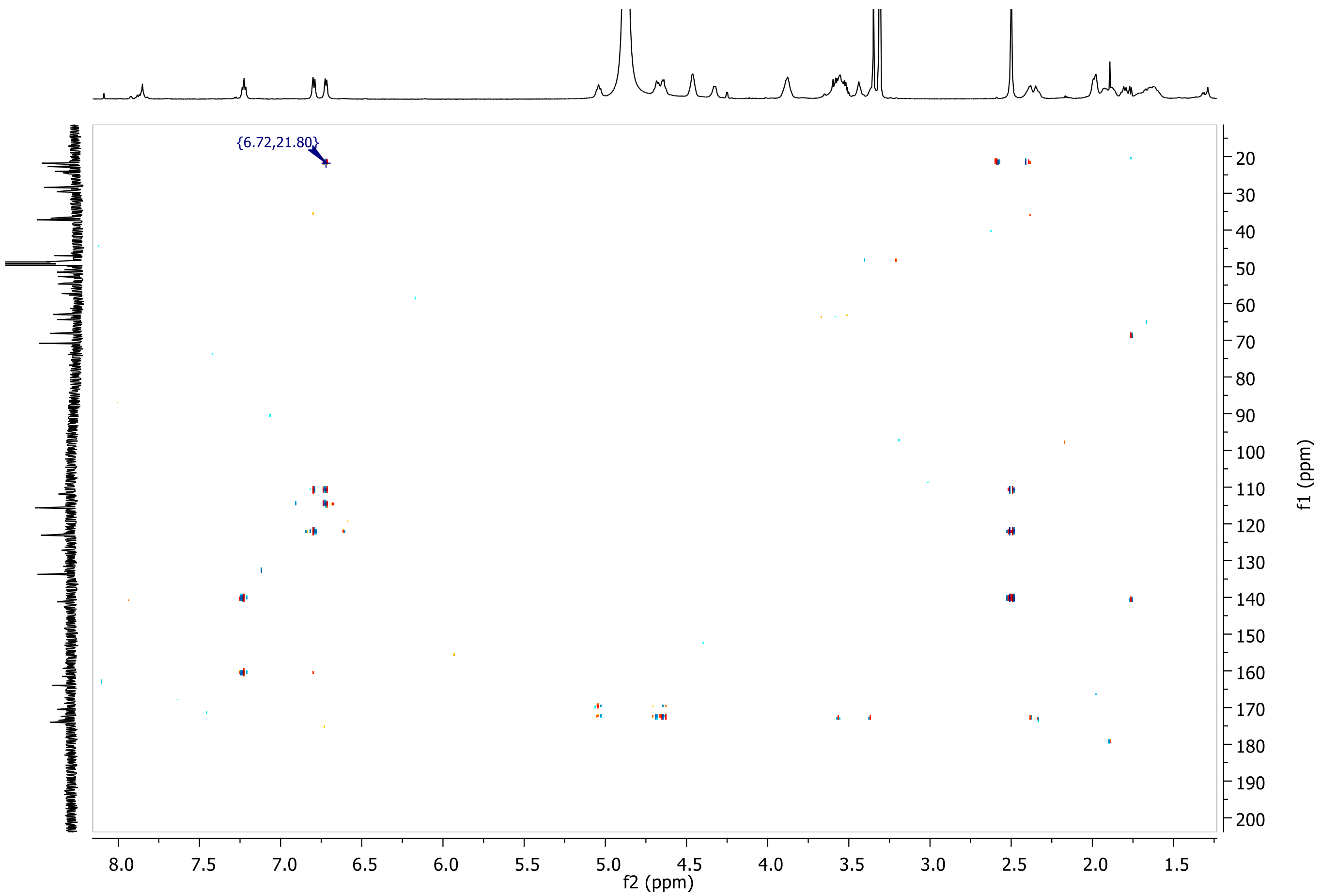

Supplementary Figure 26: 2D HMBC NMR spectrum of cahuitamycin C (**3**).

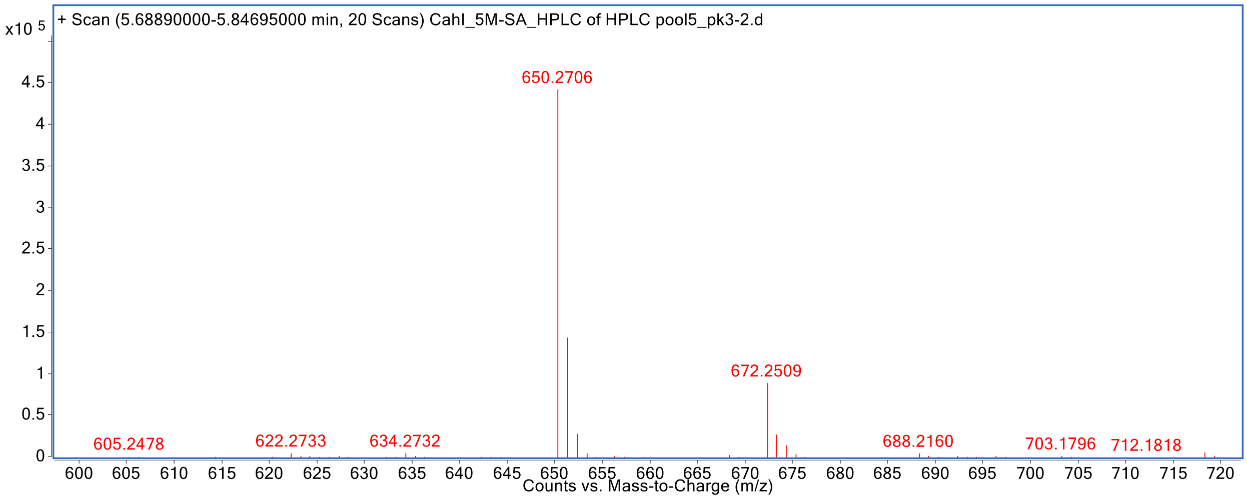

Supplementary Figure 27: HRMS chromatogram of cahuitamycin D (**4**).

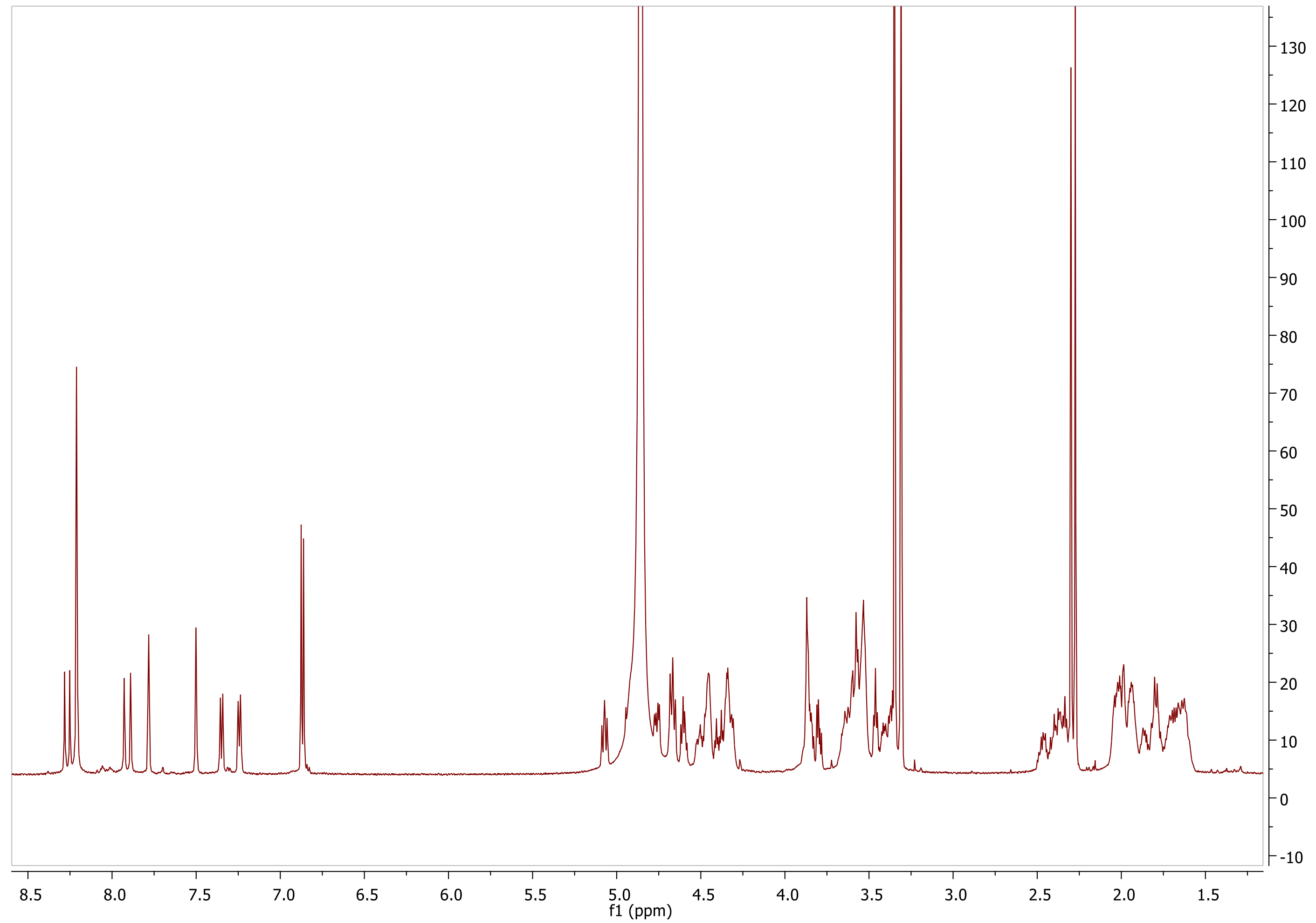

Supplementary Figure 28: ^{1}H NMR (600 MHz, CD_3OD) spectrum of cahuitamycin D (**4**).

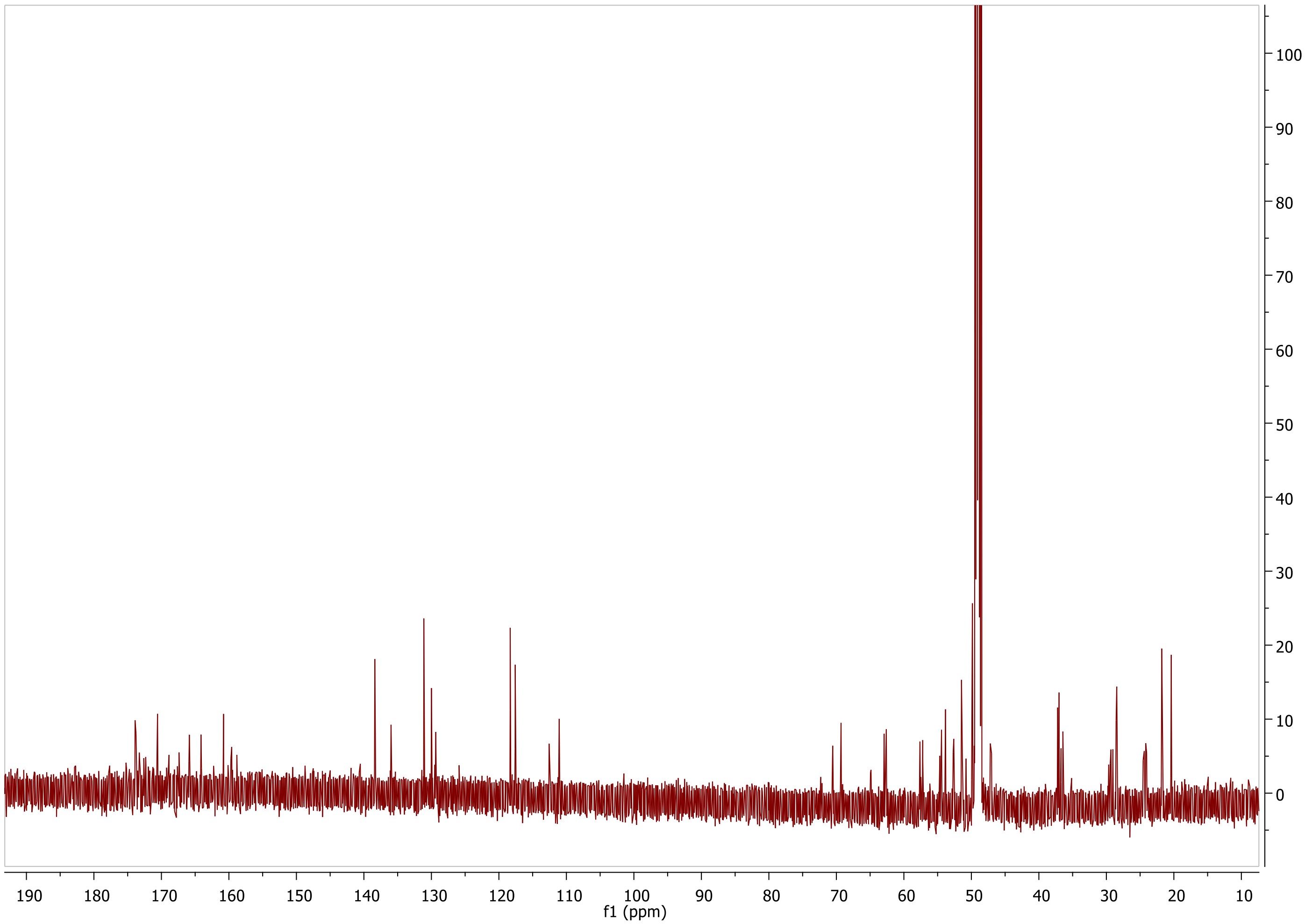

Supplementary Figure 29: ^{13}C NMR (600 MHz, CD_3OD) spectrum of cahuitamycin D (**4**).

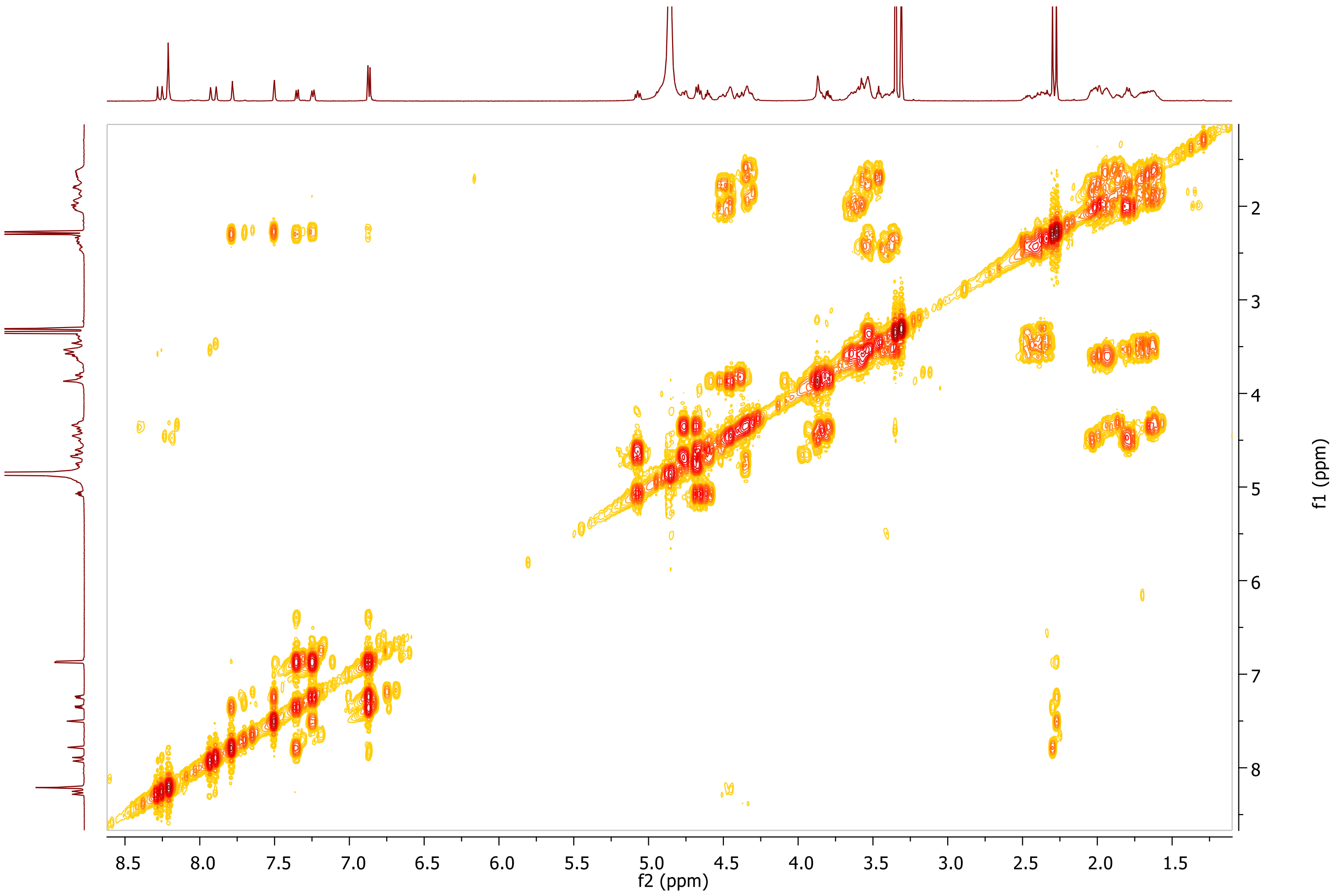

Supplementary Figure 30: ^{1}H-^{1}H COSY NMR spectrum of cahuitamycin D (**4**).

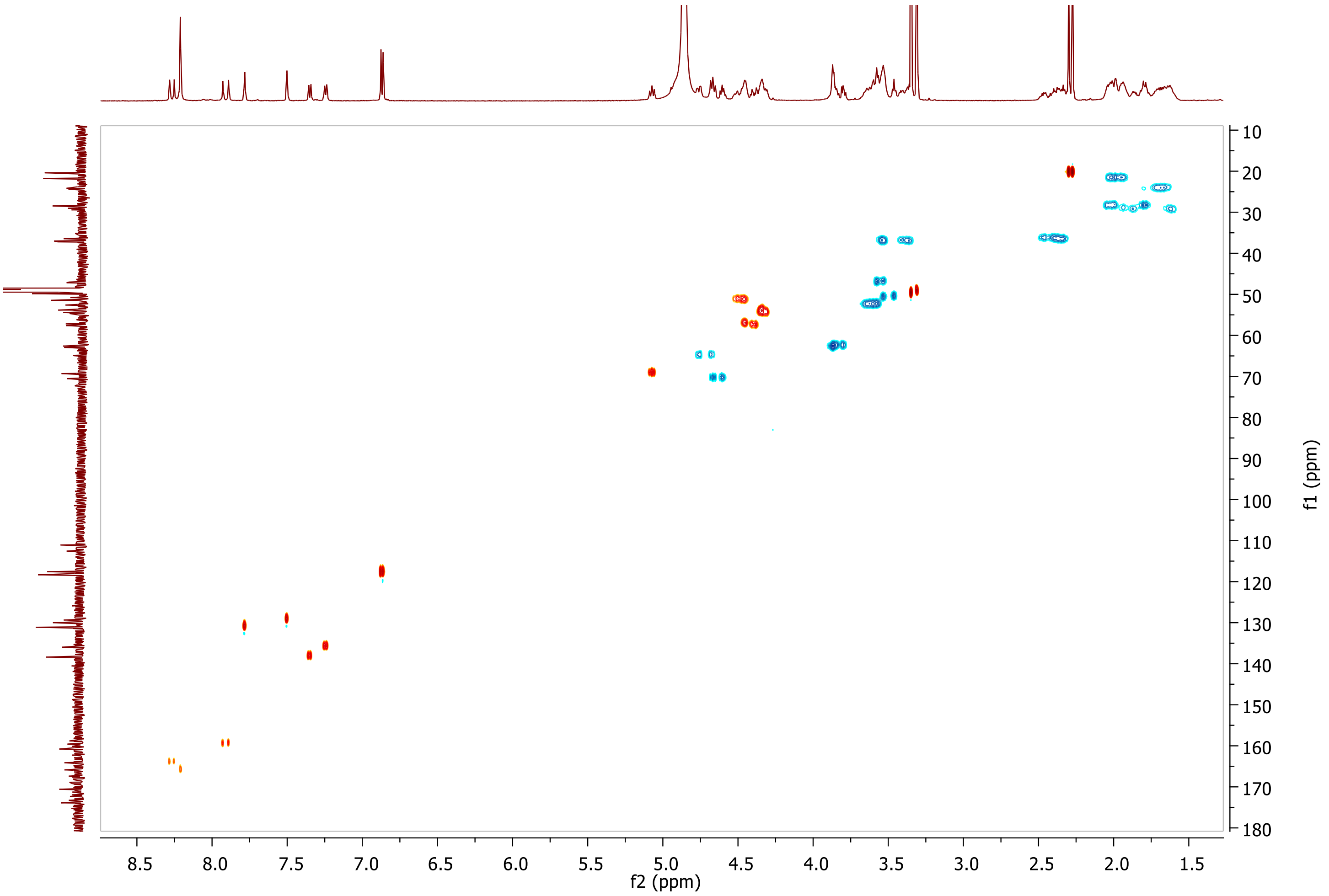

Supplementary Figure 31: 2D HSQC NMR spectrum of cahuitamycin D (**4**).

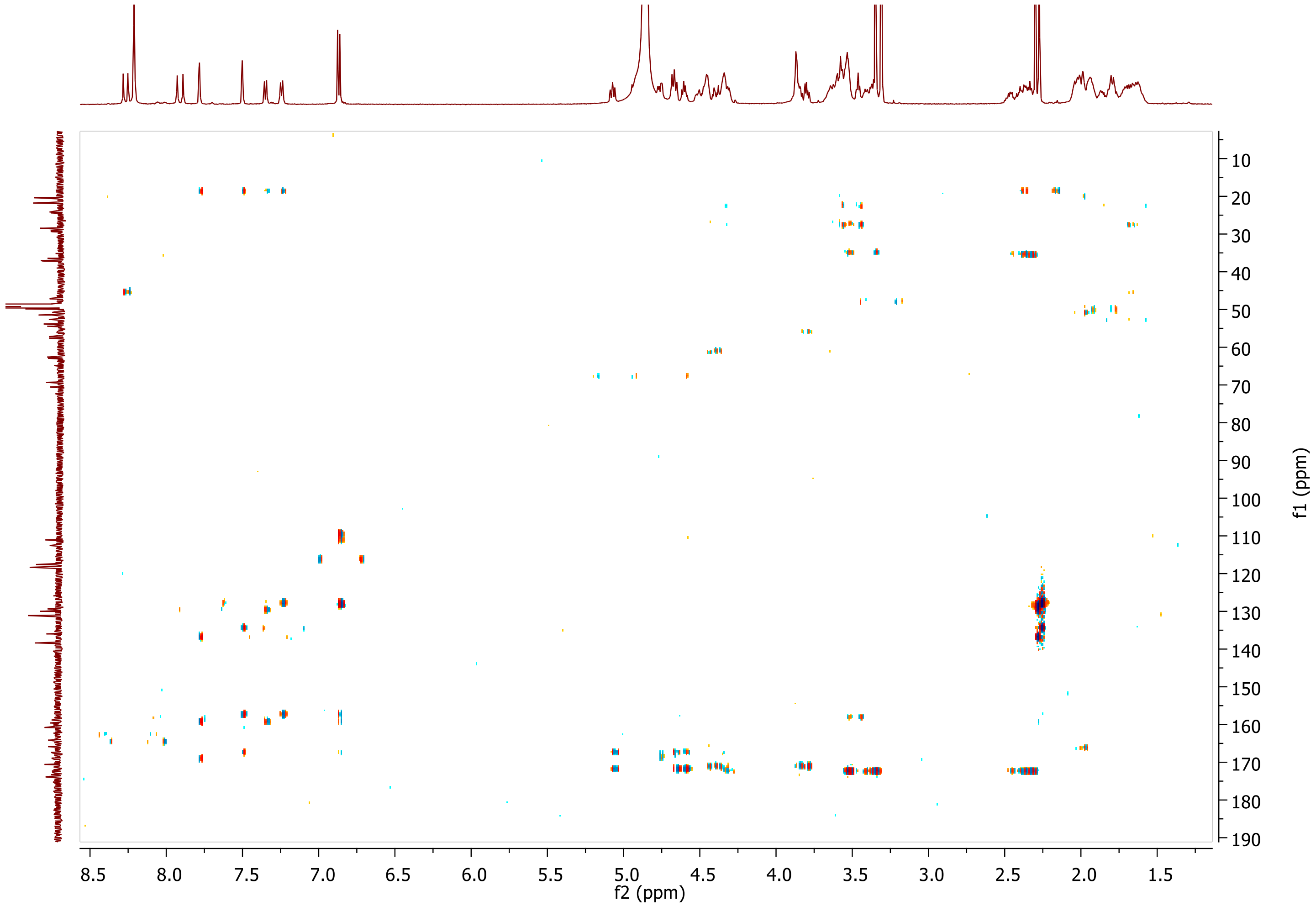

Supplementary Figure 32: 2D HMBC NMR spectrum of cahuitamycin D (**4**).

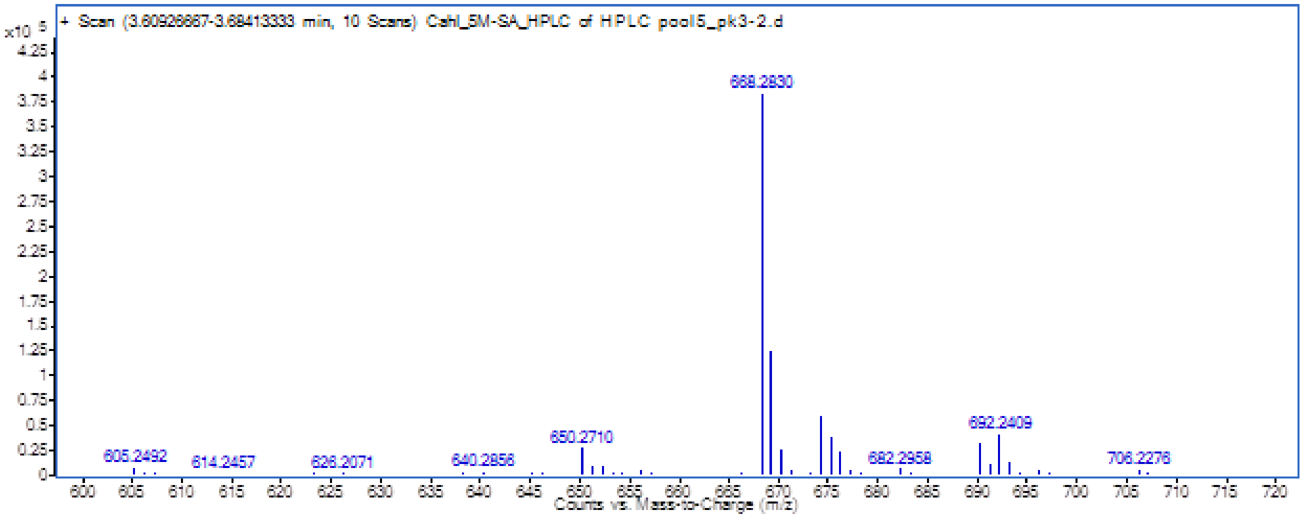

Supplementary Figure 33: HRMS chromatogram of cahuitamycin E (**5**).

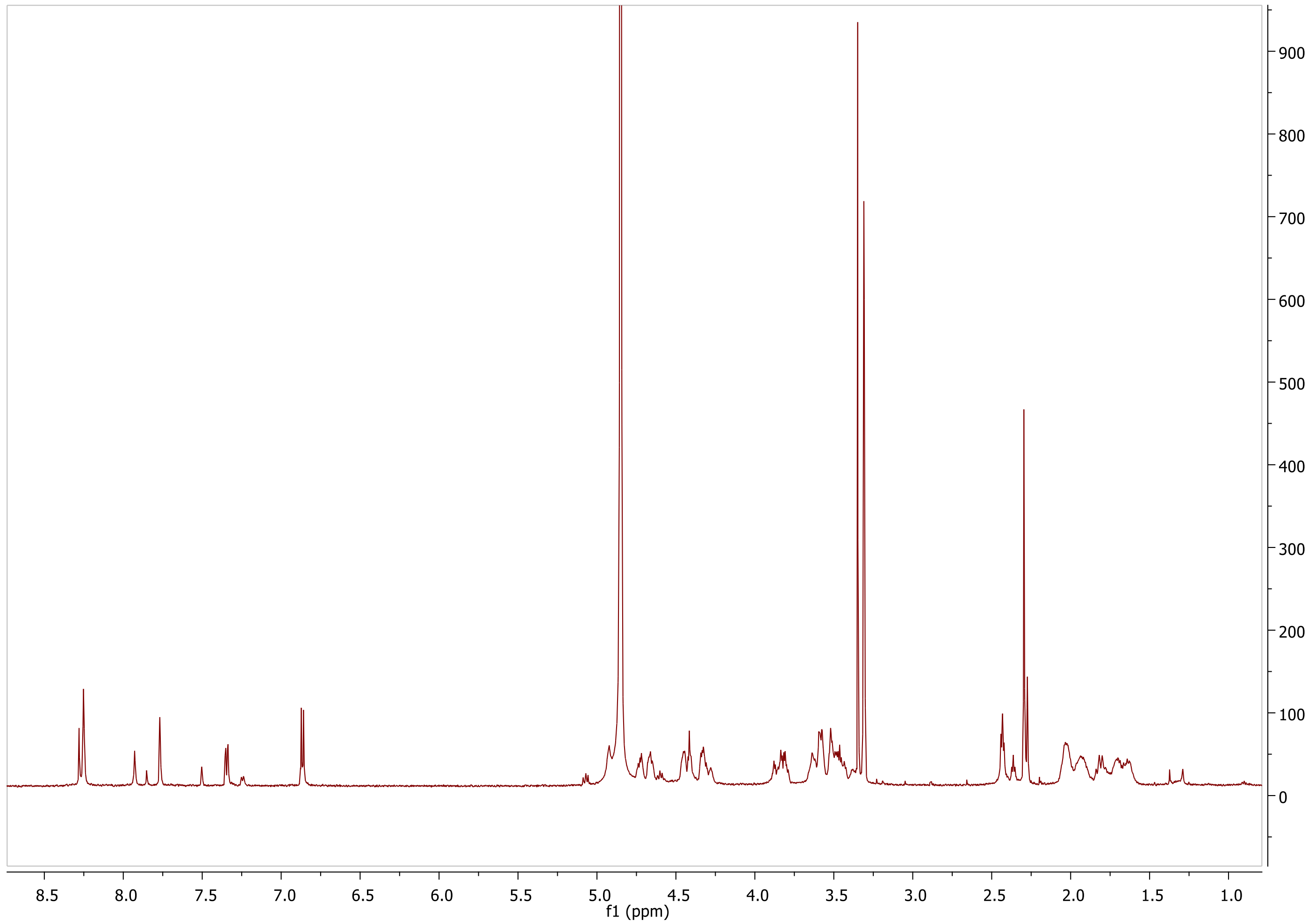

Supplementary Figure 34: ^{1}H NMR (600 MHz, CD_3OD) spectrum of cahuitamycin E (**5**).

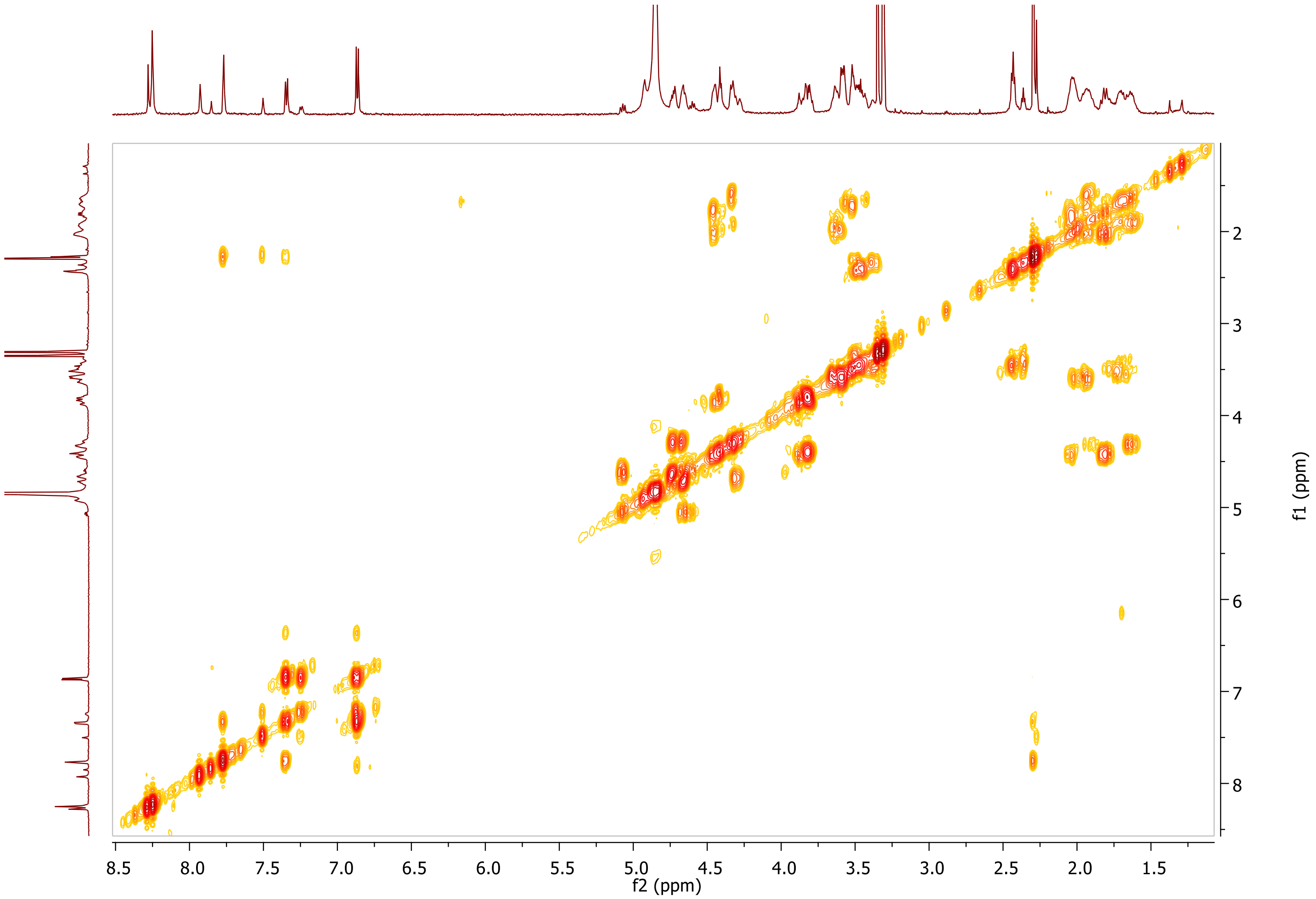

Supplementary Figure 35: ^{1}H-^{1}H COSY NMR spectrum of cahuitamycin E (**5**).

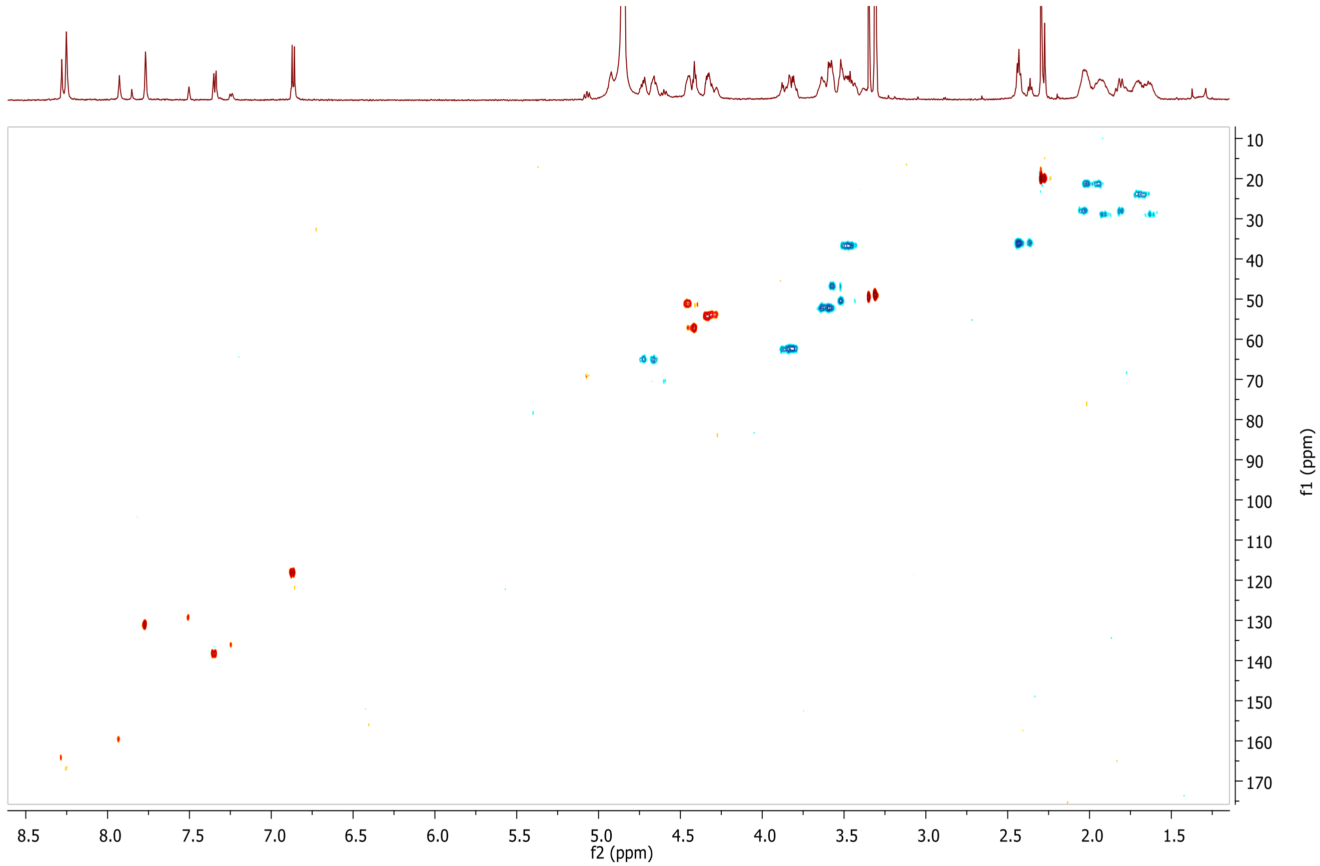

Supplementary Figure 36: 2D HSQC NMR spectrum of cahuitamycin E (**5**).

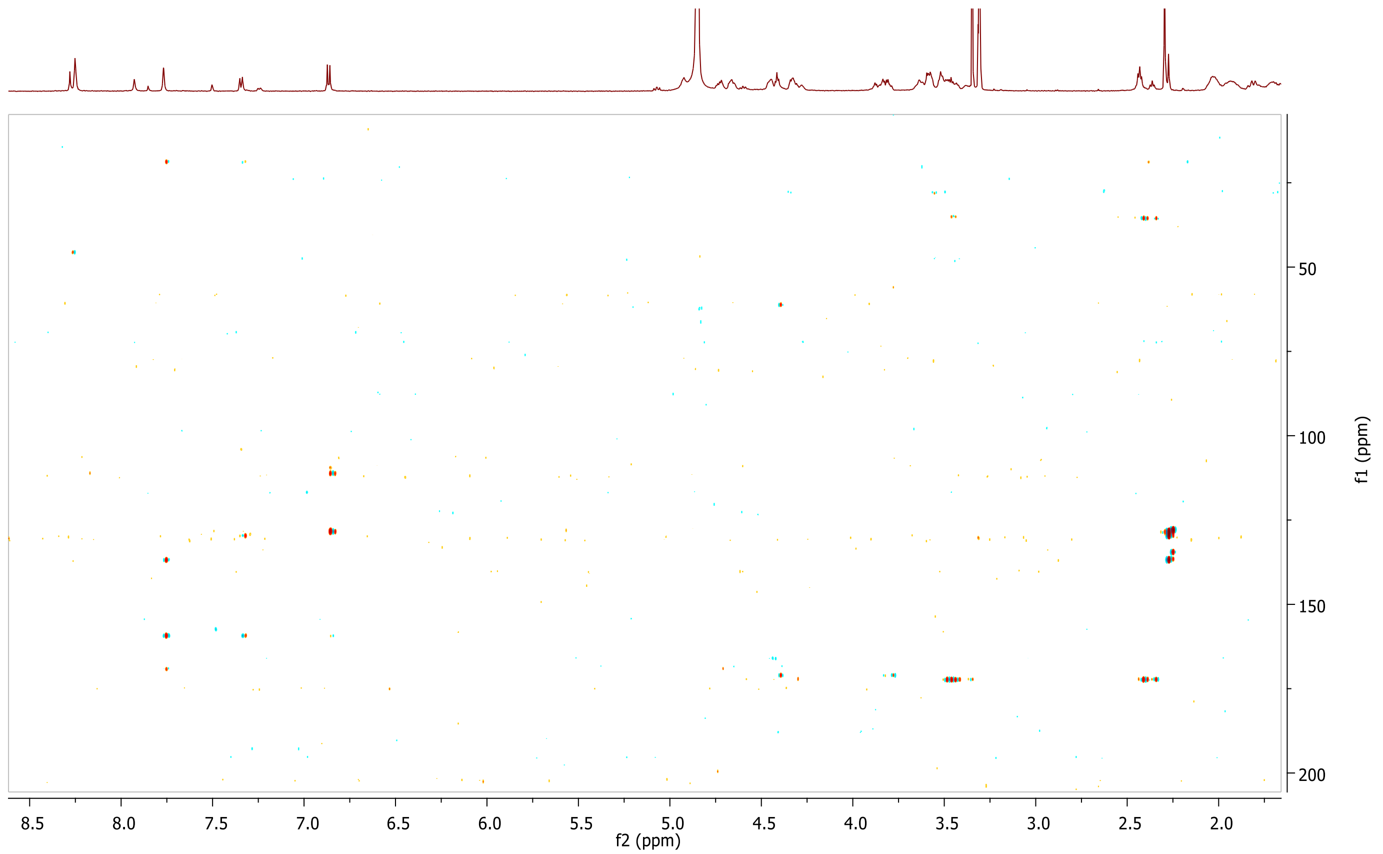

Supplementary Figure 37: 2D HMBC NMR spectrum of cahuitamycin E (**5**).

(a)

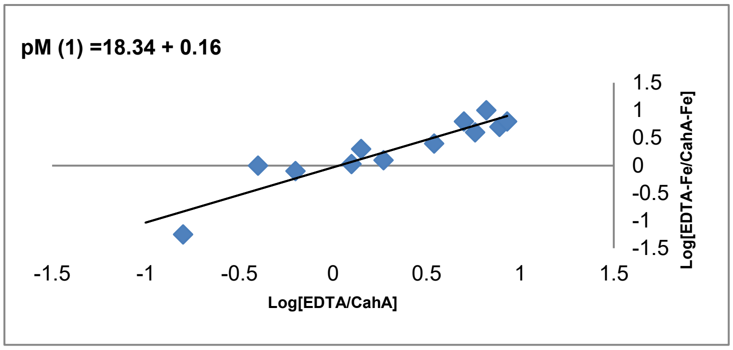

(b)

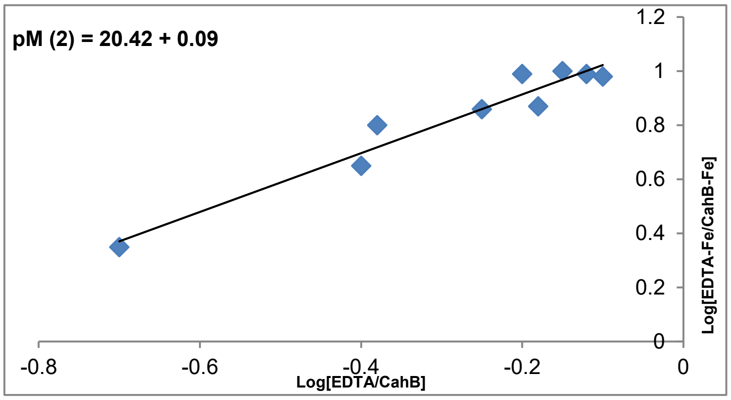

(c)

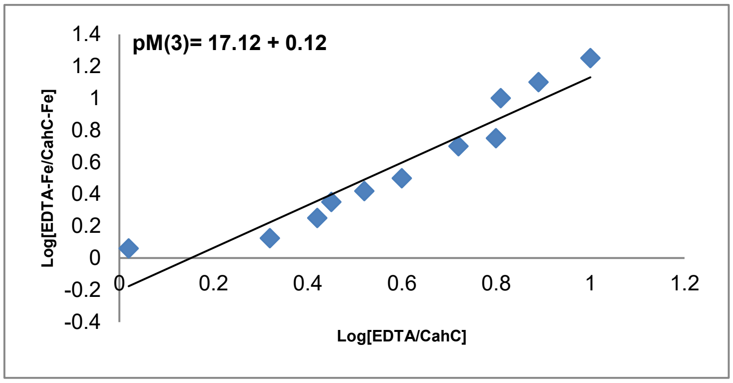

Supplementary Figure 38: Plots of log[EDTA/[Cah] against log [Fe-EDTA]/Fe-Cah] for the ligand; **(a)** [Cah A (**1**)•Fe^{III}]$^{3-}$, **(b)** [Cah B (**1**)•Fe^{III}]$^{3-}$, **(b)** [Cah C (**1**)•Fe^{III}]$^{3-}$. The shown data points are the result of three independent experiments. The data was fitted using linear regression analysis as described in reference[1]

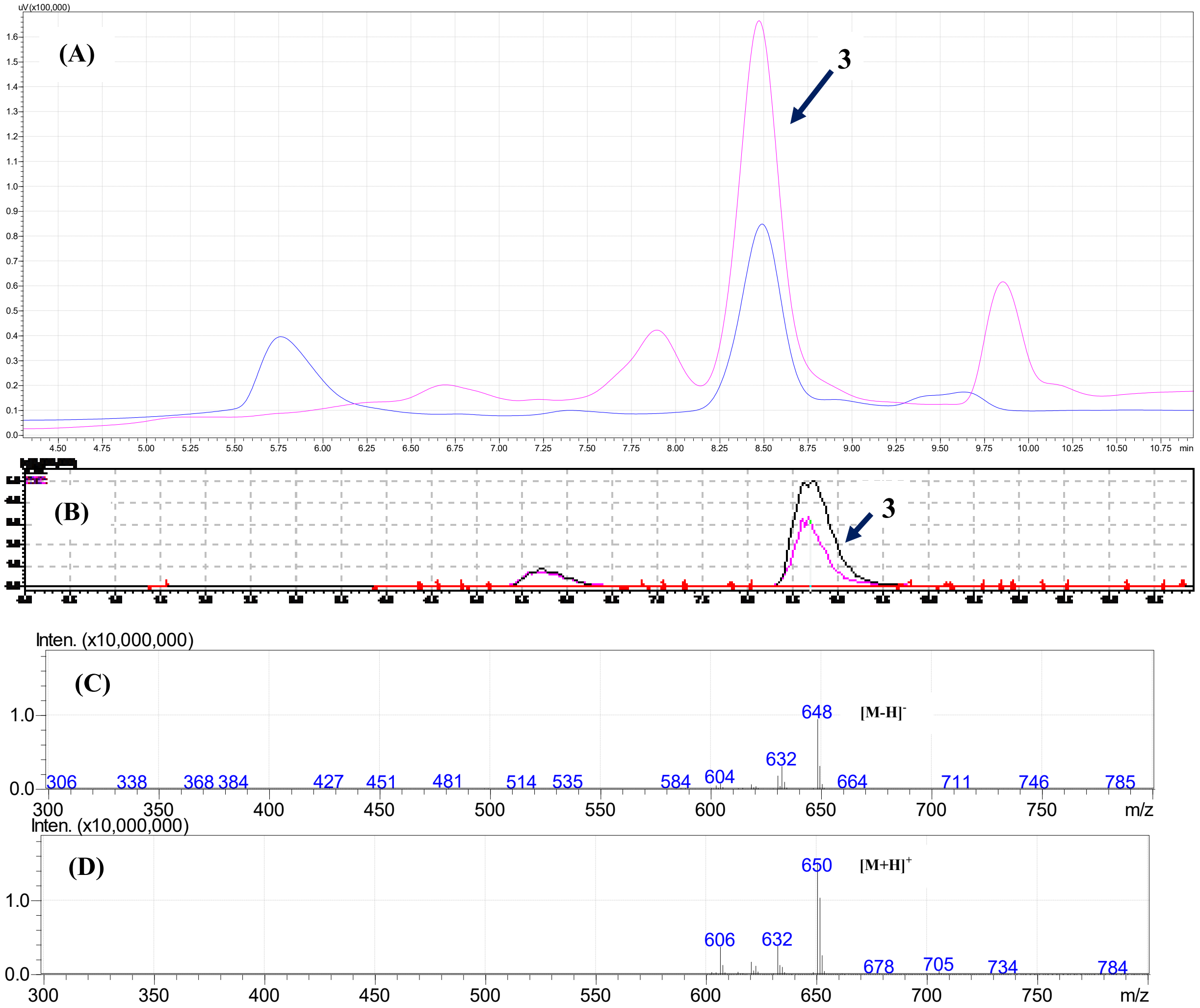

Supplementary Figure 39: Increase in production of cahuitamycin C (**3**) through exogenous feeding of 6-methylsalicylic acid (blue = DHS334 non-fed; Pink = DHS334 fed). (A): UV chromatogram of **3** (X axis- retention time; Y axis- relative abundance); (B) Total Ion Chromatogram of **3** (X axis- retention time; Y axis- relative abundance); (**C**) Observed *m/z* in positive mode (X axis- *m/z*; Y axis- relative intensity); (**D**): Observed *m/z* in negative mode (X axis- *m/z*; Y axis- relative intensity).

Supplementary Figure 40: Mutasynthetic analysis for the generation of new cahuitamycin analogs through LCMS (insets in chromatogram shows the fed substrate to replace **R** in cahuitamycins). All the panels below shows extracted ion chromatogram of expected molecules generated through feeding experiments. (X axis- retention time; Y axis- relative abundance).

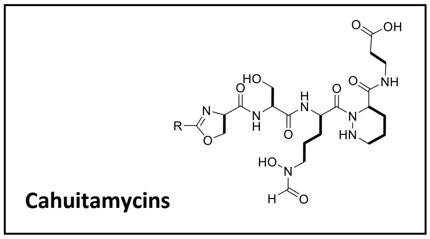

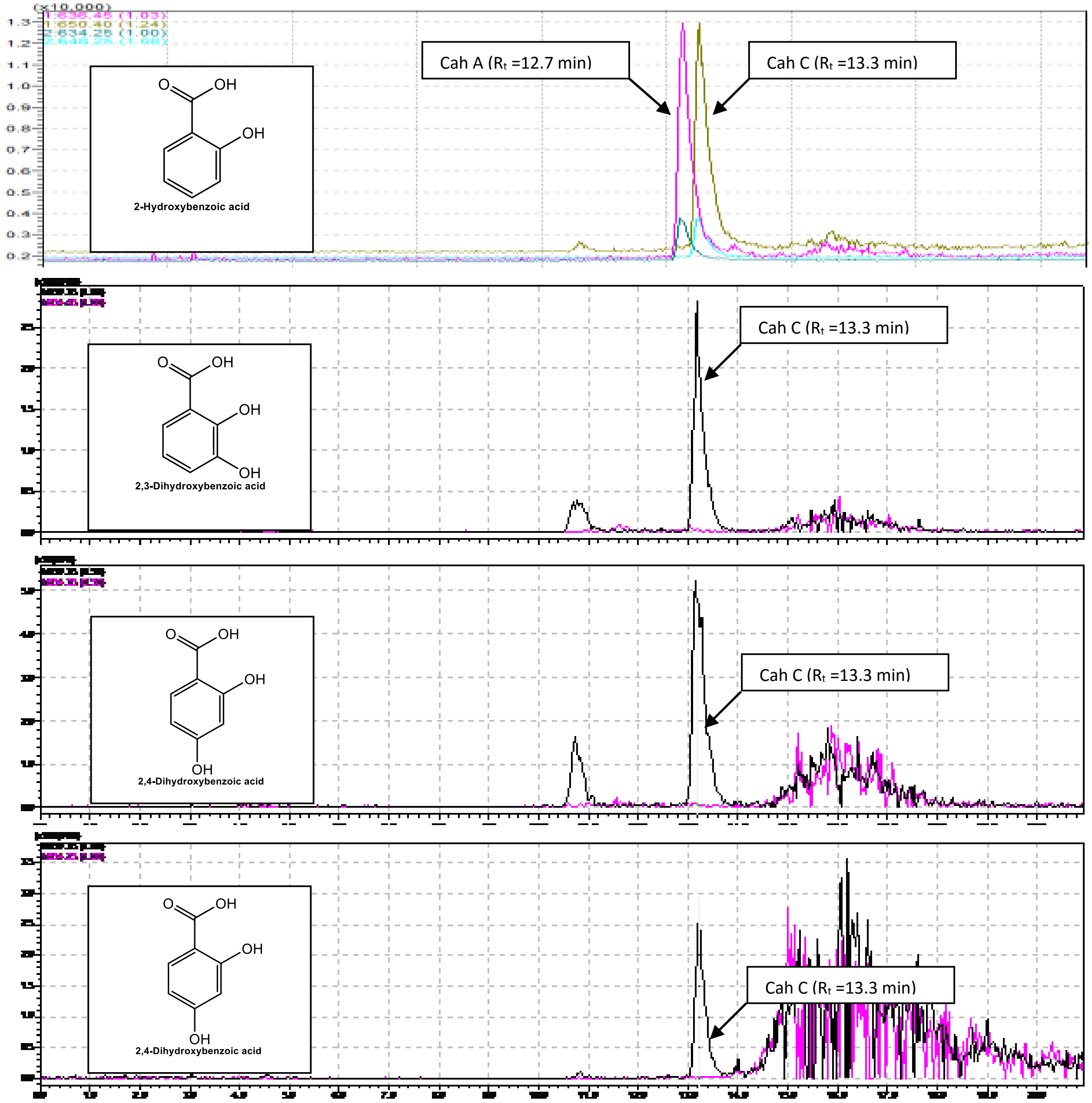

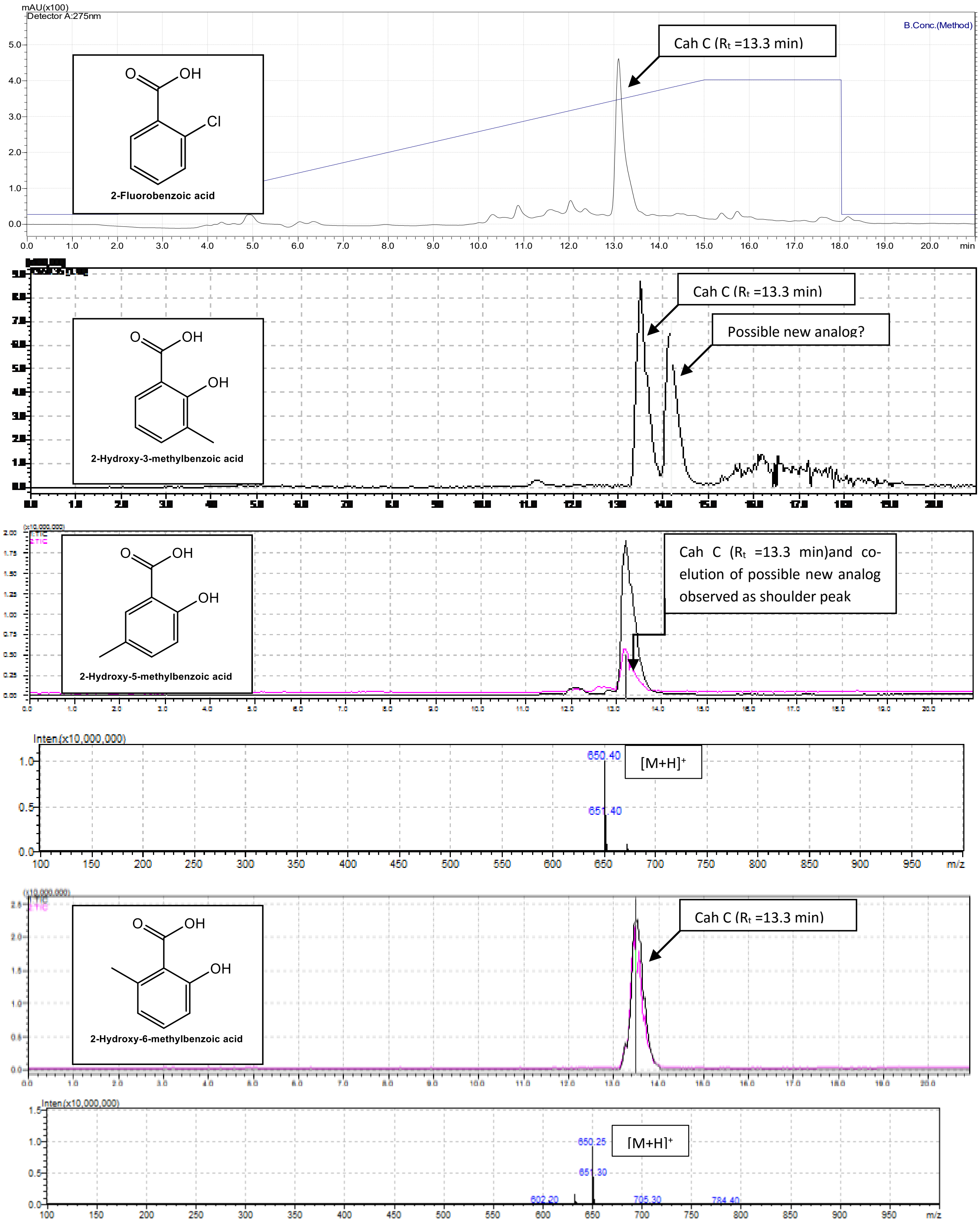
mAU(x100)
Detector A:275nm
B.Conc.(Method)
Cah C (Rt =13.3 min)
2-Fluorobenzoic acid
Cah C (Rt =13.3 min)
Possible new analog?
2-Hydroxy-3-methylbenzoic acid
Cah C (Rt =13.3 min)and co-elution of possible new analog observed as shoulder peak
2-Hydroxy-5-methylbenzoic acid
Inten.(x10,000,000)
650.40
651.40
[M+H]+
Cah C (Rt =13.3 min)
2-Hydroxy-6-methylbenzoic acid
Inten.(x10,000,000)
650.25
651.30
602.20
705.30
784.40
[M+H]+

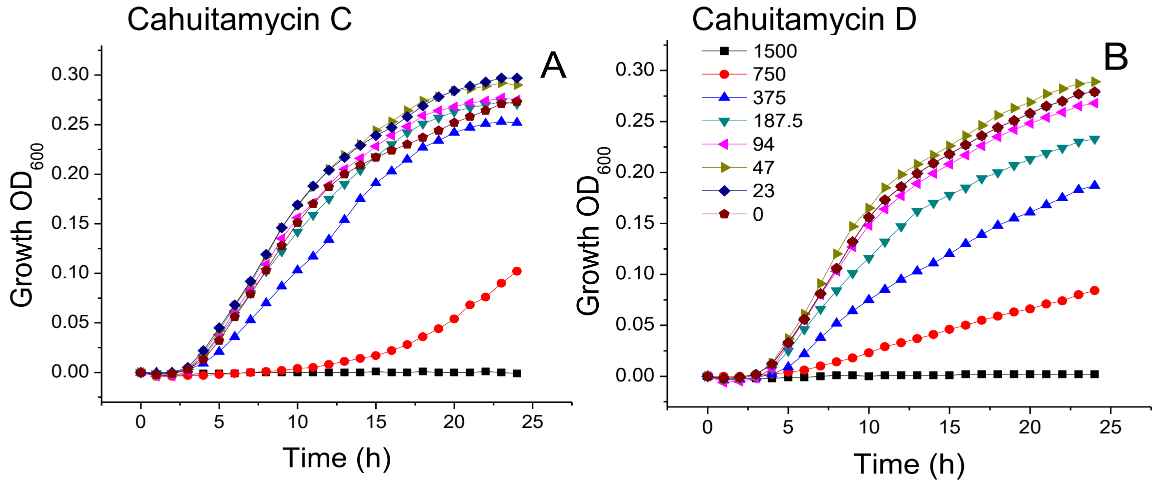

Supplementary Figure 41. Growth curve of *A. baumannii* by cahuitamycin C-D (**3-4**). The unit of tested compounds concentration is in μg/ml.

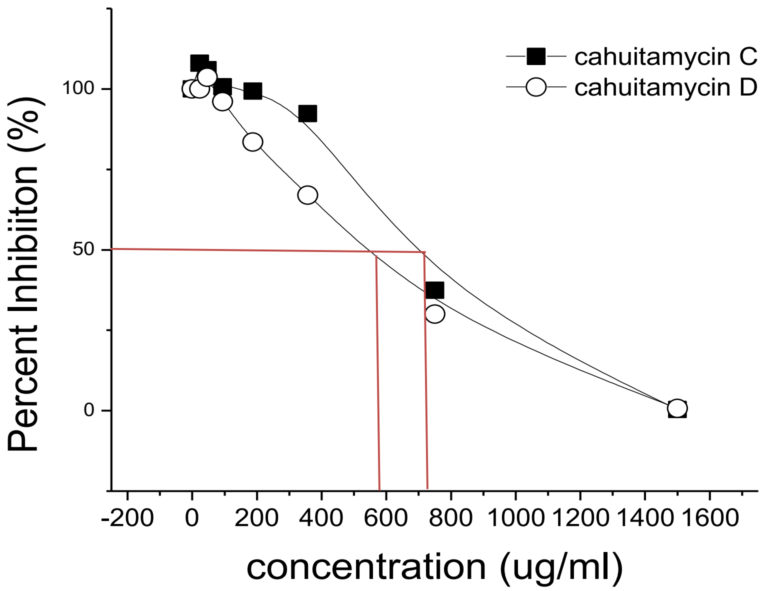

Supplementary Figure 42. Graph showing 50% growth reduction ($IC_{50} \geq 1$ mM: shown with red lines) of *A. baumannii* against cahuitamycins C-D (**3-4**).

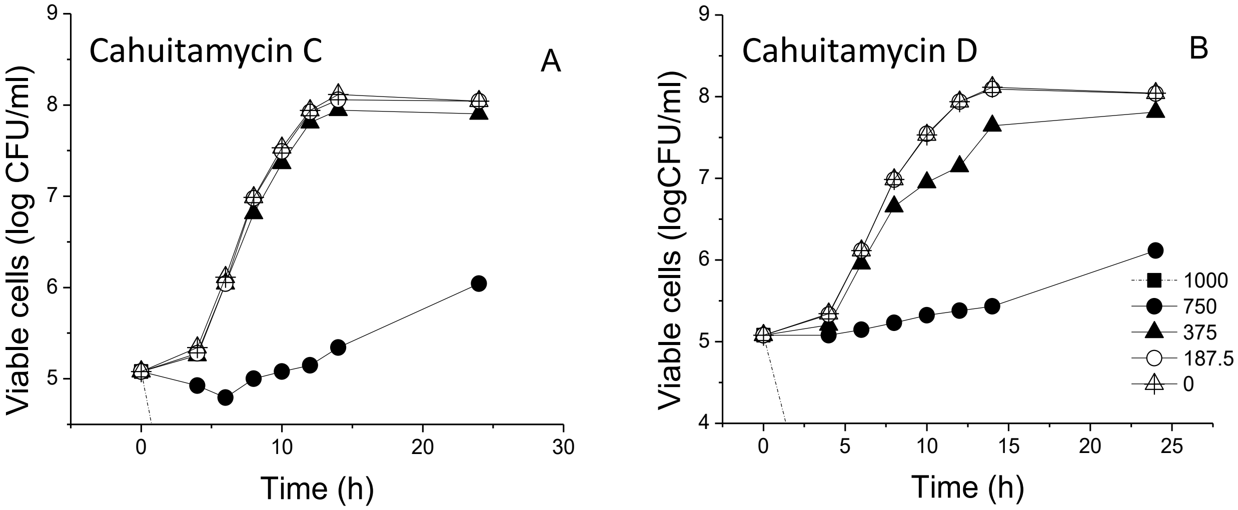

Supplementary Figure 43. Minimum inhibitory concentrations (MICs) of cahuitamycins C-D (**3**-**4**). The unit of tested compounds concentration is in μg/ml.

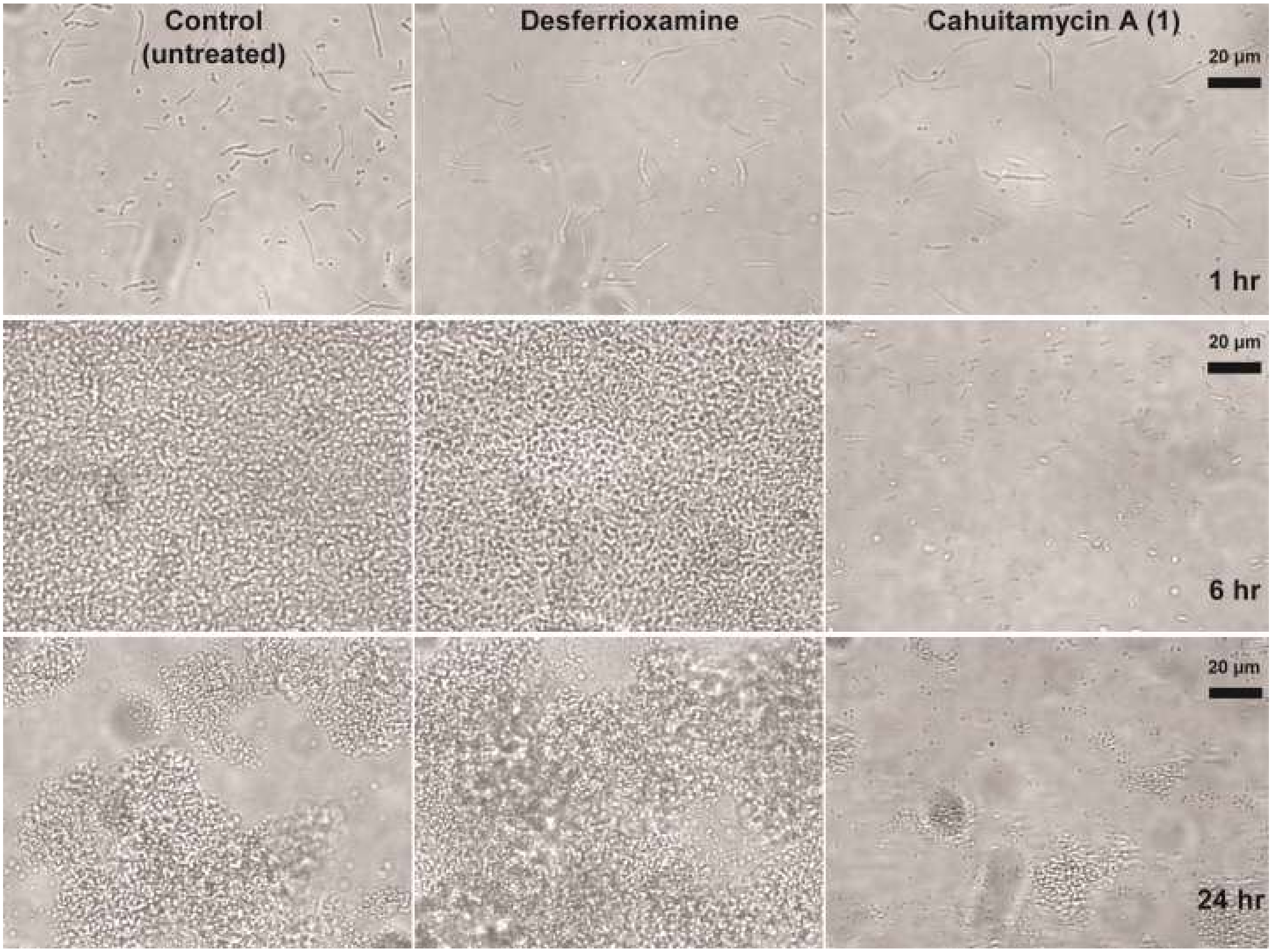

Supplementary Figure 44. Representative micrograph of *A. baumannii* biofilm formation in flow cells. Biofilm biomass was measured by crystal violet method, and normalized by the biomass of control biofilm (no compounds added group). Data were presented in mean value of three replicates. The concentration of compounds in flow media was 7.5 µg/ml and flow rate was 4 ml/hr.

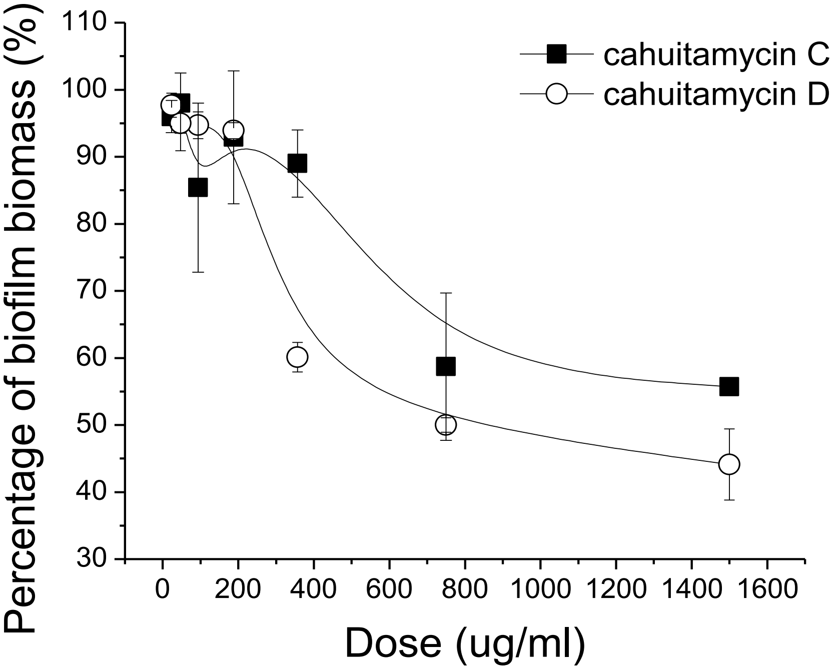

Supplementary Figure 45. Disruption of pre-formed *A. baumannii* biofilm by cahuitamycins C-D (**3**-**4**). Biofilm biomass was obtained by measuring absorbance of crystal violet assay at 600 nm. Results are the average of three replicates ± SD. Student's *t* test was used for statistical analysis, *** indicates $P < 0.001$ compared with control (no addition of compounds).

	Cahuitamycin A (1)				Cahuitamycin B (2)		Cahuitamycin C (3)	
	δ_C	δ_H, multi (*J* in Hz)	COSY	HMBC	δ_C	δ_H, multi (*J* in Hz)	δ_C	δ_H, multi (*J* in Hz)
**1**	174.1				171.1		173.9	
**2**	36.3	2.39, m	3	1	35.2	2.26, m	36.7	2.36, m
								2.39, m
**3**	37.1	3.39, m	2	1	35.4	3.24, m	37.2	3.37, m
		3.47, m	2	4				3.58, m
**4**	167.3				167.2		173.4	
**5**	51.2	4.41, dd (4.4, 9.5)	6	4, 9	52.4	4.17, dd (4.0. 9.1)	51.4	4.47 dd (4.2, 9.0)
**6**	27.8	1,76, m	5, 7		27.6	1.6, m	28.4	1.81, m
		1.98, m	5, 7			1.85, m		2.01, m
**7**	21.3	1.91, m	6, 8	5	20.2	1.83, m	21.8	1.93
		1.97, m	6, 8			1.88, m		1.99
**8**	52.5	3.57, m	7	9	50.9	3.45, m	52.5	3.57, m
		3.63, m	7	6				3.60, m
**9**	173.9				170.2		173.8	
**10**	54.4	4.29, dt (6.1, 14.2)	11	9, 11, 15	55.3	4.25, dt (5.8, 14.0)	54.4	4.33, dt (6.0, 14.0)
**11**	29.2	1.61, m	12, 10		28.9	1.45, m	29.5	1.63, m
		1.82, m	10			1.66, m		1.89, m
**12**	24.03	1.61, m	11, 13	13, 14, 10	22.6	1.45, m	24.4	1.66, m
		1.68, m	11, 13	14, 10		1.51, m		
**13**	50.9	3.45, t (6.4, 13.1)	12	11, 14	48.5	3.29, t (6.4. 12.7)	50.8	3.45, t (6.3, 13.0)
**14**	164.1	8.21, s			161.6	8.21, s	163.9	8.25, s
**15**	172.3				170.3		172.3	
**16**	57.1	4.48, m	17	15, 18	55.4	4.56, m	57.3	4.47, m
**17**	62.4	3.89, d (5.1)	16	15	61.5	3.58, m	63.1	3.89, m
						3.63, m		
**18**	173.6				169.8		174.01	
**19**	68.8	5.11, dd (7.8, 10.3)	20	18	55.2	4.52, m	68.1	5.05, dd (7.8, 10.2)
**20**	70.4	4.61, dd (7.8, 9.3)	19	18, 21	61.7	3.69, m	70.8	4.65, dd (7.8, 9.0)
		4.69, dd (8.1, 9.3)	19	21		3.73, m		
**21**	168.6				164.8		170.5	
**22**	133.8				133.3		112.8	
**23**	129.6	7.71, d (8.4)	24	25, 27	129.4	7.34, d (8.0)	141.2	
**24**	120.6	6.96, t (8.4, 14.8)	23, 25	22, 23	116.7	6.91, t (8.0, 14.0)	22.7	2.51, s
**25**	135.4	7.48, t (7.4, 14.8)	24, 26	23, 27	116.8	6.81, t (7.0, 14.0)	123.1	6.73, d (6.7)
**26**	117.5	7.01, d (7.4)	25	22, 24	117.4	7.91, d (7.0)	133.7	7.24, t (6.7, 7.4)
**27**	159.7				156.9		115.6	6.81, d (6.7)
**28**							157.8	

Supplementary Table 1. NMR spectroscopic data for cahuitamycins A-C (**1-3**)

	Cahuitamycin D (4)				Cahuitamycin E (5)	
	δ_C	δ_H, multi (J in Hz)	COSY	HMBC	δ_C	δ_H, multi (J in Hz)
**1**	174.3				173.2	
**2**	36.4	2.36, m	3	1	36.2	2.36, m
		2.41, m				2.41, m
**3**	36.9	3.39, m	2	1	36.7	3.42, m
		3.50, m	2	4		
**4**	167.3				170.4	
**5**	51.1	4.41, dd (4.5, 9.5)	6	4, 9	54.2	4.33, dd (4.0. 9.0)
**6**	27.8	1,72, m	5, 7		28.7	1.63, m
		2.01, m	5, 7			1.93, m
**7**	21.3	1.91, m	6, 8	5	21.2	1.73, m
		1.97, m	6, 8			1.88, m
**8**	52.1	3.51, m	7	9	50.6	3.45, m
		3.60, m	7	6		
**9**	173.7				172.2	
**10**	54.6	4.30, dt (6.0, 14.0)	11	9, 11, 15	54.3	4.36, dt (5.8, 14.0)
**11**	29.4	1.61, m	12, 10		28.9	1.58, m
		1.82, m	10			1.77, m
**12**	24.1	1.63, m	11, 13	13, 14, 10	24.3	1.60, m
		1.70, m	11, 13	14, 10		1.71, m
**13**	51.1	3.45, t (6.4, 13.0)	12	11, 14	52.3	3.59, m
						3.63, m
**14**	165.6	8.21, s			164.6	8.28, s
**15**	172.3				171.7	
**16**	56.9	4.42, m	17	15, 18	54.3	4.32, m
**17**	62.4	3.89, d (5.0)	16	15	62.8	3.85, m
		3.80, m				3.91, m
**18**	173.2				172.4	
**19**	69.2	5.07, dd (7.5, 10.0)	20	18	69.1	5.08, dd (7.0, 9.8)
**20**	70.2	4.60, dd (7.5, 9.0)	19	18, 21	65.1	4.66, m
		4.66, dd (8.0, 9.0)	19	21		4.72, m
**21**	170.1				170.7	
**22**	122.6				121.4	
**23**	130.8	7.78, s	24	25, 27	131.2	7.77, s
**24**	129.4				127.8	
**25**	20.3	2.30, s	23, 24, 26		20.3	2.31
**26**	138.4	7.35, d (7.0)	24, 26	23, 27	138.4	7.31, d (7.0)
**27**	117.3	6.87, d (7.0)	25	22, 24	118.2	6.87, d (7.0)
**28**	159.7				157.2	

Supplementary Table 2. NMR spectroscopic data for cahuitamycins D (**4**) and E (**5**)

Mutation in *rpsL*	Amino acid substitution	Number of mutants with same mutation
130A→G	Lys43→Arg	1/8
244G→A	Val82→Met	6/8*
271C→T	Pro91→ Thr	1/8

* A mutant of *S. gandocaensis* with V82M mutation in *rpsL* (DHS287) has been used in this study

Supplementary Table 3. Locations of putative *rpsL* gene mutations and resulting amino acid changes in *S. gandocaensis*

Protein	Amino acid	Proposed function	Sequence similarity, Organism	Identity/ Similarity (%)	GenBank accession no.
**CahFT**	317	Formyltransferase	SCAB_85511, *Streptomyces scabies*	81/90	CBG75496
**CahMO**	451	L-ornithine-5-monooxygenase	STRTUCAR8_09254, *Streptomyces turgidiscabies Car8*	72/81	ELP64051
**CahT1**	338	Putative iron(III)/siderophore ABC transporter substrate binding protein	FhuD, *Actinoplanes missouriensis* 431	36/57	BAL90510
**CahA**	1159	NRPS (T Cy A T)	NRPS, *Streptomyces viridochromogenes*	82/87	ELS51167
**CahB**	2626	NRPS (C A T C A T E)	NRPS, *Streptomyces viridochromogenes*	80/85	ELS51166
**CahC**	1503	NRPS (C A T E)	NRPS, *Rhodococcus opacus*	45/57	BAH53136
**CahD**	1057	NRPS (C A T)	NRPS*, Streptomyces* sp. NRRL F-4415	51/62	AGE11899
**CahE**	72	MbtH-like protein	MbtH, *Streptomyces viridochromogenes*	82/92	ELS51232
**CahF**	162	Aspartate 1-decarboxylase	PanD, *Amycolatopsis mediterranei* U32	78/84	ADJ46798
**CahG**	270	Alpha-beta hydrolase	LipE*, Streptomyces albus* J1074	64/76	EFE82037
**CahH**	424	Siderophore export protein	SACE_2690, *Saccharopolyspora erythraea* NRRL 2338	64/75	CAM01972
**CahI**	425	Salicylate synthase	MbtI, *Mycobacterium tuberculosis* UT205	43/57	CCE37856
**CahJ**	544	Salicylate-AMP ligase	MxcE, *Sorangium cellulosum* So ce56	64/74	CAN94040
**CahT2**	323	Iron ABC transporter permease	STVIR_7837, *Streptomyces viridochromogenes*	89/92	ELS51226
**CahT3**	353	Iron ABC transporter permease	STVIR_7836, *Streptomyces viridochromogenes*	88/94	ELS51225
**ORF1**	595	FAD/iron sulfur cluster binding oxidoreductase	B479_00040, *Pseudomonas putida* HB3267	36/49	AGA70926
**ORF2**	361	Peptidase C45, acyl-coenzyme A/6-aminopenicillanic acid acyl-transferase	SACE_5325, *Saccharopolyspora erythraea* NRRL 2338	51/62	CAM04564
**ORF3**	344	6-aminohexanoate-oligomer hydrolase	Ny1C, *Flavobacterium* sp.	60/73	BAA01528
**CahT4**	251	ABC transporter ATPase	OppF, *Agromyces* sp. KY5R	60/71	BAE97623
**CahT5**	329	ABC transporter ATPase	Vapar_6007, *Variovorax paradoxus*	51/65	ACS22576
**CahT6**	286	ABC transporter permease	OppC, *Agromyces* sp. KY5R	69/83	BAE97625
**CahT7**	323	ABC transporter permease	OppB, *Agromyces* sp. KY5R	56/76	BAE97626
**CahT8**	535	Extracellular solute-binding protein	OppA, *Agromyces* sp. KY5R	52/70	BAE97627
**CahR**	333	LacI-family transcriptional regulator	SSEG_04729, *Streptomyces sviceus* ATCC29083	74/83	EDY58149
**CahMASA**	1760	6-methylsalicylic acid synthase	ChlB1, *Streptomyces antibioticus*	56/78	AAZ77673

Supplementary Table 4. Deduced function of ORFs in the cahuitamycin biosynthetic gene cluster

A domain	active site residues	predicted substrate
CahA-A	D L Y N L G L I H K	L-Cys
CahB-A_1	D V W H V S L V D K	L-Ser
CahB-A_2	D I N Y W G G I G K	L-fhOrn
CahC-A	D A W E G G L V D K	L-Gln
CahD-A	I D V T I S L A D K	L-β-Ala

Supplementary Table 5. Substrate specificity prediction for the A domains of the Cah NRPS based on the specificity-conferring codes of A-domains using a web-based PKS/NRPS analysis tool.[2]

Primer	Sequences 5’ to 3’	Description
SR144	GCGCACCGTACGTCTCGAGGAATTCGGGCTCAGTCCGAAGCAG	Forward and reverse primers for amplification of 5' flanking region of *cahI*
SR145	TCTGGGGTTCGGGGAGAAACTGCGCAGCGTC	
SR146	CGGTATCAGGGAACAATCCCGACTCCGCC	Forward and reverse primers for amplification of 3' flanking region of *cahI*
SR147	CCGGTGACGTCACCATGGGAAGCTTCGCTGTCGAAGCCGCAGAC	
SR148	GCAGTTTCTCCCCGAACCCCAGAGTCCC	Forward and reverse primers for amplification of kanamycin resistance gene
SR149	GGGATTGTTCCCTGATACCGCTCGCCGC	
SR185	TTGCCGTTCAGCAGCACCTTG	Primers for amplifying the *rpsL*
SR186	TTCCAGGTTAGCTGTACACAT	
SR233	ACTTCTCCCAGACGCACG	Primers for verifying the Δ*cahI* mutant by PCR
SR234	TAACCTAAGTCCAGGGAG	
SR235	GCCGCGCGGCAGCCATATGCTCGATGGGTGGGTG	Forward and reverse primers for amplification of *cahJ*
SR236	TCGAGTGCGGCCGCAAGCTTCAGTGGACACCGCCGTC	
FC27	5’-AGAGTTTGATCCTGGCTCAG-3’	Forward and reverse primers for amplification of 16s rDNA of *S. gandocaensis*
RC1492	5’-TACGGCTACCTTGTTACGACTT-3’)	

Supplementary Table 6. Oligonucleotide primers used in this study.

Strains and plasmids	Description	Source of reference
Strains		
S. gandocaensis	Wild-type cahuitamycin producer	This study
DHS287	Ribosome-engineered mutant of *S. gandocaensis*	This study
DHS334	Δ*cahI S. gandocaensis*	This study
ET12567	methylation-deficient *Escherichia coli*	[ref][3]
Plasmids		
pKC1139	*E.coli-Streptomyces* shuttle vector for gene inactivation	[ref][4]
pSRP50	*cahI* deletion plasmid	This study
pYJ276	Plasmid for PCR amplification of kanamycin resistance gene	[ref][5]

Supplementary Table 7. Strains and plasmids used in this study

Reference

1. Abergel RJ, Zawadzka AM, Raymond KN. Petrobactin-Mediated Iron Transport in Pathogenic Bacteria: Coordination Chemistry of an Unusual 3,4-Catecholate/Citrate Siderophore. *J Am Chem Soc* **130**, 2124-2125 (2007).

2. Bachmann BO, Ravel J. Methods for in silico prediction of microbial polyketide and nonribosomal peptide biosynthetic pathways from DNA sequence data. *Meth Enzymol* **458**, 181-217 (2009).

3. MacNeil DJ, Gewain KM, Ruby CL, Dezeny G, Gibbons PH, MacNeil T. Analysis of Streptomyces avermitilis genes required for avermectin biosynthesis utilizing a novel integration vector. *Gene* **111**, 61-68 (1992).

4. Bierman M, Logan R, O'brien K, Seno E, Rao RN, Schoner B. Plasmid cloning vectors for the conjugal transfer of DNA from *Escherichia coli* to *Streptomyces spp*. *Gene* **116**, 43-49 (1992).

5. Jung WS*, et al.* Enhanced heterologous production of desosaminyl macrolides and their hydroxylated derivatives by overexpression of the pikD regulatory gene in Streptomyces venezuelae. *Applied and environmental microbiology* **74**, 1972-1979 (2008).
